# Adjustment with aphasia after stroke: study protocol for a pilot feasibility randomised controlled trial for SUpporting wellbeing through PEeR Befriending (SUPERB)

**DOI:** 10.1186/s40814-019-0397-6

**Published:** 2019-01-22

**Authors:** Katerina Hilari, Nicholas Behn, Jane Marshall, Alan Simpson, Shirley Thomas, Sarah Northcott, Chris Flood, Sally McVicker, Mireia Jofre-Bonet, Becky Moss, Kirsty James, Kimberley Goldsmith

**Affiliations:** 1 Centre for Language and Communication Science Research, School of Health Sciences, City, University of London, Northampton Square, London, EC1V 0HB UK; 2 Centre for Mental Health Research, School of Health Sciences, City, University of London, Northampton Square, London, EC1V 0HB UK; 3 Division of Rehabilitation and Ageing, School of Medicine, Floor B, The Medical School, Queen’s Medical Centre, University of Nottingham, Nottingham, NG7 2UH UK; 4 Aphasia Re-Connect at City, Centre for Language and Communication Science Research, School of Health Sciences, City, University of London, Northampton Square, London, EC1V 0HB UK; 5 Department of Economics, School of Arts and Social Sciences, City, University of London, Northampton Square, London, EC1V 0HB UK; 6 Biostatistics & Health Informatics Department, Division of Psychology and Systems Sciences, Institute of Psychiatry, Psychology and Neuroscience, King’s College London, De Crespigny Park, London, UK

**Keywords:** Stroke, Aphasia, Peer support, Peer befriending, Randomised controlled trial, Feasibility, Behavioural intervention

## Abstract

**Background:**

Despite the high prevalence of mood problems after stroke, evidence on effective interventions particularly for those with aphasia is limited. There is a pressing need to systematically evaluate interventions aiming to improve wellbeing for people with stroke and aphasia. This study aims to evaluate the feasibility of a peer-befriending intervention.

**Methods/design:**

SUPERB is a single blind, parallel group feasibility trial of peer befriending for people with aphasia post-stroke and low levels of psychological distress. The trial includes a nested qualitative study and pilot economic evaluation and it compares usual care (*n* = 30) with usual care + peer befriending (*n* = 30). Feasibility outcomes include proportion screened who meet criteria, proportion who consent, rate of consent, number of missing/incomplete data on outcome measures, attrition rate at follow-up, potential value of conducting main trial using value of information analysis (economic evaluation), description of usual care, and treatment fidelity of peer befriending. Assessments and outcome measures (mood, wellbeing, communication, and social participation) for participants and significant others will be administered at baseline, with outcome measures re-administered at 4 and 10 months post-randomisation. Peer befrienders will complete outcome measures before training and after they have completed two cycles of befriending. The qualitative study will use semi-structured interviews of purposively sampled participants (*n* = 20) and significant others (*n* = 10) from both arms of the trial, and all peer befrienders to explore the acceptability of procedures and experiences of care. The pilot economic evaluation will utilise the European Quality of life measure (EQ-5D-5 L) and a stroke-adapted version of the Client Service Receipt Inventory (CSRI).

**Discussion:**

This study will provide information on feasibility outcomes and an initial indication of whether peer befriending is a suitable intervention to explore further in a definitive phase III randomised controlled trial.

**Trial registration:**

ClinicalTrials.gov identifier NCT02947776, registered 28th October 2016.

**Electronic supplementary material:**

The online version of this article (10.1186/s40814-019-0397-6) contains supplementary material, which is available to authorized users.

## Background

Stroke and aphasia can have a profound impact on people’s lives. Depression is a common sequel of stroke, with rates remaining high even 1 year later at 33% [1]. Depression is associated with worse rehabilitation outcomes, increased carer strain, increased healthcare utilisation, and higher mortality [2–4]. Yet, the Stroke Association’s report ‘Feeling overwhelmed’ [5] highlights that over half of stroke units in England still have no access to psychology services; and that two thirds of stroke survivors felt their emotional needs were not as well looked after as their physical needs.

There is evidence that the psychological needs of people with aphasia are even greater than in the general stroke population. One study reported a 62% rate of depression in this group 1 year post-stroke [6] and family of people with aphasia post-stroke often fall under strain [7]. Social support and social networks are also affected [8]. People with aphasia take part in fewer social activities [9] and are at risk of losing contact with friends and their wider social network [10, 11]. This is particularly concerning, as poor social support is associated with worse physical recovery [12] and increased likelihood of a future adverse event such as a second stroke [13]. It is therefore paramount to support psychosocial well-being post stroke and aphasia.

A UK audit of clinical psychology services for people with mood problems post-stroke across ten stroke services found that the most common outcome of mood assessment was monitoring and advice, with less than half of patients identified as having low mood receiving psychological intervention [14]. The National Clinical Guideline for Stroke [15] highlights that psychological care after stroke should be multifaceted, involving many agencies—health, social care, voluntary. It recommends that services for people with stroke should offer psychological support to *all* patients regardless of whether they exhibit specific mental health or cognitive difficulties and use a matched care model to select the level of support appropriate to the person’s needs. A Cochrane systematic review on the effectiveness of psychological therapies for treating post-stroke depression identified that most studies excluded people with aphasia [16]. There is a pressing need to systematically evaluate interventions that aim to improve psychosocial wellbeing for people with stroke and for the vulnerable group of people with aphasia in particular.

The proposed study aims to address this need for people with aphasia with no or low levels of psychological distress. A systematic review of rehabilitation interventions to prevent and treat depression specifically in post-stroke aphasia found that though some interventions may enhance mood for those without clinically significant depression, they do not lead to significant reductions in depression scores [17]. Evidence of benefit for those with no or low mood problems would be particularly welcome, as these interventions might help to avert some of the long-term psychological consequences of stroke and prevent the need for more complex and possibly more costly psychological therapies.

An intervention with potential for application in the field of stroke and aphasia is one to one peer support/peer befriending. Peer befriending is widely used in mental health [18] and other long-term conditions [19]. Peer befriending is social and emotional support provided by people with experience of a condition to others sharing a similar condition to bring about a desired social or personal change [20]. Peer befriending has been evaluated in stroke but only within a hospital setting and not for those with aphasia [21]. Peer befrienders, who have achieved improvements in their own condition, have been found to offer acceptance, respect, empathy, support, companionship, and hope and share experiences and ideas about how to cope [22]. A recent systematic review and meta-analysis found moderate but significant positive effects of befriending on depressive symptoms in depressed elderly people [standard difference in means SMD = − 0.75] [23].

In the UK, a charity for people with aphasia (formerly Connect—the communication disability network, now Re-Connect) offers a peer-befriending scheme. The scheme has mostly targeted those with aphasia in the longer term post-stroke who are socially isolated with poor access to support systems. Positive outcomes have been reported for people with aphasia taking part in the befriending scheme, their families, and health professionals involved in their care [24]. The current study aims to answer feasibility questions about a refined version of the peer befriending scheme that is offered to people with aphasia post-stroke earlier, i.e. when they are discharged to the community from hospital, active care is withdrawn, and they have an increased need for support [25].

### Objectives

This study aims to provide answers to questions about the feasibility of a definitive phase III randomised controlled trial (RCT) of the clinical and cost-effectiveness of peer befriending for people with aphasia post-stroke. These questions include the following:

- Feasibility of recruitment and retention to the trial
- Acceptability of research procedures and outcome measures
- Acceptability of usual care + peer befriending compared with usual care control to (a) participants, (b) their significant others, and (c) peer befrienders
- Documentation of usual care
- Treatment fidelity of peer befriending
- Exploration of psychological and social well-being outcomes as outcomes for a definitive trial for (a) people with aphasia receiving usual care + peer befriending versus usual care control, (b) their significant others, and (c) peer befrienders
- Feasibility of a full economic evaluation of usual care + peer befriending versus usual care control.

## Methods/design

The study is a single-blind, mixed methods, parallel group pilot feasibility (phase II) multicentre RCT comparing usual care with usual care + peer befriending for people with aphasia post-stroke and low levels of psychological distress. To ensure all necessary aspects were addressed in the study protocol, the Standard Protocol Items: Recommendations for Interventional Trials (SPIRIT) checklist [26, 27] was adhered to and the Template for Intervention Description and Replication (TIDieR) [28] was used (see Additional files 1 and 2). SPIRIT checklist page numbers refer to the full study protocol (see Additional file 3).

### Participants

#### Setting

This study takes place in North London boroughs. Primary recruitment sites are hospitals. To maximise recruitment and pick up in the community people who may have been missed in the hospital, we also recruited from community services (e.g. Speech and Language Therapy (SLT) teams), and in the second half of the recruitment period, we also recruited from GP practices within the included boroughs. The hospital sites and GP practices were recruited through the North Thames National Institute for Health Research (NIHR) Clinical Research Network (CRN), which has adopted the study into their portfolio, and through the study team’s SLT contacts with services. Hospital sites were excluded if they have active community peer befriending schemes for people with aphasia in place. Baseline assessments and randomisation take place when participants with aphasia are back in the community and, where applicable, have completed intensive rehabilitation care (early supported discharge). For this reason, community SLT services were asked to identify people who had just completed or were close to competing intensive input. GP practices were asked to identify people who had a stroke up to 6 months previously.

#### Participants with aphasia

Inclusion criteria for participants with aphasia are as follows:

- Over 18 years of age.
- Fluent premorbid users of English (confirmed by relative or self-report).
- Presence of aphasia due to stroke: determined by the multidisciplinary team notes, based on SLT diagnosis. In cases of uncertainty, i.e. where there is no SLT diagnosis at the time of recruiting to the trial, CRN nurses will use the Frenchay Aphasia Screening Test [29] for screening for aphasia. In these cases, the presence of aphasia will be determined based on the published cut-offs in the Frenchay Aphasia Screening Test manual.
- Low levels of emotional distress. This aims to ensure that participants do not require more complex psychological interventions. To determine the level of emotional distress, the Depression Intensity Scale Circles (DISCS) [30] will be used. This tool is recommended for people with communication problems, cognitive deficits, and visual perception problems post-stroke [31]. Scores on DISCS range 0–5, with a score of 0–1 indicating no/low distress and a score of ≥ 2 used as a cut-off for identifying depression in those with complex disabilities following brain injury [30]. Those scoring 0–1 will be eligible for the study. If a participant scores ≥ 2 on DISCS, they will be referred back to the multidisciplinary team for consideration for more complex psychological care as appropriate. People who score 2 (which is also the median on DISCS) and who the multidisciplinary team deems do not need other psychological care or other psychological care is not available will still be eligible to take part. If a person scores 3 if screened while still in the hospital, and is uncertain about their level of emotional distress or feels they will be better once home, consent will be obtained to re-screen them for eligibility when they return to the community (second screen). If at the second screen they score 0–1, or 2 as described above, then they will be eligible to take part.

#### Significant others

Each person with aphasia will be invited to nominate one significant other, who is their closest confidant and who is over 18 years of age. If participants live alone, their significant other should be someone that they see at least once a week. Consent will be sought from significant others to take part in the study. If a significant other does not meet eligibility criteria or does not give consent to take part, we will approach up to three alternative individuals nominated by the person with aphasia. People with aphasia without a significant other or whose significant other does not consent to the project will still be eligible to take part.

#### Peer befrienders

Peer befrienders will be people with mild-moderate aphasia who are over 18 years of age, are at least 1 year post-stroke and meet the selection criteria identified by a group of consultants with aphasia during the development phase of this project (see User Involvement). These comprise good adjustment post-stroke; be open, confident, resilient and willing to talk with others; able to concentrate for up to 3–4 h (to travel to and complete visits); and able to use public transport or to drive. The Trial Manager who will check eligibility and give initial information about the project to potential peer befrienders is a highly specialist SLT and will informally assess befrienders’ aphasia. The Trial Manager will also administer the short Frenchay Aphasia Screening Test to ensure they score a minimum 5/10 for auditory comprehension and 5/10 for verbal expression.

#### Exclusion criteria

Participants with aphasia, significant others and befrienders will be excluded from the study if they have the following:

- Other diagnoses affecting cognition or mental health, such as, but not restricted to, advanced Parkinson’s disease, motor neurone disease, dementia, clinical depression. This will be based on medical records for participants with aphasia and self-report for significant others and peer-befrienders, as well as the General Health Questionnaire 12 (GHQ-12) [32] as a depression screen for peer-befrienders who will be excluded if they score ≥ 3.
- Severe uncorrected visual or hearing problems, based on medical records for participants with aphasia and self-report for significant others and peer-befrienders.
- Severe or potentially terminal co-morbidities, on grounds of frailty, based on medical records for participants with aphasia and self-report for significant others and peer-befrienders.

People with aphasia will also be excluded if they are discharged to a geographical location away from the borough of the recruiting hospital, as this will make it unfeasible for peer-befrienders to visit those in the intervention arm.

### Recruitment

For those identified in hospital, people with aphasia and significant others will be screened for eligibility by the CRN Nurse or the hospital SLT, who will also seek consent for inclusion into the study. The CRN nurse and the hospital SLT are members of the clinical team and will know if a patient is eligible /have access to patients’ medical records to assess eligibility. Potential participants will be approached, given information about the study, and recruited to the study before the person with aphasia is discharged from hospital and close to the time of discharge. For participants who are discharged from hospital prior to consent being obtained and for those identified in the community, a community CRN nurse, community SLT, or a member of the research team will screen them and obtain consent from those willing to take part.

For those recruited while still in the hospital, there is a gap between recruitment to the study and baseline assessment, often 6–8 weeks long while they receive intensive input from an early supported discharge (ESD) team in their community. Such teams provide intensive (i.e. 3 or more sessions weekly) input from a dedicated multi-disciplinary team. When a person expresses interest in the study while in hospital but does not meet specific eligibility criteria that may change after discharge, i.e. borderline fail (score of 3 on DISCS) on emotional distress as indicated above, visual or hearing problems that may be corrected, or temporarily discharged to a borough away from the recruiting hospital, they will be asked for their consent to be re-approached for a second screen in the community. This will ensure we do not miss participants who do not meet eligibility criteria when approached in hospital but do meet these criteria after discharge. The second screen will be organised within the first 2 weeks of the person’s return to the community or as soon as possible after that if this is not feasible.

Training for interacting with people with aphasia will be provided by the Trial Manager who is a highly specialist SLT to the CRNs completing consent. The numbers of people screened, identified as eligible, and consented will be recorded at each site and provided to the Trial Manager at the end of each month. Potential participants identified in hospital but discharged before screening will have their details forwarded to the Trial Manager with their consent and will be screened and consented into the study by a member of the research team.

Significant others will be recruited at the same time as people with aphasia. However, in cases where this is not possible, recruitment will occur during the initial home visit.

Nominations for potential peer befrienders will be received from recruitment sites (e.g. community SLT, hospital), local services, and voluntary organisations (e.g. The Stroke Association, Re-Connect). Peer befrienders will be screened for eligibility and consented into the study by the Trial Manager.

### Ethical issues

The trial will be conducted in compliance with the principles of the Declaration of Helsinki (1996), the principles of Good Clinical Practice (GCP), and in accordance with all applicable regulatory requirements including but not limited to the Research Governance Framework and the Mental Capacity Act 2005. Ethical approval to conduct the study was granted by the NHS Health Research Authority London-Bloomsbury Research Ethics Committee (ref 16/LO/2187). Local NHS Research and Development approvals were also gained from all participating sites.

#### Informed consent

Informed consent will be obtained from all participants. The person obtaining consent will have experience of or receive training on communicating with people with aphasia using a total communication approach (e.g. explaining in simple short sentences, using gestures, pointing to pictures) and obtaining informed consent. To ensure that each participant fully understands the nature of the study, participant information materials and consent forms were developed that are accessible to people with aphasia. The materials were developed following standard aphasia-friendly principles, such as presenting one idea at a time, using short simple sentences presented in large font, emboldening keywords, and representing key ideas with a suitable pictorial image. During the development phase, we used templates created by the NIHR CRN for enabling people with aphasia to participate in research [33] and principles from the Consent Support Tool, which has been specifically designed to facilitate the consent process with people who have aphasia [34]. Each participant will have time (up to 48 h) after information is provided to make an informed decision about whether they would like to consent to inclusion in the study. Any questions or queries they may have about the study will be discussed with the person obtaining consent.

For participants with aphasia, the person obtaining consent will ask them three simple yes/no or forced alternative questions after information giving, to check their understanding of key aspects of the study: ‘Is this study about a drug or how you feel?’, ‘Will our researchers visit you once or many times?’, ‘Can you stop if you wish, yes or no?’. If participants with aphasia cannot answer these correctly, this will suggest they have such severe aphasia that they are unable to give informed consent and they will not be included in the study. For participants who are physically unable to sign the form (e.g. due to weakness in dominant hand due to stroke), then consent will be given using a mark or line in the presence of an independent witness (who has no involvement in the trial) who will then corroborate by signing the consent form.

#### Two-stage consent process

In behavioural interventions, blinding participants to treatment versus control allocation is problematic. If participants are provided with information about the intervention to be tested, as ethics guidelines require, they will know whether they are in the intervention or the control arm of the study. This is particularly problematic in psychological interventions where people who may already be distressed or anxious are likely to become even more distressed when they realise they are in the control arm of a study. Where a participant is aware that they have been allocated to the control condition, there are potential threats to validity and maintaining lack of bias [35, 36]. These threats include the ‘resentful demoralisation’ effect [37] whereby when participants are allocated to their non-preferred arm of a trial, it leads to deflated scores on psychological outcome measures and/or non-compliance; selective differential attrition rates between groups; and lack of consent to randomisation from potential participants with strong preferences over group allocation [35, 36]. Indeed, in our team’s previous experience with a peer support intervention with mental health patients at the time of discharge, many potential participants refused to take part unless they were guaranteed to receive the peer support [38]. In such circumstances, some advocate the use of a Zelen design where only those in the experimental group consent to the trial. An ethical concern with such a design is that participants are included in a study without their consent.

To minimise these threats, we are following a modified two-stage consent design [39, 40], as described in the Medical Research Council (MRC) framework for complex interventions [41]. This will hopefully encourage recruitment to and retention in the trial while maintaining participants’ blinding to group allocation. First, we will invite participants to join a study monitoring progression and adjustment to life post stroke and aphasia and comparing different packages of care. Those who consent will be randomised. Those assigned to the control group will receive usual care; they will know that other people may receive different care, against which they will be compared, but they will not know what this entails. The second stage of consent will involve only those participants who have been randomised to peer befriending. A separate visit (conducted within their own home/community setting) will be conducted by the Trial Manager within 1–2 weeks of randomisation to inform participants of their allocation in the intervention arm of the trial, give them information about the peer befriending, and get their consent to participate in this arm. No instances are expected to occur where a participant in the control arm may need to be unblinded.

### Randomisation

Participants will be randomised within three (3) days of the baseline assessments being completed. For participants who receive ESD therapies, baseline assessments will occur once this input is completed. For the rest, baseline assessments will occur within 1–2 weeks from discharge home.

King’s Clinical Trials Unit (KCTU) will provide the randomisation service via their web-based service held on a secure server in accordance with KCTU standard operating procedures. On randomisation, a unique patient identification number (PIN) will be assigned. Each participant will be randomised 1:1 to usual care + peer befriending (PEER) or usual care (USUAL). The randomisation allocation will utilise minimisation to promote balance on important prognostic characteristics, with a random component to avoid predictability of allocation. Minimisation will be based on the following characteristics: severity of aphasia (based on WAB cut-offs, 3 levels), recruitment area (3 levels), and physical ability (wheelchair user or not).

**Fig. 1:**
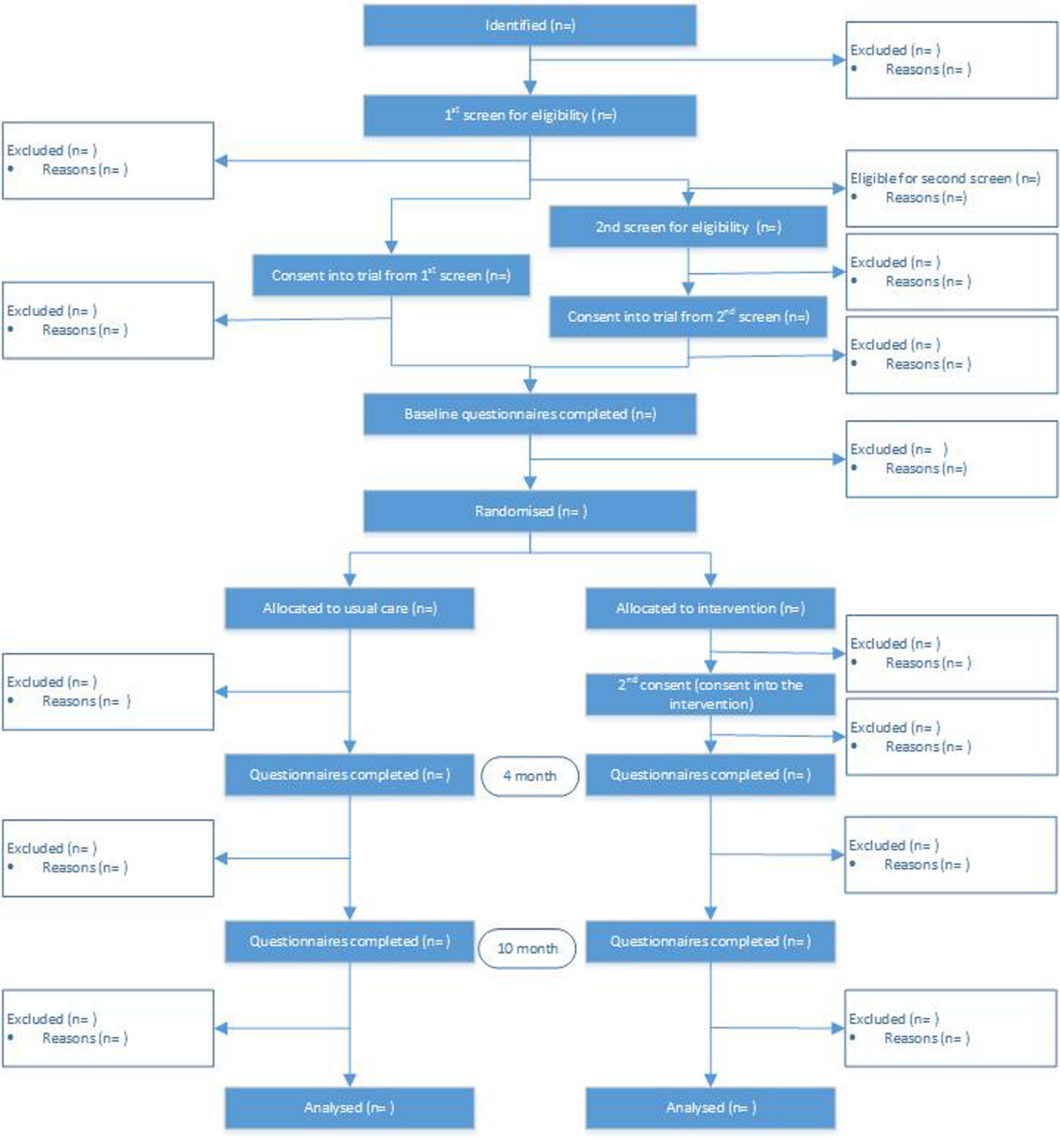
CONSORT diagram of recruitment and participation for people with aphasia

**Fig. 2:**
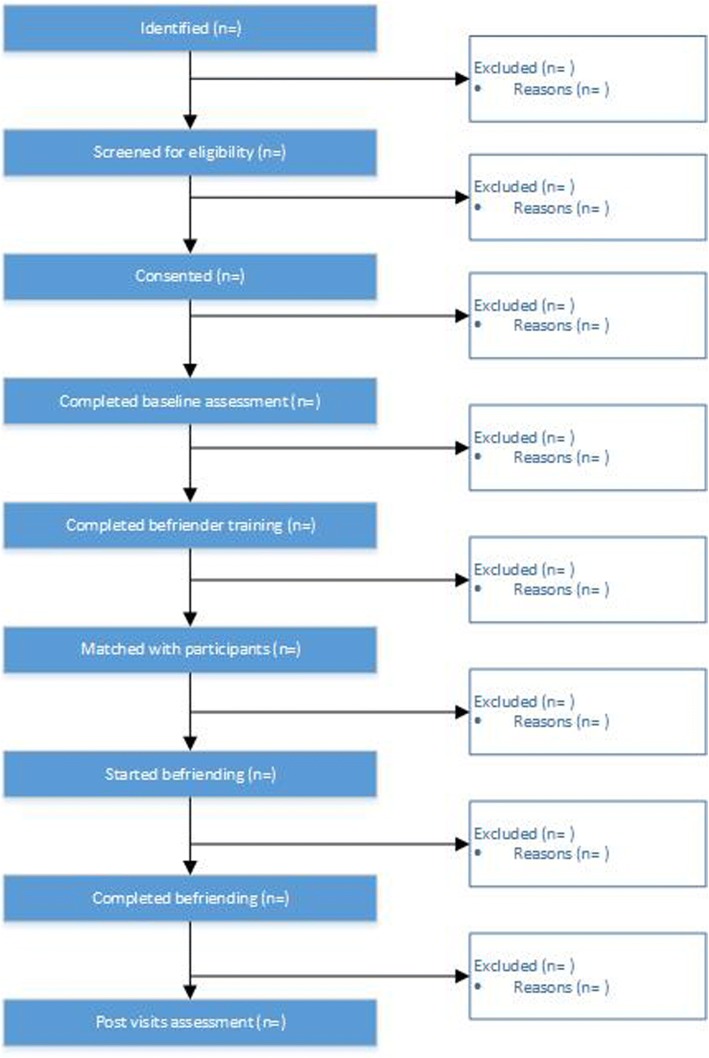
CONSORT diagram of recruitment and participation for peer befrienders

A CONSORT diagram of recruitment and participation in the study is shown for people with aphasia (Fig. 1) and peer befrienders (Fig. 2).

### Blinding

The chief investigator, study statisticians, and research assistants collecting post-baseline quantitative data will be blinded to treatment allocation until data collection is complete. To ensure blinding of quantitative outcome measures, research assistants will not have access to participant details, they will follow a script when completing visits, all visits will be arranged by the Trial Manager, and participants will be asked to not reveal information regarding interventions they have received during assessment. If a research assistant becomes unblinded, they will report this to the Trial Manager who will keep a record of such instances. If this happens during the 4-month assessment point, the 10-month assessments will be completed by another research assistant. Statisticians will receive treatment group with partially blind coding (i.e. A and B), and data that would unblind will be stored in a separate database that will not be accessed by the trial statisticians until the main primary analysis is done. The Trial Manager, qualitative researchers, health economics researchers, and the befriender supervisor will be unblinded.

### Intervention

#### Intervention arm—usual care + peer befriending (PEER)

Participants in the intervention arm will receive all usual care available in their borough and peer befriending. Peer befriending aims to utilise the skills, knowledge, and ‘lived experience’ of people with longer-term aphasia to offer emotional, social, and informational support to others with aphasia. It aims to help people move forward and develop their own strategies for adjusting to life post-stroke. In the trial, peer befriending is offered at a time of transition (discharge from hospital and withdrawal of intensive therapeutic input) and increased need. The intervention is described according to the TIDieR checklist [28] (see Additional files 2).

Peer-befrienders will receive training based on a study-adapted version of a peer befriending intervention manual [42] and ongoing supervision and support. The training will last 5–6 h across 2–3 days to accommodate the needs of the participants in the group (e.g. levels of attention and fatigue). The training will be conducted by two facilitators, who both have experience of communicating with people with aphasia. One of the trainers has additional experience of running peer befriending schemes. The training will cover a range of topics related to peer befriending (e.g. the role of a befriender, hopes and fears, how to have a conversation as a befriender, setting objectives, identifying barriers, dealing with challenging situations), health and safety, and dealing with adverse events. Peer befrienders will receive a copy of a peer befriender handbook, which contains key information from the manual in aphasia-accessible format. It is anticipated that each peer-befriender will work with 2–4 participants during the trial and no more than two at any one time.

Peer-befrienders will be offered monthly group supervision sessions. These sessions will be conducted by a single befriender facilitator (also involved in the training) who is a trained SLT with extensive experience in working with people with aphasia. The sessions will be an opportunity to share experiences and discuss any difficulties that have arisen. Peer-befrienders who are facing particular challenges will receive supplementary individual supervision and support from the same befriender facilitator. Peer befrienders and the facilitator will record these challenges. The facilitator will keep detailed written notes to summarise the content of each supervision session.

Trained peer-befrienders will visit participants in their own home six times (each visit aimed to be at least 1 h) over a period of 3 months. A further two visits within the next 6 months will also be offered for a gradual transition to the end of the peer befriending. Participants will be paired with and introduced to their peer-befriender as soon as possible after and within 1 month of randomisation. If this is not feasible, the reasons for this will be recorded. Where possible, pairing will take account of preferences around gender, cultural factors, age, and personal interests. The schedule and nature of visits will be agreed between the pair at their first meeting. This meeting will also identify possible goals for the intervention. For example, participants might highlight concerns that they would like to discuss or activities that they would like to pursue. Subsequent visits may include conversation, problem-solving, trips out e.g. to a local group, and joint activities.

After each visit peer-befrienders will complete an aphasia-friendly record sheet. This will include whether a visit was cancelled and reason why, length of visit, topics discussed, activities undertaken, any decisions made, and date and time of next visit. Peer befrienders will complete this record sheet immediately or as soon as possible after each visit if necessary with the help of the befriender facilitator who will collate these sheets during monthly supervision sessions. The facilitator will pass the record sheets on to the Trial Manager on a monthly basis.

#### Control arm—usual care (USUAL)

The control group will receive usual care, comprising of all the health care, social care, and voluntary services available in their borough. It is not known what exactly usual care comprises for people with aphasia who are discharged in the community with low levels of psychological problems. This trial will help to document usual care by administration of the CSRI questionnaire.

#### Intervention fidelity

The fidelity of the peer befriender training and supervision and the peer-befriending intervention will be evaluated. Fidelity checklists have been created for each of these three session types to measure how closely they adhere to the peer befriending intervention manual, the handbook, and the intended aims of the intervention. These checklists have been informed by the Health Behaviour Change Competency Framework (HBCC) [43] and rating scales of the interactions of people with aphasia and their communication partners [44].

All training and supervision sessions will be videotaped. All training sessions and all supervision sessions, except the first and the last, will be watched by unblinded team researchers and research students and rated against the fidelity checklists to ensure training and supervision are delivered as intended. With participants’ consent, a proportion of befriending visits (1 per participant) will also be videotaped and watched against a fidelity checklist. Results from these checks will be reported back to the befriender facilitator to inform later content of the supervision sessions.

Information from the supervision notes and from the peer befriender visit record forms will also be used to evaluate whether peer befrienders followed the peer befriending handbook and to compare content of intervention between and within different peer befrienders. This information will provide additional qualitative information and context on the fidelity of the intervention.

### Procedures

Outcome measures data will be collected by blinded research assistants/assessors who are trained and skilled in communicating with people with aphasia. Assessors will be given a data collection pack for each participant, at each time point. These packs will contain a script to guide their conversations with participants and verbal instructions for each of the outcomes administered. During the development phase of the trial, an example assessment session was videotaped between a member of the research team and a person with aphasia (consultant with aphasia, see User and public involvement). This training video will be used to ensure consistent administration of the outcome measures. In addition, the Trial Manager will observe the first videotaped assessment session of each assessor. Further assessment sessions will be observed at random by the Trial Manager either in person or by reviewing videotaped sessions.

The qualitative research assistant conducting the semi-structured interviews will similarly be supported. They will receive training in conducting semi-structured interviews from a senior qualitative researcher in the team, who has extensive experience of adapting qualitative methodologies for people with aphasia. The senior qualitative researcher will listen to two audiotaped initial interviews and give feedback, for example, to ensure questioning is unbiased and leads to the full exploration of topics. They will continue to support the qualitative research assistant throughout the trial and will periodically listen to interviews.

For the economic evaluation, either the Trial Manager or the qualitative research assistant will collect information using a stroke adapted version of the Client Service Receipt Inventory (CSRI) [45]. They will be given training in completion of the CSRI and eliciting resource use information by a member of the study team with relevant health economics expertise. As much information as possible will be collected from the significant other as primary respondent, where available. The Trial Manager will draw additional information by contacting CRN nurses or members of the clinical team as appropriate.

To facilitate participants to attend baseline and follow-up sessions, participants, significant others and peer befrienders will be contacted by text/telephone at least 1 week prior to each assessment session and on the previous day to confirm appointment times. To maximise retention and participant engagement in the project, a quarterly newsletter will be sent out, and a single phone-call between follow-up assessment times will also be made to remind them of their next appointment.

### Measures

At baseline, all participants will complete a case history covering: demographic and health information, family and social circumstances, and personal interests. This information will be used to report on participant characteristics and will also contribute to pairing with peer befrienders for those in the intervention arm. For participants with aphasia, aphasia will be fully assessed with the Western Aphasia Battery-Revised [46], and cognition with the Cognitive-Linguistic Quick Test (CLQT) [47], which has been specifically developed for people with aphasia. These assessments will be reported descriptively under participant characteristics.

#### Feasibility outcomes

Feasibility outcomes will include the following:

- Proportion of participants with aphasia who are eligible of those screened
- Proportion eligible at first screen
- Proportion eligible at second screen
- The rate of eligibility per month
- Proportion who consent of those eligible
- The rate of consent per month
- The rate of recruitment (participants randomised) per month
- The frequency and proportion of people consented who withdraw overall, by study arm, and by those who do before and after randomisation. This will specifically include describing those in the PEER arm who decline consent at the second stage
- Acceptability of research procedures and outcome measures based on qualitative interviews
- Acceptability of the intervention and also usual care to participants, their significant others, and peer befrienders, based on qualitative interviews
- Documentation of usual care, based on data from CSRI
- Fidelity and adherence, based on session observations, visit record sheets, and supervision records.

Moreover, we will informally ask CRN nurses and other members of the clinical teams involved with SUPERB in all our sites about their experiences with the trial. This information will provide additional context to our feasibility outcomes.

#### Patient-reported outcome measures

A range of outcome measures will be used with participants with aphasia covering mood, wellbeing, activities, and communication and social participation. Measures chosen have either been developed specifically for neurologically impaired populations, some including people with aphasia or have been previously used with people with aphasia with good evidence of accessibility and acceptability. Where appropriate, the presentation of measures will be modified to make them aphasia-friendly in line with best practice guidelines [48]. The content, however, will not be changed to avoid affecting measures’ psychometric properties.

The following primary outcomes for participants with aphasia will be completed at baseline, 4 and 10 months post-randomisation: General Health Questionnaire-12 (GHQ-12) [32], using 0 0 1 1 scoring and the Depression Intensity Scale Circles (DISCS) [30]. The DISCS is being measured because some participants may not be able to complete the GHQ-12. This feasibility study will assess how often this is the case. The DISCS will be treated as the primary outcome measure only if there is ≥ 10% missing data in the GHQ-12 due to severity of aphasia; otherwise, DISCS will be a secondary outcome measure.

The following secondary outcomes for participants with aphasia will be taken at the same time points: Short Warwick Edinburgh Mental Well-being Scale-7 [49], Communicative Participation Item Bank [50], Community Integration Questionnaire—Adapted [51]. Proportion with high emotional distress vs low emotional distress as measured using the GHQ-12 (high distress = score of 3 or more, low distress = score of 0–2). There are additional psychological constructs that may be affected by peer befriending; however, the evidence base is not strong on whether existing measures on these constructs are responsive to change when used with people with aphasia. We will include two exploratory self-report outcomes for participants with aphasia in this study, the Communication Confidence Rating Scale for people with aphasia [52, 53] and the Friendship Scale [54] in order to see whether they may be suitable to use as secondary outcome measures in a definitive trial. These will be completed by participants with aphasia at each of the three time points.

The following outcomes for significant others will be taken at baseline, 4 and 10 months post-randomisation: Warwick Edinburgh Mental Well-being Scale [55]; General Health Questionnaire-28 (GHQ-28) [32], with 0 0 1 1 scoring and descriptive information on its four subscale scores; and Bakas Caregiving Outcome Scale [56].

Outcomes for peer befrienders will be taken at baseline and on completion of two befriending cycles, i.e. six befriending visits with each of two participants. For befrienders who do not fully complete a cycle because, e.g. the participant with aphasia dies or withdraws, the cycle will be considered completed if they have done a minimum of two visits. Outcomes comprise the Warwick Edinburgh Mental Well-being Scale-7; Community Integration Questionnaire—Adapted; and General Self-Efficacy Scale [57].

#### Qualitative outcomes

Interviews will be conducted with ten participants with aphasia in each arm of the trial at 4 months post-randomisation. These interviews will explore participants’ experience of taking part in the study and also what has helped participants in adjusting to living with stroke and aphasia. They will probe what participants have found useful in terms of emotional well-being, confidence, and thoughts about the future. The interviews will also explore participant experiences of services they have received in terms of their psychological well-being post stroke. Those who received peer befriending will also be asked about their experiences of the intervention, its perceived benefits, and any difficulties. The subset of participants interviewed from the PEER arm of the trial will be re-interviewed at 10 months about the longer term impact of the stroke and aphasia and their perceptions about the intervention.

A sub-sample of five significant others from each arm will be interviewed at 4 months to explore the impact of the stroke and aphasia on their life and family life and their perspectives of the care received. Those in the PEER arm will also be asked about the administration and impact of peer befriending. All interviewed participants will be selected purposively to capture a diversity of views. Key sampling criteria for participants with aphasia are severity of aphasia and whether the person lives alone. Secondary criteria are geographical area, gender, mobility, GHQ-12 score, and ethnicity. For significant others, sampling criteria include relationship to person with aphasia (partner/spouse or child/other), ethnicity, gender, and GHQ-28 scores.

All befrienders will be interviewed after they have completed two cycles of befriending (minimum two visits per cycle) to explore their experience of the study, including reflections on programme training and delivery, support received, any concerns or difficulties, and perceived benefits of peer befriending.

#### Economic evaluation

The European Quality of Life measures, 5 dimensions, 5 levels (EQ-5D-5L) [58] will be collected for participants with aphasia at baseline, 4 and 10 months post-randomisation, and the Stroke-adapted CSRI at 4 and 10 months post-randomisation.

### Sample size

We will recruit in total 60 participants with aphasia (30 in each arm of the study). Allowing for a ~ 15% lost to follow-up rate, at least 50 will complete the study. With 60 participants recruited, we will be able to estimate a 95% confidence interval for the recruitment rate to within approximately 25%. This sample size will be adequate to estimate important parameters needed to inform the design and the sample size of a full trial, such as the standard deviation, consent rates, event rates. This sample size also meets recommended sample sizes for feasibility studies [59].

Although this is a feasibility study, we would like to estimate exploratory differences between the PEER and USUAL arms. If we assume we retain 50 participants at follow-up, a two-sided test, *α* = 0.05, and independence of participants, we will have 80% power to detect an effect size of approximately 0.8. The standard deviation of the primary GHQ-12 outcome in a generic population of patients with stroke has been shown to be approximately 3.6—this was using the 0 0 1 1 scoring, with total scores ranging from 0 to 12 [60]. This effect size corresponds to an approximate mean difference of 3 points between groups. This sample size is sufficient for these exploratory comparisons; however, in a definitive trial, we would want to have higher power to detect a smaller effect size. This feasibility study will give us a standard deviation specific to the group that we would be interested in recruiting for a definitive trial, so would help to inform such a sample size calculation.

### Statistical methods: data analysis

#### Feasibility outcomes

As SUPERB is a feasibility trial, the feasibility outcomes are the most important/will take precedence. We will calculate proportion who are eligible of those screened (number eligible/number screened), proportion who are eligible at first screen (number eligible/number screened at first screen) and second screen (number eligible/number screened at second screen), rate of eligibility per month, proportion who consent of those eligible (number who consent/number eligible), rate of consent per month, and rate of recruitment (participants randomised) per month. The frequency and proportion of people consented who withdraw overall, by study arm, and by those who do before and after randomisation will be presented. This will specifically include describing those in the PEER arm who decline consent at the second stage. Appropriate 95% confidence intervals will be constructed for all of the above measures.

#### Descriptive statistics

Trial flow data will be reported as outlined by the CONSORT statement. We will use descriptive statistics to document what usual care consists of, based on data from the CSRI. The primary and secondary outcomes will be summarised using summary statistics, for the entire trial population and by trial arm, at each trial time point. The primary and main secondary outcome means and confidence intervals will be plotted over time. These summary statistics will help inform sample size estimation for the definitive trial.

#### Outcome measures’ analyses

Despite the feasibility nature of the trial, we will compare the PEER and USUAL groups. These will be considered strictly exploratory. For primary outcome analysis, the comparison will be between the PEER and USUAL groups. The GHQ-12 will be scored 0 0 1 1 and summed, resulting in a 0–12 score range. This overall total GHQ-12 score will be analysed using linear mixed models with the 4 and 10 months post-randomisation measures as dependent variables, with a random intercept for individuals, and time, the baseline GHQ-12 score, a dummy variable indicating treatment group, the minimisation stratification factors, and any baseline variables that were imbalanced or that predicted missingness as independent variables to improve the plausibility of the missing at random assumption. A treatment group by time interaction term will be included to allow for extracting comparisons at the 4- and 10-month post-randomisation time points. These models will account for missing data using the maximum likelihood algorithm.

In addition, if at least some peer befrienders see multiple participants (e.g. > 2), we will explore the possibility of calculating the GHQ-12 intra-class correlation coefficient (ICC) by fitting a linear mixed effects model using the data from the PEER group with the 4 and 10 months post-randomisation measures as dependent variables and a random intercept for befriender to calculate the within-peer, between-peer, and total variability for clusters of participants seen by the same peer befriender. This ICC will help inform a sample size calculation for a larger trial and provide estimates of a peer befriender ICC to publish in the literature. If the DISCS is used as the main primary outcome instead of GHQ-12, it will be analysed in a similar manner.

The significant other outcomes and the secondary and exploratory participant with aphasia outcomes will be analysed in a similar manner to the primary outcome, comparing the outcomes between the PEER and USUAL groups. The peer befriender outcomes will be compared between the baseline and post-befriending time points using a paired t-test.

All analyses will initially be conducted on an intention-to-treat basis. A second per protocol analysis will be done on the primary and secondary outcomes, excluding participants who are not in compliance with the protocol. Participants deemed not compliant will include those who did not consent at the second stage, completed less than six peer befriending sessions, were found to be ineligible after randomisation based on the inclusion/exclusion criteria, and participants in the usual care group who received peer befriending outside the study as reported to assessors or measured using the CSRI (in addition to their removal, we may explore analysing them in the peer group).

#### Missing data

The amount and reasons for missing data will be summarised overall and by treatment group. Missing data will be accounted for where mixed models are applied under the missing at random assumption using the maximum likelihood algorithm. The baseline characteristics of those missing follow-up and those with complete follow-up will be summarised and variables affecting missingness will be examined using a logistic predictor of missingness model. This will be done by generating a binary variable for missingness for the primary outcome variable 10 months post-randomisation (we will explore the need to do this either separately or combined for the primary and secondary outcomes) and regressing this on baseline variables. Any variables found to be important predictors of missingness will be included in the primary and secondary outcome models. From a health economic perspective, where there is missing data, we will analyse the data on a complete case analysis basis and additionally use mean imputation for missing values. This will allow for a sensitivity analysis indicating whether missing values could be biasing the data.

#### Qualitative analysis

All qualitative data will be analysed using Framework Analysis [61]. Initial themes and concepts will be identified through reviewing the data. These will then be used to construct a thematic index, used to assign a label to each phrase or passage. The labelled raw data will then be summarised and synthesised into the thematic charts. This matrix-based method of analysis will facilitate systematic exploration of the range of views including both between cases and within cases in order to produce both descriptive and explanatory accounts of the data. To minimise potential bias, a second analyst will independently index a proportion of transcripts and analyse the matrix-based material.

#### Economic evaluation analysis

This pilot economic evaluation study has the objective of detecting problems in the cost collection, exploring how patients respond to the primary outcome in the study, the GHQ-12, and comparing it to the EQ-5D-5 L instrument, as well as developing the economic evaluation model that will be applied to a phase III RCT. The pilot economic evaluation will compare incremental costs to incremental health gains of the PEER arm versus USUAL arm. Due to the very low numbers, this will be strictly an exercise to detect problems in the process of data collection and development of the full economic evaluation model. The rationale is to use the feasibility stage data to prepare a future full trial cost-effectiveness.

For the costs, we will use data collected on the CSRI on service use (including health, social, and voluntary services) and associated costs at 4 and 10 months post-randomisation. Unit costs of resources used will be derived from routine sources locally where possible and from national sources such as the NHS reference costs.

Health gains will be obtained from the answers to both the GHQ-12 and to the EQ-5D-5 L instruments. The gold standard for economic evaluation is to use generic health state outcome measurements because these allow comparability across clinical areas. Thus, we will run two types of pilot economic evaluation analyses. First, we will run a pilot cost-effectiveness analysis based on the GHQ-12. Second, we will perform a pilot cost-utility analysis using quality-adjusted life years (QALYs) gained based on the answers to the EQ-5D-5 L. Additionally, since there is limited evidence on how the GHQ-12 and EQ-5D-5 L relate to each other [38], we will use the pilot data to explore the correlation of these two instruments in terms of health gains in different domains.

With the pilot cost and outcome data, we will explore the calculation of confidence intervals for costs and health gains using non-parametric bootstrapping, with the understanding that low numbers preclude generalisability. Also, we will explore the application of probabilistic sensitivity analysis that will eventually generate cost-effectiveness acceptability curves in a Phase III RCT. These curves graphically represent provisional estimates of the cost-effectiveness of the intervention.

#### Fidelity

Videotaped sessions will be rated against fidelity checklists. Fidelity of the peer befriending training and supervision to the training manual and the peer befriending intervention to the peer befriending handbook will be evaluated by calculating a per cent fidelity score (components implemented/components planned × 100). Inter-rater (two raters) and intra-rater reliability will be calculated for all three session types. Inter-rater agreement coefficients will be used to calculate inter and intra-rater reliability. Counts of sessions completed will indicate whether befriender/befriendee pairs have adhered to the six befriending visits.

### Data management

The main trial database will be hosted at King’s Clinical Trials Unit (KCTU). Data will be managed using the InferMed MACRO database system. This system is regulatory compliant (GCP, 21CRF11, EC Clinical Trial Directive). Data will be entered by authorised staff (Trial Manager and research assistants) with a full audit trail in which participants will be identified by their unique PIN. The database will include feasibility data on people consented, with these data being aggregated where we do not have consent to hold participants’ data (for example, reasons for declining participation), as well as assessment and outcome measures data, and serious adverse event data.

A separate database storing intervention details will also be hosted at KCTU. This will include all necessary information on the intervention each participant in the PEER arm received as extracted by visit record forms and information from supervision sessions (e.g. number of sessions arranged, completed, missed and reason why; duration of session; topics discussed). The intervention database will meet the same requirements as the main trial database above, but only the Trial Manager will be authorised to input data and have access. Trial statisticians will only have access after unblinding. Data in the second database will be extracted and summarised routinely by an unblinded statistician independent to the trial when necessary.

Fidelity data, qualitative data, and health economic data will be kept at City, University of London, on a secure network drive within the City system, which is regularly backed up. The fidelity dataset will be anonymised with participants being identified by their PIN. The Trial Manager and unblinded researchers analysing fidelity data will have access to it. Qualitative data will be transcribed and then saved by the qualitative research assistant in an electronic database. Participants in the qualitative data database will be identified by their unique PIN; however, this database will also include identifying information, such as gender and age. Health economic data will be entered onto a password-protected Excel spreadsheet. Each participant on this spreadsheet will be identified by their PIN. Qualitative and health economics data will be accessible by the Trial Manager and qualitative and health economics researchers.

### Site monitoring

The Trial Manager will conduct on-site/central monitoring. The Trial Manager will do monthly checks on data completeness and range checks as outlined in the data management plan. In addition, regular checks of a selected number of participant records will be done at three monthly intervals to check that data recording procedures are being followed consistently and accurately. The data will also be perused by the trial statistician when extracted for Data Management Committee reports and at the end of the study. Data queries will be raised by the trial statistician and sent to the Trial Manager for resolution. Any issues found in any of these checks will be resolved against the original source records and corrected in the MACRO database where possible.

### Anticipated risks and benefits

The risk of harm is considered unlikely to participants and low risk, which was further confirmed by early user consultation. Research assistants and peer befrienders visiting participants with aphasia will have a Disclosure and Barring Service (DBS) check, to ensure participant safety. There are no formal statistical criteria for stopping the trial early. Decisions to stop the trial early on grounds of safety or futility (with regard to recruitment) will be made by the Trial Steering Committee on the basis of advice from the Data Management Committee.

Some participants may experience fatigue and poor concentration during assessment sessions. To minimise this, information will be provided in an accessible aphasia-friendly way and researchers will offer breaks as and when needed. It will also be made clear to participants that they can request a break at any time or additional visits to complete outcomes.

There is the risk that participants may become burdened with their involvement in the study. Participants may get upset during assessment and/or outcome measurement, or peer befriending visits. All researchers and peer befrienders will be trained and supervised to deal with these situations, for example they may take a break, see if the participant wants to talk about their feelings, or stop the session if needed. Issues raised by peer befrienders will be discussed in monthly supervision with the befriender facilitator, with additional individual support provided as needed. If a participant or peer befriender scores within the high emotional distress range of the GHQ-12 (3 or more) or the GHQ-28 for significant others (6 or more), the research assistant or Trial Manager will discuss with them their score and ask them to consider talking to their GP. If they would like, they will be given information on local support organisations and groups. For peer befrienders who score within the high emotional distress range of the GHQ-12, the Trial Manager will explore with them whether they find peer befriending distressing and whether they need to be withdrawn from the study.

In terms of benefits, participants in this study will receive all their usual care; no treatment will be withdrawn due to participation in the study. Information about local support services and voluntary organisations that support well-being post-stroke will be offered to those experiencing low mood and it will also be offered if participants express feelings of loneliness and social isolation. Trained befrienders will also be linked to local support organisations, where their skills can be used in the future to support people with aphasia.

### User and public involvement

People with aphasia are engaged at every stage of the SUPERB trial, and the trial cannot run without their involvement. While developing the proposal for this study, we held a consultation event with six people with aphasia, where they reviewed and influenced our plans for the study. Prior to the commencement of recruitment, we had a 6-month development phase, where six consultants with aphasia with extensive experience in delivering peer befriending advised on key decisions through a series of six 3-h workshops. These decisions included: the criteria for a peer befriender, choice of outcome measures, design of information sheets and consent forms, content of peer befriending training manual and handbook, and questions to ask during qualitative interviews. During the trial, we will train and employ people with aphasia as peer befrienders, i.e. they will deliver the intervention tested in this study. In our evaluation, apart from feasibility and patient-reported outcomes, we will elicit the views of participants with aphasia, their significant others, and peer befrienders on the intervention and study processes in qualitative interviews. Lastly, a user group comprising at least four people with aphasia and one significant other will advise on the study. The user group will meet five times during the course of the trial and will advise on management issues, the implications of the findings, and dissemination to the stroke community.

### Trial management

Three committees have been established to govern the conduct of the study: the Trial Management Committee, the Trial Steering Committee (TSC), and the Data Management Committee (DMC). The Trial Management Committee will comprise the Chief Investigator and Co-investigators, the Trial Manager, and junior statistician and will meet monthly to manage the project. The Trial Management Committee will sign off the protocol and agree to all standard operating procedures before the start of recruitment. The committee will also provide overall supervision of the trial including trial progress, adherence to protocol, patient safety, and consideration of new information. The committee forms a strong multidisciplinary team who will report to the TSC.

The TSC will consist of an independent chair and independent and non-independent members, as well as the Trial Manager and key partners from our recruiting sites. This group will meet six times during the project to oversee the study management. They will also be consulted via email as and when needed. They will be responsible for reviewing and approving trial documentation (e.g. protocol, information sheets, and consent forms) and oversee the conduct of the study, including advising on continuing or stopping the study in light of advice from the DMC. This committee will advise on processes (e.g. fidelity checking; monitoring and reporting adverse events) and issues (e.g. recruitment to target; site-specific issues) as they arise.

The DMC will consist of an independent from the study chair; an independent specialist with interest in trials for people with stroke and aphasia; and an independent statistician. This committee will meet four times to approve the data management plan, monitor adverse events, and monitor the progress of the trial in relation to safety and ethical issues, including recruitment, uptake of the intervention, withdrawal from the trial, descriptive summaries of the outcomes and any other variables they feel are critical to trial monitoring. They will meet again on completion of data collection. The DMC and the trial statistician will discuss the level of access to unblinded data and will include this level of access and any procedures for unblinding the DMC in the DAMOCLES charter that will be agreed at the first meeting. The DMC will report to the TSC and may advise the TSC to continue or to stop the trial should they feel this is necessary based on their monitoring of the data.

## Discussion

This exploratory (phase II) RCT will evaluate the feasibility of a phase III RCT on the clinical and cost-effectiveness of peer befriending for people with aphasia post-stroke. Effect sizes with 95% confidence intervals will be estimated to check that the likely effect is within a clinically relevant range as confirmation that it is worth planning a definitive trial. This information together with acceptability of the study data, safety of intervention, participant recruitment, and retention rates will help us determine whether the definitive RCT is feasible and whether modifications are needed to the intervention and/or the protocol. As multiple outcomes are considered in this study, including qualitative data, we have not set specific targets for the feasibility outcomes. According to the CONSORT 2010 extension statement for pilot and feasibility trials, even when such targets are set, they may be best viewed as guidelines rather than thresholds for progression [62]. As recommended in the CONSORT 2010 extension statement, we will consult with key stakeholders, including our TSC and user advisory group, when considering whether to progress to a definitive trial and potential amendments to the protocol. We will draw on the ADePT model (A process for Decision-making after Pilot and feasibility Trials) [63] to explore the best pathway forward from this feasibility study. The ADePT model provides a framework which enables (1) systematic identification and appraisal of problems and potential solutions/amendments, (2) increased transparency in the decision-making process, and (3) a process to make clear any tensions which may exist between explanatory and pragmatic choices (i.e. potential solutions which may work well within the trial, but less well in the real world).

This paper reports protocol version 5 (4 July 2018). Ethical approval for the SUPERB trial was obtained on 31 January 2017. Approvals from all sites to begin recruitment were completed by 6th April 2017. Recruitment, in terms of identification of potential participants, was completed on 31st August 2018, with the last participant consented to the study 9th October 2018.

## Additional files

Additional file 1: SPIRIT 2013 Checklist: Recommended items to address in a clinical trial protocol and related documents. (DOC 122 kb)

Additional file 2: TiDieR table for the SUPERB trial intervention. (DOCX 29 kb)

Additional file 3: SUPERB Full Study Protocol (SPIRIT 2013 Checklist page numbers relate to this). (PDF 1790 kb)

## Abbreviations

CRN: Clinical research nurse
CSRI: Client Service Receipt Inventory
DISCS: Depression Intensity Scale Circles
DMC: Data Management Committee
EQ-5D-5L: European Quality of Life measures, 5 dimensions, 5 levels
GHQ-12: General Health Questionnaire 12 item version
GHQ-28: General Health Questionnaire 28 item version
ICC: Intra-class correlation coefficient
KCTU: King’s Clinical Trials Unit
MDT: Multi-disciplinary team
MRC: Medical Research Council
PIN: Participant identification number
QALYs: Quality-adjusted life years
RCT: Randomised controlled trial
SLT: Speech and language therapist
SO: Significant other
SPIRIT: Standard Protocol Items: Recommendations for Interventional Trials
TSC: Trial Steering Committee
