# Supplementary material for: Adjustment with aphasia after stroke: study protocol for a pilot feasibility randomised controlled trial for SUpporting wellbeing through PEeR Befriending (SUPERB)
Source: Pilot Feasibility Stud. 2019 Jan 22;5:14. doi: 10.1186/s40814-019-0397-6 (PMC6341752; doi:10.1186/s40814-019-0397-6)
Supplement: Supplementary file 2 — TiDieR table for the SUPERB trial intervention. (DOCX 29 kb) [file 40814_2019_397_MOESM2_ESM.docx]

**TiDieR table for the SUPERB trial intervention**

| Item 1: Brief name | Supporting wellbeing through PEer Befriending (SUPERB) |
| --- | --- |
| Item 2: Why | Peer befriending may have the potential for improving the psychosocial wellbeing for people with stroke and aphasia. Peer befriending is social and emotional support provided by people with experience of a condition to others sharing a similar condition to bring about a desired social or personal change.^1^ Peer befrienders, who have achieved improvements in their own condition, have been found to offer acceptance, respect, empathy, support, companionship and hope and share experiences and ideas about how to cope.^2^ Peer befriending aims to utilise the skills, knowledge and ‘lived experience’ of people with longer-term aphasia to offer emotional, social and informational support to others with aphasia, starting at a time of transition (discharge from hospital and withdrawal of intensive therapeutic input) and increased need. It aims to help people move forward and develop their own strategies for adjusting to life post-stroke. |
| Item 3: What: materials | Peer-befrienders will receive an established package of training based on an adapted peer befriending intervention manual from Connect-the communication disability network, now Re-Connect.^3^ The training manual will cover a range of topics related to peer befriending (e.g. the role of a befriender, hopes and fears, how to have a conversation as a befriender, goal setting, dealing with challenging situations), health and safety, and dealing with adverse events. Peer befrienders will receive a copy of a peer befriender handbook, which contains key information from the manual (available from authors upon request). The handbook will contain key forms that befrienders will need to complete prior to and following visits (e.g. risk assessment forms, itinerary, record of visit forms, adverse event forms). Peer-befrienders will receive monthly group supervision sessions (from a befriender facilitator), as well as additional individual supervision sessions (face to face or remote) if/when they have additional needs. For supervision sessions, aphasia-friendly agendas and minutes will be available to all participants. For all training and supervision sessions, materials to support communication with people with aphasia will be available (e.g. pens and paper, maps). |
| Item 4: What: procedures | Befrienders will first attend training. Once trained, befrienders will be matched with a person with aphasia who has recently been discharged from hospital. Where possible, matching will take account of preferences around gender, cultural factors, age, and personal interests. Befrienders (and the befriender facilitator) will be provided written aphasia-friendly information about the person they are visiting. The frequency, schedule and nature of visits will be agreed between the pair at their first meeting.  This meeting will also identify possible goals for the intervention.  For example, participants might highlight concerns that they would like to discuss or activities that they would like to pursue.  Subsequent visits may include: conversation, problem solving, trips out e.g. to a local group, and joint activities. Befrienders need to notify the befriender facilitator of each visit including, prior to and following each visit for health and safety reasons. A record of each visit needs to be completed by the peer befriender (with the support of the befriender facilitator). After each visit peer-befrienders will complete an aphasia friendly record sheet, detailing each session held with a participant. This will include whether a visit was cancelled and reason why, length of visit, topics discussed, activities undertaken, any decisions made, and date and time of next visit. Peer-befrienders will complete these sheets as soon as possible after each visit if necessary with the help of the befriender facilitator. Befrienders will attend regular group supervision sessions and seek individual supervision (as required). |
| Item 5: Who provided | Peer befriender training and supervision: Two befriender facilitators provided these. The first person is a qualified speech and language therapist with >20 years’ experience of working with people with aphasia, of which 12 years were spent setting up and managing peer befriending schemes. She is one of the trainers and the befriender facilitator who will supervise all the befrienders throughout the course of the study. The second person is a second trainer who is a Clinical Linguist with 12 years’ experience of facilitating communication for people with aphasia.  Peer befriending intervention: Peer befrienders will have mild-moderate aphasia, be at least one year post-stroke, be able to complete visits over a 3-4 hour period (including travel time), able to use public transport (or drive) and have positive personality characteristics to help with the completion of visits (e.g. resilience, open with feelings, confident, able to approach others). Peer befrienders will also need to have availability to attend training and monthly supervision sessions at a central location. |
| Item 6: How | Peer befriender training and supervision: The training of befrienders occurs face-to-face in groups that are therapist-facilitated. Each group contains up to five befrienders. Group supervision sessions are conducted with up to 10 befrienders face-to-face. Additional individual supervision sessions are completed (as required) by the befriender facilitator by email, telephone, Skype or face-to-face.  Peer befriending intervention: Befrienders organise the date and time of visits with people with aphasia in-person, by telephone or text. The befriender facilitator may provide support to organise visits (if required). Befrienders notify the befriender facilitator of their arrival and departure from a visit by text or telephone. Visits are completed face-to-face between the peer befriender and the person with aphasia being visited. Ideally, the significant other of the person with aphasia would not be present for the visit. Peer befrienders will complete the record sheet after each visit either independently or with support of the befriender facilitator face-to-face, by email, telephone, text or Skype. |
| Item 7: Where | Peer befriending training and supervision: Training and supervision will take place in a quiet, private room at a central location.  Peer befriending intervention: The majority of visits completed by befrienders will occur in a person’s home. In some circumstances the visits will occur out in the community (e.g. local café, stroke club). |
| Item 8: When and how much | Peer befriending training and supervision: The training of befrienders involved 5-6 hours across 2-3 days. Befrienders will attend monthly group supervision sessions of approximately 1-1.5 hours in duration when they are actively befriending people for the duration of the study.  Peer befriending intervention: Each person having befriending will have six visits (each visit a minimum of 1-hour in length) over a 3 month period, plus a further two follow-up visits in the following six months (if appropriate). It is anticipated that each peer-befriender will work with 2-4 participants during the project and no more than two at any one time. |
| Item 9: Tailoring | Peer befriending training and supervision: The same topics were addressed during the training of befrienders but tailoring was done to accommodate the needs and abilities of the group participants (e.g. attention, fatigue, frequent breaks). For example, some befrienders received training in 2-days whereas others received training in shorter periods over 3-days. The minimum amount of detail to be provided about befriending is described in the manual but additional information was given if requested by participants. Supervision sessions were tailored to the needs of the group participants and would often reflect experiences, concerns or challenges raised by befrienders in the previous month or at the beginning of the session. Content from the training was revised during supervision sessions if appropriate and relevant. The pace of training and supervision sessions was also tailored to accommodate the communicative abilities of all people within the group (e.g. slower pace, frequent breaks).  Peer befriending intervention: Visits were tailored to the needs and requests of people with aphasia who were being visited. Conversational topics, location of visits (i.e. at home or in the community) and purpose of visits were some areas tailored. |
| Item 10: Modifications | Peer befriending training and supervision: The length of befriender training was revised down from 6 hours to 5 hours following the first training group. The topics discussed in the training were not altered but less in-depth detail was provided for some, e.g. Health & Safety, Safeguarding, as befrienders found them too detailed and lost interest. These topics were regularly reviewed within supervision sessions as and when issues arose.  Peer befriending intervention: No modifications made. |
| Item 11: How well: planned | Peer befriending training and supervision: Attendance to supervision sessions will be recorded. The fidelity of the peer befriender training and supervision will be evaluated. All training, and all supervision sessions except for the first and last, will be videotaped and rated by unblinded research students against fidelity checklists. Information from the supervision notes will be used to provide context on the fidelity of the intervention.  Peer befriending intervention: Adherence to the intervention will be assessed by means of calculating the number of matches that completed the six visits (in 3 months) and closely recording any problems (e.g. rescheduled or cancelled visits) that may have occurred. The fidelity of the peer-befriending intervention will be evaluated. With participants’ consent a proportion of befriending visits (1 per participant) will also be videotaped and watched by research students against a fidelity checklist. Results from these checks will be reported back to the befriender facilitator to inform later content of the supervision sessions. Information from the peer befriender visit record forms will also be used to evaluate whether peer befrienders followed the peer befriending handbook and compare content of intervention between and within different peer befrienders. All of this information will provide additional qualitative information and context on the fidelity of the intervention. |
| Item 12: How well: actual | To measure fidelity of the peer befriending intervention, videotaped recordings of the training, supervision sessions and visits will be rated against the fidelity checklists. Further data (e.g. record sheets, minutes of supervision sessions) will be qualitatively analysed to give further information about the intervention. Fidelity data will be reported prior to trial completion. |

1. Solomon P. Peer support/peer provided services underlying processes, benefits, and critical ingredients. *Psychiatr Rehabil J.* 2004;27(4):392-401.

2. Mead S, Hilton D, Curtis L. Peer support: a theoretical perspective. *Psychiatric rehabilitation journal.* 2001;25(2):134.

3. McVicker S, Swinburn K. *How to set up a befriending Scheme: a guide for scheme organisers.* UK: Connect Press; 2009.
