# Supplementary material for: Adjustment with aphasia after stroke: study protocol for a pilot feasibility randomised controlled trial for SUpporting wellbeing through PEeR Befriending (SUPERB)
Source: Pilot Feasibility Stud. 2019 Jan 22;5:14. doi: 10.1186/s40814-019-0397-6 (PMC6341752; doi:10.1186/s40814-019-0397-6)
Supplement: Supplementary file 3 — SUPERB Full Study Protocol (SPIRIT 2013 Checklist page numbers relate to this). (PDF 1790 kb) [file 40814_2019_397_MOESM3_ESM.pdf]

CONFIDENTIAL RESEARCH PROTOCOL

Adjustment with aphasia after stroke:

Exploring the feasibility of a definitive phase III RCT for SUpporting wellbeing through PEeR Befriending (SUPERB)

Protocol Version 5 (4th July 2018)

IRAS Reference Number: 220281

Funded by:

Funders Reference Number: PPA2015/03

Sponsored by:

Sponsors Reference Number: Staff/16-17/06

ClinicalTrials.gov Identifier: NCT02947776

TABLE OF CONTENTS

ABBREVIATIONS **5**

GENERAL INFORMATION **7**

TRIAL COMMITTEES **9**

PROTOCOL AMENDMENTS **11**

TRIAL SUMMARY **13**

1. INTRODUCTION **15**

1.1 BACKGROUND & RATIONALE 15
1.2 TRIAL OBJECTIVES 16
1.3 TRIAL DESIGN 17
1.4 TRIAL FLOWCHART 19

2. METHODS: PARTICIPANTS, INTERVENTIONS, AND OUTCOMES **21**

2.1 STUDY SETTING 21
2.2 ELIGIBILITY CRITERIA 21
2.2.1 Inclusion criteria 21
2.2.2 Exclusion criteria 22
2.3 INTERVENTIONS 23
2.3.1 Description 23
2.3.2 Fidelity 24
2.3.3 Adherence / Compliance 25
2.4 OUTCOMES 25
2.4.1 Feasibility endpoints 26
2.4.2 Primary outcome endpoints (self-report measures) 26
2.4.3 Secondary outcome endpoints (all self-report measures) 26
2.4.4 Exploratory outcome endpoints (self-report) 27
2.4.5 Economic evaluation outcome measures for participants with aphasia 27
2.5 PARTICIPANT TIMELINE 28
2.6 SAMPLE SIZE 38
2.7 RECRUITMENT 39

3. METHODS: ASSIGNMENT OF INTERVENTIONS (FOR CONTROLLED TRIALS) **39**

3.1 ALLOCATION 39
3.2 BLINDING (MASKING) 39

4. METHODS: DATA COLLECTION, MANAGEMENT, AND ANALYSIS **41**

4.1 DATA COLLECTION METHODS 41
4.2 DATA MANAGEMENT 42
4.2.1 Trial databases 42
4.2.2 Database passwords 42

4.2.3 Data Handling & Confidentiality/Format of Records 43
4.2.4 Identifiable and Unblinded Data 43
4.2.5 On-Site/Central Monitoring 44
4.3 STATISTICAL METHODS 44
4.3.1 General 44
4.3.2 Descriptive statistics 44
4.3.3 Feasibility outcomes 44
4.3.4 Primary outcome analysis 45
4.3.5 Secondary and exploratory outcome analysis 46
4.3.6 Missing data 46
4.3.7 Safety outcomes 46
4.3.8 Per protocol analysis 46
4.3.9 Qualitative data 47
4.3.10 Economic evaluation 47
5. METHODS: MONITORING 48
5.1 DATA MONITORING 48
5.1.1 Stopping guidelines 48
5.2 HARMS 48
5.2.1 Reporting AEs, SAEs, SARs and SUSARs 49
5.2.2 Follow-up after adverse events 50
5.3 AUDITING 51
6. ETHICS AND DISSEMINATION 51
6.1 RESEARCH ETHICS APPROVAL 51
6.2 PROTOCOL AMENDMENTS 51
6.3 CONSENT OR ASSENT 51
6.4 PROGRESSING TO A DEFINITIVE TRIAL 52
6.5 CONFIDENTIALITY 52
6.6 DECLARATIONS OF INTERESTS 52
6.7 ACCESS TO DATA 52
6.8 ANCILLARY AND POST-TRIAL CARE 53
6.9 DISSEMINATION POLICY 53
7. INSURANCE / INDEMNITY 53
8. FINANCIAL ASPECTS 54
9. SIGNATURES 54
10. REFERENCES 55
11. APPENDIXES 59
11.1 APPENDIX 1 – DEVELOPMENT PHASE 59
11.2 APPENDIX 2 – DESCRIPTION OF OUTCOME MEASURES 61

11.2.1 Participant with aphasia outcomes *61*
11.2.2 Significant others outcomes *63*
11.2.3 Peer befriender outcomes *63*
11.3 APPENDIX 3 – FIDELITY CHECKLISTS 65
11.4 APPENDIX 4 – AE, SAE AND SUSAR FORMS 74
11.5 APPENDIX 5 – EXAMPLE CONSENT FORM 82

Abbreviations

AE	Adverse Event
BAT	Behaviour activation therapy
BCOS	Bakas Caregiving Outcome Scale
CALM	Communication and Low Mood
CCRSA	Communication Confidence Rating Scale for people with Aphasia
CI	Chief Investigator
CIQ	Community Integration Questionnaire
CLQT	Cognitive Linguistic Quick Test
CPIB	Communication Participation Item Bank
CRF	Case Report Form
CRN	Clinical Research Network
CSRI	Client Service Receipt Inventory
CTU	Clinical Trials Unit
DISCS	Depression Intensity Scale Circles
DMC	Data Management Committee
eCRF	Electronic case report form
EQ-5D-5L	European Quality of Life measure (5 dimensions, 5 levels)
ESD	Early supported discharge
FAST	Frenchay Aphasia Screening Test
FS	Friendship Scale
GCP	Good Clinical Practice
GP	General Practitioner
GSE	General Self-Efficacy Scale
GHQ	General Health Questionnaire
HRA	Health Research Authority
ICC	Intraclass Correlation Coefficient
ICER	Incremental Cost Effectiveness Ratio
KCTU	Kings Clinical Trial Unit
MDT	Multi-Disciplinary Team
NIHR	National Institute for Health Research
NHS	National Health Service
NRES	National Research Ethics Service
PEER	Peer Befriending arm of the trial
PIN	Patient Identification Number
PwA	Person with aphasia
QALY	Quality Life Adjusted Years
RCT	Randomised controlled trial
SAE	Serious adverse event
SAR	Serious Adverse Reaction

SOP	Standard Operating Procedure
SLT	Speech and Language Therapist
SUPERB	SUpporting wellbeing through PEeR Befriending
SUGAR	Service User and carer Group Advising in Research
SUSAR	Suspected Unexpected Serious Adverse Reaction
SWEMWBS	Short Warwick Edinburgh Mental Well-Being Scale
TM	Trial Manager
TMC	Trial Management Committee
TSC	Trial Steering Committee
UGC	User Group Committee
USUAL	Usual care arm of the trial
WAB-R	Western Aphasia Battery – Revised
WEMWBS	Warwick Edinburgh Mental Well-Being Scale

General Information

Chief Investigator

Prof Katerina Hilari
Professor of Acquired Communication Disorders
Language and Communication Science
School of Health Sciences
City, University of London
Northampton Square, London, EC1V 0HB
Tel: +44 (0)20 7040 4660
Email: k.hilari@city.ac.uk

Co-investigators

Prof Jane Marshall
Professor of Aphasiology
Language and Communication Science
School of Health Sciences
City, University of London
Northampton Square, London, EC1V 0HB
Tel: +44 (0)20 7040 4668
Email: j.marshall@city.ac.uk

Prof Alan Simpson
Professor of Collaborative Mental Health Nursing Centre for Mental Health Research
School of Health Sciences
City, University of London
Northampton Square, London, EC1V 0HB
Tel: +44 (0)20 7040 5937
Email: a.simpson@city.ac.uk

Dr Shirley Thomas
Associate Professor in Rehabilitation Psychology
University of Nottingham
Division of Rehabilitation and Ageing
School of Medicine, Floor B
The Medical School, Queen's Medical Centre
Nottingham NG7 2UH
Tel: +44 (0)115 846 7484
Email: Shirley.thomas@nottingham.ac.uk

Dr Chris Flood
Senior Lecturer
School of Health Sciences
City, University of London
Northampton Square, London, EC1V 0HB
Tel: +44 (0)20 7040 5989
Email: c.flood@city.ac.uk

Dr Sarah Northcott
Research Fellow
Language and Communication Science
School of Health Sciences
City, University of London
Northampton Square, London, EC1V 0HB
Email: Sarah.northcott@city.ac.uk

Dr Sally McVicker
Former CEO of Connect
Re-Connect at City
Language and Communication Science
School of Health Sciences
City, University of London
Northampton Square, London, EC1V 0HB
Email: sally.mcvicker@city.ac.uk

Dr Kimberley Goldsmith
Senior Lecturer in Medical Statistics
Biostatistics Department
Division of Psychology and Systems Sciences
Institute of Psychiatry, Psychology & Neuroscience
King's College London
De Crespigny Park
London, UK
Tel: +44 (0)20 7848 0059
Email: kimberley.goldsmith@kcl.ac.uk.

Trial Manager

Dr Nicholas Behn
Research Fellow
Language and Communication Science
School of Health Sciences
City, University of London
Northampton Square, London, EC1V 0HB
Tel: +44 (0)20 7040 0884
Email: Nicholas.behn@city.ac.uk

Research Sponsor Contact

Ms Alison Welton

Research Governance Officer
School of Health Sciences
City, University of London
Northampton Square, London, EC1V 0HB
Tel: +44 (0)20 7040 5704
Email: A.Welton@city.ac.uk

Trial Committees

Trial Management Committee (TMC)

The TMG will comprise the Chief Investigator and Co-investigators, the trial manager and junior statistician (KJ) who will meet face-to-face monthly to manage the project. The TMC will sign off the protocol and agree all standard operating procedures (SOPs) before the start of recruitment. The committee will also provide overall supervision of the trial including trial progress, adherence to protocol, patient safety and consideration of new information. The committee form a strong multidisciplinary team who will report to the Trial Steering Committee (TSC). The TMG will be kept informed of TSC and DMC advice and consulted by email or teleconferences as required.

Members: Prof Katerina Hilari
Prof Jane Marshall
Prof Alan Simpson
Dr Shirley Thomas
Dr Chris Flood
Dr Sarah Northcott
Dr Sally McVicker
Dr Kimberley Goldsmith
Miss Kirsty James
Dr Nicholas Behn

Prof Hilari, Prof Marshall and Dr Northcott are speech & language therapists with a strong record on longitudinal studies on quality of life and wellbeing in people with stroke and aphasia; on evaluating a range of interventions for aphasia; and on outcome measurement. Prof Hilari will oversee study coordination and management, data analysis, and dissemination. Dr Thomas is a health psychologist who has published widely on post-stroke depression. She led the CALM RCT study, the largest study of its kind, on psychological therapy for people with aphasia and depression. Simpson is Professor of mental health with extensive experience of running large studies and evaluating new and complex interventions. He led the UK's first pilot trial on the clinical and cost effectiveness of peer support in people discharged from mental health units. He also established the award-winning Service User and carer Group Advising on Research (SUGAR). In addition, both Professor Simpson and Dr Northcott have experience of carrying out qualitative research, including evaluating novel interventions. Dr Flood has expertise in conducting health economic evaluations alongside RCTs. Dr McVicker, Chief Executive of Connect and speech and language therapist, has extensive experience of setting up and running peer support programmes; training people with aphasia as befrienders; and working collaboratively with people with aphasia. Dr Goldsmith and Ms James are both statisticians who will oversee the electronic data records and complete the analysis of outcomes. Dr Behn is a speech and language therapist employed as the trial manager with experience in running treatment research projects for people with acquired neurological conditions.

A sub-committee comprised of Prof Katerina Hilari, Prof Jane Marshall and Dr Nicholas Behn will meet on a weekly to fortnightly basis to discuss day-to-day management of the project and any problems that arise. Each of these people are situated within the same division at City, University of London making it easier to organise meetings or discuss problems or concerns that may arise on a daily to weekly basis. This committee will monitor the conduct and progress of the trial, ensure that the protocol is closely adhered to and take appropriate action to safeguard participants when problems occur.

Trial Steering Committee (TSC)
The TSC will consist of an independent chair and independent and non-independent members, as well as the trial manager and key partners from our recruiting sites

Chair: Dr Rebecca Palmer, Speech and Language Therapist, University of Sheffield
Members: Professor Marian Brady, Speech and Language Therapist, Glasgow Caledonian University
Professor Linda Worrall, Speech and Language Therapist, University of Queensland
Professor Jill Francis, Health Psychologist, City, University of London
Professor Mireia Jofre-Bonet, Health Economist, City, University of London
Ms Clare Rossiter, Speech and Language Therapist, Royal London Hospital
Ms Caroline Watchurst, Lead Research Practitioner, University College London Hospital
Ms Laura Howaniec, Lead Research Practitioner, Royal London and Homerton Hospitals
Dr Nicholas Behn, Trial Manager, City, University of London

This group will meet six times during the project to oversee the study management. They will also be consulted via email as and when needed. They will be responsible for reviewing and approving trial documentation (e.g. protocol, information sheets and consent forms) and oversee the conduct of the study, including advising on continuing or stopping the study in light of advice from the DMC (see below). This committee will advise on processes (e.g. fidelity checking; monitoring and reporting adverse events) and issues (e.g. recruitment to target; site specific issues) as they arise.

Data Management Committee (DMC)
The DMC will consist of an independent from the study chair; an independent specialist with interest in trials for people with stroke and aphasia; and an independent statistician. This committee will meet four times to approve the data management plan, monitor adverse events, and to monitor the progress of the trial in relation to safety and ethical issues, including recruitment, uptake of the intervention, withdrawal from the trial, descriptive summaries of the outcomes and any other variables they feel are critical to trial monitoring. They will meet again on completion of data collection. The DMC and the trial statistician will discuss the level of access to unblinded data and will include this level of access and any procedures for unblinding the DMC in the DAMOCLES charter that will be agreed at the first meeting. The DMC will report to the TSC and may advise the TSC to continue or to stop the trial should they feel this is necessary based on their monitoring of the data.

- Dr Nick Drey (Chair), City, University of London
- Dr Steven Bloch, University College London
- Dr Richard Hooper, Queen Mary's University of London

The first person (ND) is independent from the study but is employed by the sponsor. They will chair the DMC. The second member (SB) is an independent specialist. The third person (RH) is a statistician who is independent from both the sponsor and study.

The User Group Committee (UGC)
The UGC will be comprised of four people with aphasia and one significant other. The group will meet 5 times and will advise on management issues; the implications of the findings; and dissemination to the stroke community. Our dissemination will also involve service users, e.g. we will distribute accessible bulletins and a results leaflet to participants and will hold a dissemination day for everyone involved in the study.

Protocol Amendments

Protocol amendments since version 1.0

Protocol version 1.0, 14 October 2016 amended to version 2.0, 21 October 2016. Summary of main changes:

I. Page 1: Clinicaltrials.gov identifier added

II. Section 7.3. Consent or assent: Our original consent process specified that for participants who had such severe aphasia that they were unable to give their own fully informed consent, consultee declaration would be sought. The Bloomsbury National Research Ethics Service (NRES) Committee advised us that a Mental Capacity Act (MCA) flagged committee should evaluate any study involving consultee declaration. To go under a MCA flagged committee researchers need to meet certain criteria, one of which is: "*B2. Justify the inclusion of adults unable to consent for themselves. It should be clear why the research could not be carried out as effectively if confined to adults capable of giving consent.*" We could not justify this criterion, in this project. People able to consent are better candidates to see if the intervention is feasible and has the potential to benefit people with aphasia. If our feasibility study has positive outcomes, then we have grounds to justify including people unable to consent in a larger trial. For this reason, the consultee declaration process was removed.

Protocol amendments since version 2.0

Protocol version 2.0, 21 October 2016 amended to version 3.0, 28 November 2016. Summary of main changes:

I. Page 1: Amended IRAS reference number added

II. Page 9 & 10: Committee members' names completed

Protocol amendments since version 3.0

Protocol version 3.0, 28 November 2016 amended to version 4.0, 13 January 2017. Summary of main changes:

I. Table of contents moved from p.10 to p.2 with headings allocated to administrative and introductory information and added to the table of contents (i.e. abbreviations, general information, trial committees, protocol amendments and trial summary).

II. Page 11: Protocol amendments section added

III. Titles of Case Report Forms (CRFs) changed to correspond with electronic CRFs for participants with aphasia (p.27-30), significant others (p.31-33) and peer befrienders (p.34-36). For example, "screening CRF" changed to "registration and eligibility CRF".

IV. The Bloomsbury NRES Committee returned a provisional decision on 19th December 2016, where they strongly recommended to reconsider the necessity of the pilot (n=8) and start the feasibility study as soon as possible. This was because they felt that during our development phase prior to the pilot we had done "a very good job of planning the research" and "had already addressed any anticipated problems that may have arisen once the study began". After consultation with the TMC, TSC, DMC, the Sponsor and the Funder it was agreed to follow the NRES Committee recommendation and proceed to the feasibility trial without the pilot. All references to the small pilot preceding the feasibility trial were removed from the protocol.

Protocol amendments since version 4.0

Protocol version 4.0, 13 January 2017 amended to version 5.0, 27 April 2018. Summary of main changes:

Minor reorganisation and editing throughout to clarify methods and minimise repetition between sections.

I. The title of the "connect trained facilitator" was changed to "befriender facilitator" throughout. The charitable organisation "Connect" no longer exists and so the title was changed to reflect this.

II. Page 9 & 10: Changes to committee members details

III. Section 1.3: Economic evaluation changed to pilot economic evaluation. Criteria for purposive sampling for qualitative interviews added. Completion of befriending cycles clarified.

IV. Section 2.1, 2.2, 2.5, 2.7: Amendments made to describe circumstances in which a person may be screened a second time. As the trial commenced, it was practical to define circumstances in which a person may be approached a second time. If a person expresses interest in the study but does not meet specific eligibility criteria that may change over time (i.e. low level of emotional distress, corrected visual or hearing problems, or discharged within the borough of the recruiting hospital) they will be asked for their consent to be contacted again in the community for a second screen. Amendments made for the screening and consent of people identified in the community including those screened a second time to be completed by either CRNs (within GP practices), community SLT or members of the research team. Geographical location more clearly defined.

V. Sections 2.3.1, 2.3.2, 2.5: Additional detail added on intervention and training, and fidelity checking.

VI. Section 2.4.1: Additional feasibility endpoints included (i.e. proportion eligible at first screen, proportion eligible at second screen).

VII. Section 2.4.3, 2.5: Outcomes for peer-befrienders are assessed before they start befriending and after two cycles of befriending. For the purposes of determining when to complete post assessment with peer befrienders when befriending cycles are not completed (e.g. person with aphasia dies or withdraws), a cycle of befriending was more clearly defined as a minimum of two visits.

VIII. Section 3.1: Pathway post-discharge removed as a minimisation factor as the majority of participants across all recruitment sites were planned to receive early-supported discharge (ESD).

IX. Section 4.3.3: Additional detail added on feasibility outcomes and analysis to reflect changes in 2.3.2 (fidelity of supervision) and section 2.1 (second screen). Analysis of informal data collected from recruiting teams and sites on their experiences of the trial was added to provide further context to the feasibility outcomes, following a recommendation from the Trial Steering Committee.

X. Section 4.3.10: The panel from The Stroke Association raised some concerns about the economic evaluation. In response to these concerns, minor amendments were made to this section of the protocol. These amendments do not alter the collection or analysis of economic evaluation data but rather clarify that the study is a *pilot* economic evaluation intended to see if we can collect all the costs' data and test the instruments to ensure we are ready for any eventualities in a future complete cost-effectiveness study.

XI. Section 5.2.1: Notification of SAEs, SARs or SUSARs to King's CTU has been removed. When the first SAE was reported, it became apparent that King's CTU only manages SAEs, SARs and SUSARs in cases where the trial is being managed by the CTU. For the SUPERB study, the trial is managed by City, University of London and therefore, it is the responsibility of the Sponsor (i.e. City) to manage these events. Relevant modifications to the report forms have been completed in Appendix 4 of the protocol to reflect these changes.

XII. Section 6.3: Example of a consent form included in Appendix 5.

Trial Summary

Background: Stroke and aphasia can have a profound impact on people's lives. Depression is a common sequel of stroke, with rates remaining high even one year after stroke at 33%. It is associated with worse rehabilitation outcomes, increased carer strain, increased healthcare utilisation, and higher mortality. A recent audit of clinical psychology services for people with mood problems post-stroke across 10 UK stroke services found that the most common outcome of mood assessment was monitoring and advice, with less than half of patients with low mood receiving psychological intervention. There is a pressing need to evaluate systematically interventions that aim to improve psychosocial wellbeing for people with stroke and aphasia.

Aims: SUPERB trial aims to evaluate the feasibility of a phase III trial on the clinical and cost-effectiveness of a stepped care model level 1 intervention, peer befriending, for people with aphasia. Specifically it will:

1. Explore the feasibility of a definitive phase III randomised control trial (RCT) based on a) feasibility of recruitment and retention to the trial, b) acceptability of research procedures and outcome measures, c) acceptability of usual care + peer befriending vs. usual care control to participants, their significant others and peer support workers, d) documentation of usual care, e) treatment fidelity of peer befriending.
2. Explore psychological and social well-being outcomes as outcomes in a definitive trial for a) people with aphasia receiving usual care + peer befriending versus usual care control, b) their significant others, and c) peer befrienders.
3. Explore the feasibility of a full economic evaluation of usual care + peer befriending versus usual care control in a phase III RCT.

Intervention to be tested: The intervention group will receive peer befriending from stroke survivors with long-term aphasia who will be trained as peer befrienders. They will visit participants who have had a stroke more recently 6 times over a period of 3 months. A further 2 visits within the next 6 months will also be offered for a gradual transition to the end of the peer befriending. The schedule and nature of visits will be agreed between the pair at their first meeting. This meeting will also identify possible goals for the intervention e.g. to offer conversation, help with problem solving and social activities.

Methods: The overall study comprises a development phase and a phase II RCT. The development phase will inform the intervention manual, the choice of outcome measures, fidelity practices, and topic guides for participant interviews. This phase will be informed through a series of six workshops attended by six people with aphasia with experience of peer befriending.

The phase II pilot feasibility RCT will be a single blind, mixed methods, parallel group design comparing usual care (n=30) with usual care + peer befriending (n=30) for people with aphasia post-stroke and low levels of psychological problems. Assessments and outcome measures for participants and significant others will be administered before randomisation with outcome measures re-administered at 4 and 10 months post-randomisation. Peer befrienders will complete outcome measures before training and after they have completed the visits for two participants. In addition, this RCT will include a qualitative study and a pilot

economic evaluation. The qualitative study will use semi-structured interviews of purposively sampled participants (n=20) and significant others (n=10) from both arms of the trial, and all peer befrienders. The pilot economic evaluation will utilise the EQ-5D and a stroke-adapted version of the CSRI.

Outcomes: RCT: feasibility of recruitment of participants to definitive trial (including proportion screened who meet criteria; proportion who consent; rate of consent); number of missing/incomplete data on outcome measures; attrition rate at follow-up; potential value of conducting main trial using value of information analysis (economic evaluation); description of usual care; treatment fidelity of peer befriending. Patient-reported outcomes will include mood, wellbeing, communication and social participation. Qualitative study: participant, significant other, peer befriender views on acceptability of procedures and experiences of care received. Economic evaluation: cost outcomes, average costs, costs per participant and mean difference between trial arms, description of resources used and overall cost effectiveness.

Benefits: This study will provide feasibility level evidence on whether peer befriending is a suitable intervention to explore further, in terms of averting some of the serious psychological consequences of stroke, and preventing the need for more complex and costly psychological therapies.

1. INTRODUCTION

1.1 Background & Rationale

Stroke and aphasia can have a profound impact on people's lives. Depression is a common sequel of stroke, with rates remaining high even one year after stroke at 33%.[1] It is associated with worse rehabilitation outcomes, increased carer strain, increased healthcare utilisation, and higher mortality.[2-4] Yet, the Stroke Association's report 'Feeling overwhelmed'[5] highlights that over half of stroke units in England still have no access to psychology services; and that two thirds of stroke survivors felt their emotional needs were not as well looked after as their physical needs.

There is evidence that the psychological needs of people with aphasia are even greater than in the general stroke population. Rates of depression in this group can rise to 62%[6] and family units often fall under strain.[7] Social support is also affected. People with aphasia take part in fewer social activities;[8] and are at risk of losing contact with friends and their wider social network.[9,10] This is particularly concerning, as poor social support is associated with worse physical recovery,[11] and increased likelihood of a future adverse event such as a second stroke.[12] It is therefore paramount to support psychosocial well-being post stroke and aphasia.

A recent audit of clinical psychology services for people with mood problems post-stroke across 10 UK stroke services found that the most common outcome of mood assessment was monitoring and advice, with less than half of patients identified as having low mood receiving psychological intervention.[13] The National Clinical Guidelines for Stroke[14] and the NHS Stroke Improvement Programme[15] highlight that psychological care after stroke should be multifaceted, involving many agencies - health, social care, voluntary; and recommend a stepped care approach. Stepped care seeks to ameliorate problems with access through better allocation of scarce psychological therapy resources.[16] The model aims to offer psychological care in a hierarchical approach, offering simpler interventions first (level 1), such as those provided by peers or stroke specialist staff, and progressing to more complex interventions requiring input from clinical psychology or psychiatry (level 3) if required.[15] A Cochrane systematic review has indicated that there is currently insufficient evidence for the effectiveness of psychological therapies for treating post-stroke depression.[17] There is a pressing need to systematically evaluate interventions that aim to improve psychosocial wellbeing for people with stroke; and for the vulnerable group of people with aphasia, who are often excluded from stroke studies, in particular.

The proposed study aims to address this need for people with aphasia, within the stepped care model. Current evidence is limited. No level 3 interventions have been evaluated with people who have aphasia following stroke. One study has explored a level 2 intervention: a multicentre randomised controlled trial (n=105) of a behaviour activation therapy (BAT) delivered by assistant psychologists for treating depression in stroke patients with aphasia and found that mood was significantly better at 6 months follow up in those who received BAT compared to usual care (Communication and Low Mood; CALM trial).[18] Level 1 interventions have not been evaluated with people who have aphasia. Evidence of benefit at this level would be particularly welcome, as these interventions might help to avert some of the serious psychological

consequences of stroke, and prevent the need for more complex and possibly more costly level 2 and 3 input.

A level 1 intervention with potential for application in the field of stroke and aphasia is one to one peer support / peer befriending which is widely used in mental health[19] and other long-term conditions.[20] Peer befriending is social and emotional support provided by people with experience of a condition to others sharing a similar condition to bring about a desired social or personal change.[21] Peer befrienders, who have achieved improvements in their own condition, offer acceptance, respect, empathy, support, companionship and hope and share experiences and ideas about how to cope.[22] A recent systematic review and meta-analysis found moderate but significant positive effects of befriending on depressive symptoms [standard difference in means in depressed elderly people SMD=-0.75].[23] Peer befriending has been explored but not formally evaluated in people with aphasia.

Connect, the communication disability network, is a charity for people with aphasia, providing information and support, training, and helping people with aphasia develop and deliver the services they want. Over 50 people with aphasia have been trained by Connect as peer-befrienders and over 130 have been reached by their befriending service. The scheme has mostly targeted those in the longer term post-stroke who were socially isolated with poor access to support systems. Connect has explored participants' views about the scheme which are overwhelmingly positive for those receiving the scheme (emotional support, hope, encouragement, motivation, tips), their families (support and time out, seeing the person with aphasia differently, hope and encouragement about the future), peer-befrienders (new skills, feeling useful and offering something, being part of a network of support), and health professionals (building a low-cost, flexible and sustainable service option delivered by users). The proposed study aims to refine this scheme in order to offer it to more people with aphasia, at a time of increased need when they are discharged to the community from hospital [24] and active care is withdrawn, and begin its evaluation in a systematic way. The study will evaluate the feasibility of a study on the clinical and cost-effectiveness of peer befriending for people with aphasia post-stroke.

1.2 Trial Objectives

The proposed study will evaluate the feasibility of a study on the clinical and cost-effectiveness of peer befriending for people with aphasia post-stroke. We will run an exploratory (phase II) randomised controlled trial (RCT). The study will achieve three main objectives:

- Explore the feasibility of a definitive phase III RCT based on a) feasibility of recruitment and retention to the trial, b) acceptability of research procedures and outcome measures, c) acceptability of usual care + peer befriending vs. usual care control to participants, their significant others and peer befrienders, d) documentation of usual care, e) treatment fidelity of peer befriending.
- Explore psychological and social well-being outcomes as outcomes in a definitive trial for a) people with aphasia receiving usual care + peer befriending versus usual care control, b) their significant others, and c) peer befrienders.

- Explore the feasibility of a full economic evaluation of usual care + peer befriending versus usual care control in a phase III trial.

1.3 Trial Design

The main trial will be a single blind (blinded assessors), mixed methods, parallel group pilot feasibility (phase II) multicentre RCT comparing usual care + peer befriending (PEER) to usual care (USUAL) for people with aphasia with low levels of psychological distress after stroke. Recruitment to the main study will begin in the second year of the study following the 1-year development phase (see Appendix 1). Three groups will be participants in the study: participants with aphasia, significant others and peer-befrienders. All participants will be screened for eligibility, will be provided with information about the study and written consent will be obtained from those agreeing to take part. Participants with aphasia will be recruited in a 2-stage consent process (see section 3.2 for full justification of 2-stage consent). As peer befriending is a behavioural, psychological intervention, if details of the intervention tested are revealed from the start, participants in the control arm will know they are in the control arm. This may cause them undue distress and also compromise the validity of the study (see section 3.2). Therefore, in the first stage, eligible participants will provide written consent for inclusion into a study monitoring progression and adjustment to life after stroke and aphasia; and comparing different packages of care. Assessments and baseline measures for participants with aphasia and their significant others will then be administered, followed by randomisation. In the second stage, participants with aphasia randomised in the PEER arm of the trial will be given further information about peer befriending and asked to consent to the peer-befriending intervention. Feasibility measures will be assessed either on an ongoing basis or when appropriate, with outcome measures obtained at 4 and 10 months post-randomisation for both participants and their significant others. This schedule provides a baseline, a post intervention, and a follow up assessment. Assessors who are blind to group allocation will complete these assessments, except in the case of the economic service use data. If these assessors become unblinded they will report this to the trial manager who will keep a record of these instances. Peer-befrienders will complete screening assessments and baseline measures before training and outcome measures after they have completed befriending for two participants.

The trial will include a qualitative component to explore experiences of the study and the intervention. Participants with aphasia, significant others and peer befrienders will complete semi-structured interviews. We will purposively select a sub sample of participants with aphasia (USUAL n=10; PEER n=10), optimising the diversity and range of characteristics that are of relevance. Key sampling criteria will include severity of aphasia and living arrangements. Secondary criteria will include geographical area, gender, mobility, GHQ-12 score and ethnicity. Interviews will be conducted at 4 months post-randomisation for the entire subsample, and for those in the intervention arm at 10 months as well. Significant others (USUAL n=5; PEER n=5) will be purposively selected to include different relationship to person with aphasia (partner/spouse or child/other), ethnicity, gender and GHQ-28 scores, with these interviews also conducted at 4 months post-randomisation. The peer befrienders will be interviewed after they have completed two cycles of befriending (minimum two visits per cycle). The qualitative research assistant, who will be a person different to the assessors above and unblinded to group allocation, will complete qualitative interviews.

Pilot economic evaluation study to consider the relative cost-effectiveness of PEER compared to USUAL will also be conducted. This pilot evaluation will occur at 4 months and 10 months post-randomisation. Pilot data will be collected from participants and significant others using an adapted version of a well-used tool for collecting resource use (Stroke adapted Client Service Receipt Inventory) and information from clinical records provided by clinicians in each site. As resource use will include peer-befriending, which renders the data unblinded, the qualitative research assistant or trial manager will collect this information for the pilot economic evaluation.

1.4 Trial Flowchart

Randomised controlled trial – Persons with aphasia (PwA) and significant others

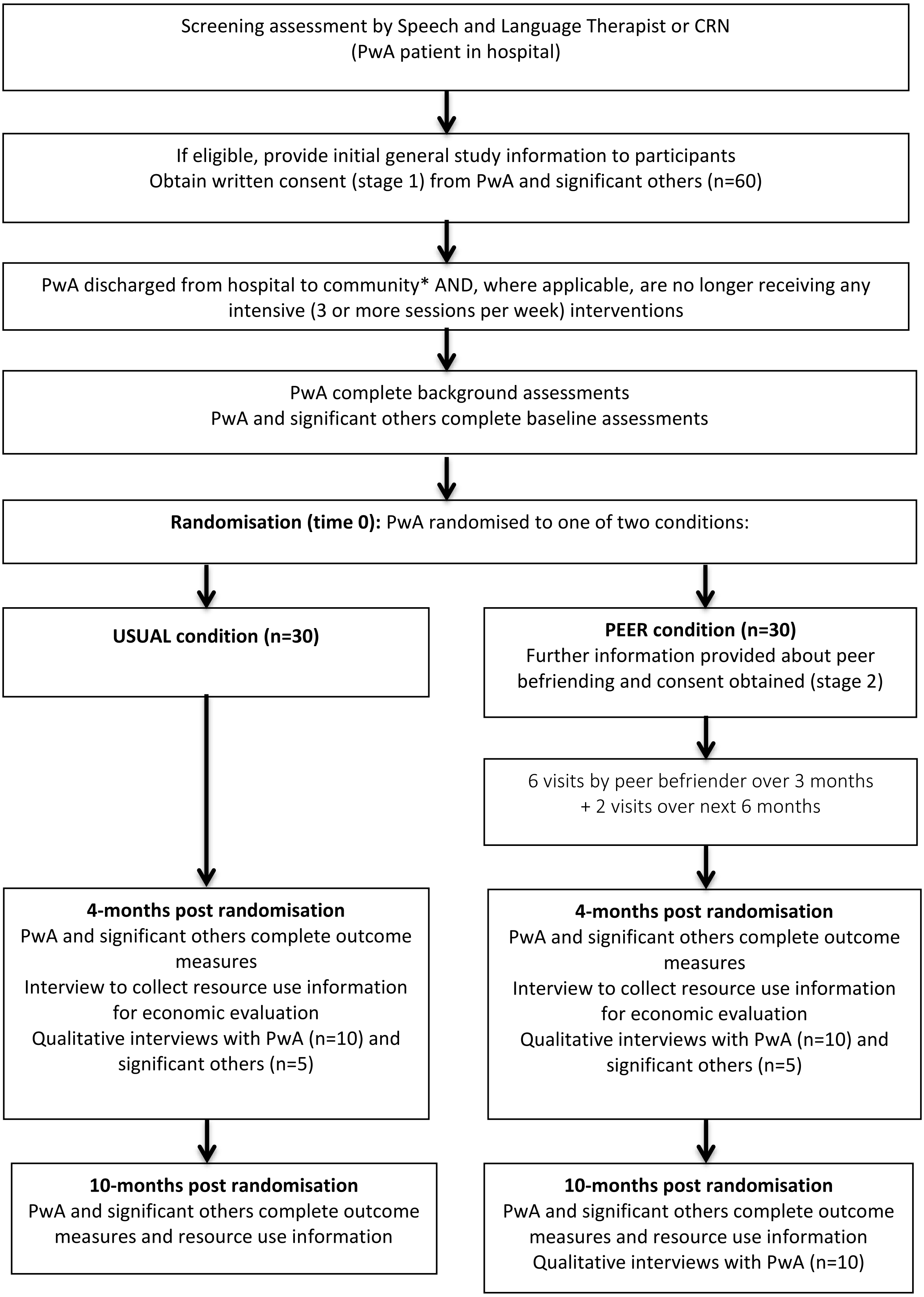

* If CRN/SLT do not manage to screen and consent while person with aphasia is still in hospital, screening and consent can take place at this stage by the community CRN, SLT or member of the research team.

Randomised controlled trial – Peer befrienders

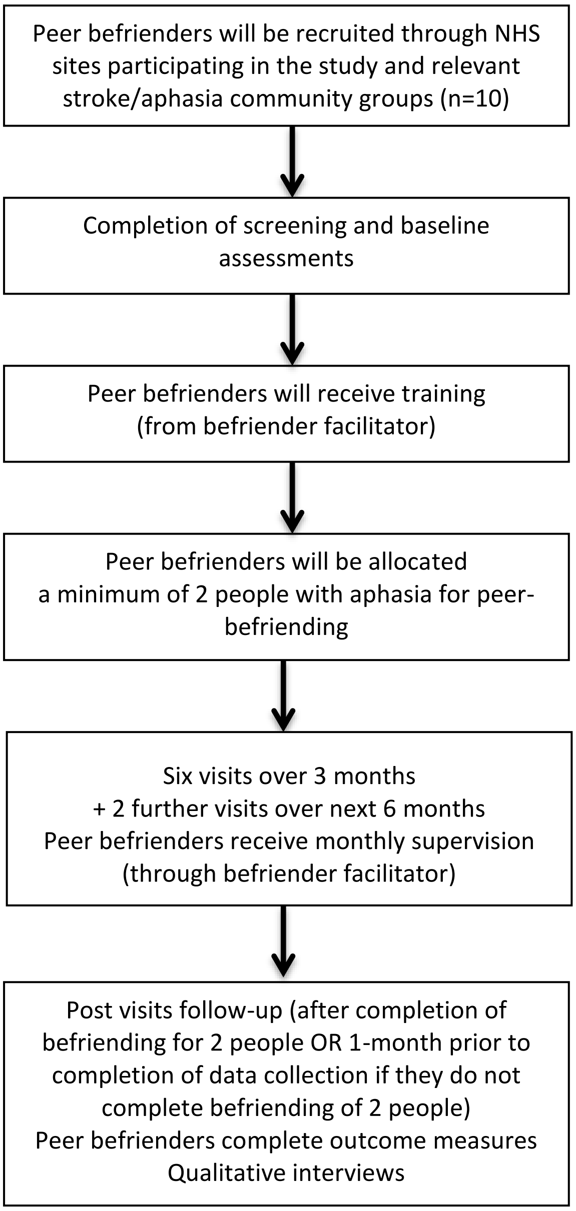

2. Methods: Participants, interventions, and outcomes

2.1 Study setting

Hospital sites will be recruited through the North Thames National Institute for Health Research (NIHR) Clinical Research Network (CRN), which has adopted the study into their portfolio, and through the study team's contacts with Speech and Language Therapy services in hospitals in London. Hospital sites will be excluded if they have active community peer befriending schemes for people with aphasia in place.

People with aphasia and significant others will be screened for eligibility by the Clinical Research Network Nurse (CRN) or the hospital Speech and Language Therapist (SLT) and provide consent for inclusion into the study typically before the person with aphasia is discharged from hospital to the community. Five hospitals from four NHS trusts are involved in this study. Referrals from the community (e.g. SLT practices, GP practices) will also be accepted to maximise recruitment. If a person expresses interest in the study but does not meet specific eligibility criteria that may change over time (i.e. low level of emotional distress, corrected visual or hearing problems, or discharged within the borough of the recruiting hospital) they will be asked for their consent to be contacted again in the community for a second screen. In the community, screening and consent will be completed by a CRN, community SLT or member of the research team. Following consent, people will then attend several baseline appointments following their discharge from hospital, in their own home. These appointments from the research team will start once any intensive community therapy (3 or more sessions per week) they may have is completed (where applicable). The appointments will be with blind assessors to complete assessments at baseline and then at 4 and 10 months post-randomisation. For people in the PEER arm of the study, 6 visits with a peer befriender over a 3-month period will be conducted (i.e. between baseline and 4-month assessment session). A further two visits within the next 6 months will also be offered for a gradual transition to the end of peer befriending. Nominations for potential peer befrienders will be received from recruitment sites (e.g. community SLT, hospital), local services and voluntary organisations (e.g. Stroke Association, Re-connect). Peer befrienders will be screened for eligibility and consented into the study by the Trial Manager. Peer befrienders will be DBS checked and will be trained on peer befriending, health and safety, and reporting adverse events by befriender facilitator(s) experienced in working with people with aphasia.

2.2 Eligibility criteria

Participants will be recruited at the time of discharge from hospital to the community, local to the recruitment site. Some participants may receive intensive input from an early supported discharge team in their community. Such teams provide intensive (i.e. 3 or more sessions weekly) input from a dedicated multi-disciplinary team. For these participants, baseline assessments will occur once this intensive input is completed. For the rest baseline assessments will occur within 1-2 weeks from discharge home.

2.2.1 Inclusion criteria

Participants with aphasia:

- Over 18;

- Fluent premorbid users of English (confirmed by relative or self report);
- Presence of aphasia due to stroke: determined by the multidisciplinary team (MDT) notes, based on SLT diagnosis. In cases of uncertainty, i.e. where there is no SLT diagnosis at the time of recruiting to the trial, CRN will use the Frenchay Aphasia Screening Test (FAST)[25] for screening for aphasia. This test covers four major aspects of language: comprehension, expression, reading and writing. In these cases presence of aphasia will be determined based on the published cut-offs in the FAST manual.
- Low levels of emotional distress. This will ensure that they do not require immediate level 2 or 3 psychological input. To determine the level of emotional distress, the Depression Intensity Scale Circles (DISCS)[26] will be used. This tool is recommended for people with communication problems, cognitive deficits, and visual perception problems post-stroke.[15,27] Scores on DISCS range 0-5, with a score of 0-1 indicating no/low distress and a score of ≥2 used as a cut-off for identifying depression in those with complex disabilities following brain injury.[26] Those scoring 0-1 will be eligible for the study. If a participant scores ≥2 on DISCS, they will be referred back to the MDT for consideration for more complex psychological care as appropriate. People who score 2 (which is also the median on DISCS) and who the MDT deems do not need other psychological care or other psychological care is not available will still be eligible to take part. If a person scores 3 if screened while still in hospital, and is uncertain about their level of emotional distress or feels they will be much better once at home, consent will be obtained to re-screen them for eligibility when they return to the community (i.e. second screen). If at the second screen they score 0-1, or 2 as described above, then they will be eligible to take part.

Significant Others: each participant with aphasia will nominate one significant other, who is their closest confidant and who is over 18 years of age. If participants live alone their significant other should be someone that they see at least once a week. Consent will be sought from significant others to take part in the study to explore what the outcomes are for them. If a significant other does not meet eligibility criteria or does not give consent to take part, we will approach up to three significant others nominated by the person with aphasia. Participants with aphasia without a significant other or whose significant other does not consent to the project will still be eligible to take part.

Peer befrienders: will be people with mild-moderate aphasia who are over 18 years of age, are at least one year post-stroke and meet the selection criteria that will be identified by a group of consultants with aphasia during the development phase. The trial manager who will check eligibility and give initial information about the project to potential peer befrienders is a highly specialist Speech and Language Therapist and will informally assess befrienders aphasia. He will also administer the short FAST to ensure they score a minimum 5/10 for auditory comprehension and 5/10 for verbal expression.

2.2.2 Exclusion criteria

Participants with aphasia, significant others and peer-befrienders will be excluded if they have:

- Other diagnoses affecting cognition or mental health, such as, but not restricted to, advanced Parkinson's Disease, Motor Neurone Disease, Dementia, Clinical Depression (based on medical records for participants with aphasia and self-report for significant others and peer-befrienders, as well as the GHQ-12 as a depression screen for peer-befrienders: will be excluded if they score ≥ 3);
- Severe uncorrected visual or hearing problems (based on medical records for participants with aphasia and self-report for significant others and peer-befrienders);
- Severe or potentially terminal co-morbidities, on grounds of frailty (based on medical records for participants with aphasia and self-report for significant others and peer-befrienders).

Participants with aphasia will also be excluded if they are:

- Discharged to a geographical location away from the borough of the recruiting hospital, as this will make it unfeasible for peer befrienders to visit those in the intervention arm. Participants discharged to a neighbouring borough easily accessible to peer befrienders will be considered. Address details will be used to determine the geographical location a person is being discharged to. Befrienders may also be contacted to check that participants with aphasia are within reasonable proximity (if uncertain).

2.3 Interventions

2.3.1 Description

USUAL group:

The control group will receive usual care, i.e. all health, social care and voluntary services available to them in their borough. It is not known what exactly usual care comprises for people with aphasia who are discharged in the community with low levels of psychological problems, and this project will help to document usual care.

PEER group:

The intervention group will receive usual care + peer befriending. Peer befriending aims to utilise the skills, knowledge and 'lived experience' of people with longer-term aphasia to offer emotional, social and informational support to others with aphasia, starting at a time of transition (discharge from hospital and withdrawal of intensive therapeutic input) and increased need. It aims to help people move forward and develop their own strategies for adjusting to life post-stroke.

Peer-befrienders will receive an established package of training (5-6 hours) based on a peer befriending intervention manual[28] and ongoing supervision and support from befriender facilitator(s). The training will be conducted by two facilitators, who have experience of the peer befriending intervention and in communicating with people with aphasia. The training will cover a range of topics related to peer befriending (e.g. the role of a befriender, hopes and fears, how to have a conversation as a befriender, goal setting, dealing with challenging situations), health and safety, and dealing with adverse events. Peer befrienders will receive a copy of a peer befriender handbook, which contains key information from the manual. It is

anticipated that each peer-befriender will work with 2-4 participants during the project and no more than two at any one time.

Peer-befrienders will be offered monthly group supervision sessions. These sessions will be conducted by a single befriender facilitator (also involved in the training) who is a trained Speech and Language Therapist. These sessions will be an opportunity to share experiences and discuss any difficulties that have arisen. Peer-befrienders who are facing particular challenges e.g. with one participant, will receive supplementary individual supervision from befriender facilitator(s). Peer befrienders and the befriender facilitator(s) will record these challenges. The facilitator will keep detailed written notes to summarise the content of each supervision session, whether individual or group. The content and management of supervision will be delineated during the developmental phase.

Trained peer-befrienders will visit participants 6 times (each visit a minimum of one hour in length) over a period of 3 months. Based on consultation with current Connect peer-befrienders and befriendees, a further 2 visits within the next 6 months will also be offered for a gradual transition to the end of the peer befriending. Participants will be paired with and introduced to their peer-befriender as soon as possible after and within one month of randomisation. If this is not feasible, reasons for this will be recorded. Where possible, pairing will take account of preferences around gender, cultural factors, age, and personal interests. The schedule and nature of visits will be agreed between the pair at their first meeting. This meeting will also identify possible goals for the intervention. For example, participants might highlight concerns that they would like to discuss or activities that they would like to pursue. Subsequent visits may include: conversation, problem solving, trips out e.g. to a local group, and joint activities. The scheme will be informed by the peer befriending handbook, which will detail the peer befriending intervention and will be finalised during the developmental phase. After each visit peer-befrienders will complete an aphasia friendly record sheet (developed in the development phase), detailing each session held with a participant. This will include whether a visit was cancelled and reason why, length of visit, topics discussed, activities undertaken, any decisions made, and date and time of next visit. Peer-befrienders will complete these sheets as soon as possible after each visit if necessary with the help of the befriender facilitator who will collate these sheets during monthly supervision sessions. The facilitator will pass the record sheets onto the Trial Manager on a monthly basis.

2.3.2 Fidelity

We use the term fidelity to indicate whether an intervention is delivered as intended. In this study this applies to the peer befriending training, supervision and the peer-befriending intervention.

<u>Peer befriending training and supervision fidelity</u>

Unblinded researchers from the team and MSc research students will review videotaped sessions of the peer-befriender training sessions and supervision sessions against fidelity checklists (developed during the development phase) to ensure that the content of the training manual is delivered as intended. In addition,

supervision sessions between peer befrienders and befriender facilitator(s) will be monitored (i.e. frequency, individual or group, size of group, topics discussed) to add further qualitative information and context. During the trial, interim results from fidelity of the training and supervision sessions will be used to provide feedback to the befriender facilitator.

Peer befriending intervention fidelity

Information from the peer befriender visit record sheets will be used to evaluate whether peer befrienders followed the peer befriending handbook and compare content of intervention between and within different peer befrienders. With participants' consent a proportion of befriending sessions (1 per participant) will be videotaped and watched against a fidelity checklist (developed during the development phase) to ensure fidelity to the peer befriending handbook. Interim fidelity results will be used to provide feedback to the befriender facilitator to inform later content of the supervision sessions.

The befriender facilitator will monitor and record the monthly group supervision sessions (e.g. length, number of participants, topics discussed). They will also keep a written record of all supervision contact with befrienders, including contact face-to-face, via skype or over the telephone. These supervision records will be provided to the Trial Manager on a monthly basis. They will provide additional qualitative information and context on the fidelity of the intervention.

2.3.3 Adherence / Compliance

Adherence to intervention protocols refers to the degree to which the behaviour of trial participants corresponds to the intervention assigned to them. For participants in the USUAL arm, we will use information reported on the Client Service Receipt Inventory (CSRI) and any information they reveal to our assessors to check their adherence to their allocated arm (i.e. no accessing of peer befriending services). For participants in the PEER arm, primarily data from the peer befriender record sheets will provide us this information. Peer befrienders need to be compliant with a minimum of 6 visits (each one hour long). Peer befrienders should determine the desired mode of contact for making arrangements on the first visit with the participant (i.e. text, telephone, Skype, FaceTime). Befrienders should get participants to record each future visit on a diary and/or calendar and make contact with the patient (through their desired mode of contact) a few days prior to each visit. If any problems arise, visits should be rescheduled. Any cancelled and rescheduled visits should be documented (with a reason if possible). All recorded information should be given to the befriender facilitator during supervision sessions; and subsequently passed on to the trial manager on a monthly basis.

2.4 Outcomes

A range of outcome measures will be used with participants with aphasia covering mood, wellbeing, activities and communication and social participation. The choice of measures has been informed by the views of our consultants with aphasia on the relevance and acceptability of measures. We have also trialled out the outcome measures with our consultants with aphasia to ensure acceptable participant burden. Measures for participants with aphasia have either been developed specifically for neurologically impaired populations,

some including people with aphasia (e.g. see below: DISCS, CPIB, CIQ, CCRSA) or have been previously used with people with aphasia with good evidence of accessibility and acceptability (see below: GHQ-12, FS, EQ-5D). Where appropriate, the presentation of measures will be modified to make them aphasia-friendly in line with best practice guidelines.[29] The content, however, will not be changed to avoid affecting measures' psychometric properties. Each of these outcomes is comprehensively described in Appendix 2.

2.4.1 Feasibility endpoints

- Proportion who are eligible of those screened
- Proportion eligible at first screen
- Proportion eligible at second screen
- The rate of eligibility per month
- Proportion who consent of those eligible
- The rate of consent per month
- The rate of recruitment (participants randomised) per month
- The frequency and proportion of people consented who withdraw overall, by study arm, and by those who do before and after randomisation. This will specifically include describing those in the PEER arm who decline consent at the second stage.
- Acceptability of research procedures and outcome measures based on qualitative interviews
- Acceptability of PEER vs. USUAL care to participants, their significant others and peer support workers, based on qualitative interviews
- Documentation of usual care, based on data from Client Service Receipt Inventory
- Fidelity and adherence, based on session observations, visit record sheets and supervision records

2.4.2 Primary outcome endpoints (self-report measures)

Participant with aphasia measures at baseline, 4 and 10 months post-randomisation
Comparisons will be between PEER and USUAL groups.

- General Health Questionnaire-12 (GHQ-12) continuous total score
- Depression Intensity Scale Circles (DISCS) continuous total score: the DISCS is being measured because some participants may not be able to complete the GHQ-12. This feasibility study will assess how often this is the case. The DISCS will be treated as the primary outcome measure only if there is ≥10% missing data in the GHQ-12 due to severity of aphasia, otherwise DISCS will be a secondary outcome measure.

2.4.3 Secondary outcome endpoints (all self-report measures)

Participant with aphasia measures at baseline, 4 and 10 months post-randomisation
Comparisons will be between PEER and USUAL groups.

- Short Warwick Edinburgh Mental Well-being Scale-7 (SWEMWBS) continuous total score
- Communicative Participation Item Bank (CPIB) continuous total score

- Community Integration Questionnaire (CIQ) continuous total score
- Proportion with high emotional distress vs low emotional distress as measured using the GHQ-12 (high distress = score of 3 or more, low distress = score of 0-2)

Significant other measures at baseline, 4 and 10 months post-randomisation

Comparisons will be between PEER and USUAL groups.

- Warwick Edinburgh Mental Well-being Scale (WEMWBS) continuous total score
- General Health Questionnaire-28 (GHQ-28) continuous total score. The GHQ-28 also provides four subscale scores (somatic symptoms, anxiety/insomnia, severe depression, and social dysfunction), which will be looked at descriptively.
- Bakas Caregiving Outcome Scale (BCOS) continuous total score

Peer befriender measures at baseline and on completion of two befriending cycles

If a befriending cycle is not fully completed because, e.g., a participant with aphasia withdraws or dies, it will be considered completed for the purposes of measuring outcomes with the peer befriender if a minimum of two visits were completed.

- Warwick Edinburgh Mental Well-being Scale (WEMWBS) continuous total score
- Community Integration Questionnaire (CIQ) continuous total score
- General Self-Efficacy Scale (GSE) continuous total score

2.4.4 Exploratory outcome endpoints (self-report)

Participant with aphasia measures at baseline, 4 and 10 months post-randomisation

There are additional psychological constructs that may be affected by peer befriending, however the evidence base is not strong on whether existing measures on these constructs that can be used with people with aphasia are responsive to change. We will include two measures in this study as exploratory measures in order to see whether they may be suitable to use as secondary outcome measures in a definitive trial. As well as the measures above, our consultants with aphasia thought these measures would be accessible to people with aphasia and would capture potential benefits of peer befriending. We will consider reporting these outcomes in the primary report on the study, but they may instead be reported in a subsequent publication.

- Communication Confidence Rating Scale for people with aphasia (CCRSA)
- Friendship Scale (FS)

2.4.5 Economic evaluation outcome measures for participants with aphasia

4 and 10 months post-randomisation

- European Quality of Life measure 5 dimensions, 5 levels (EQ-5D-5L) (self-report; administered at baseline as well)

- Stroke adapted Client Service Receipt Inventory (CSRI, to be completed with significant other as primary respondent where available and participant with aphasia, with additional information drawn from community rehabilitation teams and records by our link clinicians)

2.5 Participant timeline

Completion of recruitment for all 60 participants with aphasia will occur by August 2018 to ensure collection of all follow-up data by the following year. Below is a detailed description of the participant timeline during the RCT phase.

RANDOMISED CONTROLLED TRIAL – People with aphasia

Purpose	Person responsible (Location)	Time	Forms/ Measures	Description
Screening	CRN or SLT (Hospital or home)	20-30 minutes	Registration & eligibility CRF; DISCS	Participants will be initially screened for eligibility based on inclusion and exclusion criteria. Initial information about the study and the need to further screen for potential eligibility will be discussed with verbal consent obtained. Aphasia will be screened if necessary using the Frenchay Aphasia Screening Test[25] (i.e. if no clear information about the presence of aphasia in the medical notes). Distress will be screened with the Depression Intensity Scale Circles (DISCS).[26] This session (and consent) will typically occur while the participant is in hospital however, participants identified in the community (e.g. community SLT, GP practices) who have not already been screened and/or recruited to the study will be considered to maximise recruitment. In addition, people who are being re-approached upon discharge into the community will be screened and/or recruited by the community SLT or member of the research team..
Consent	CRN, SLT or Research team member (Hospital or home)	15-20 minutes	Stage 1 Consent CRF; Stroke case history CRF	This visit will be completed if the participant is eligible for participation in the study. Each eligible participant will be given an information sheet about the study which the CRN, SLT or research team member will go through in detail with the participant. Written consent to participate in the study will then be obtained from the person with aphasia (1st stage consent).
Background & Baseline assessments	Blind assessor (Home)	2-3 sessions (each 1-1.5 hrs)	Personal & social case history CRF; WAB-R; CLQT; GHQ-12; DISCS; SWEMWBS; CPIB; CIQ; EQ-5D-5L; CCRSA; FS	These assessments will occur within 1-2 weeks of discharge home or community setting (including residential care homes and nursing homes); or for those receiving intensive therapy in the community, 1-2 weeks post withdrawal of intensive therapy (3 hours or more per week). The number of sessions to complete the assessments will be dependent on a patient’s aphasia severity and cognitive ability (e.g. level of concentration and fatigue). All assessments and outcome measures will be completed in an interview format with a blind assessor with expertise in facilitating people with aphasia. At the first assessment point, all participants will complete a case history, covering: demographic and health information, family and social circumstances, and personal interests. This information will be used to report on participant characteristics, and will also contribute to pairing with peer-befrienders for those in the intervention arm. Outcome measures will then be completed in the following order: GHQ-12; DISCS; SWEMWBS; CPIB; CIQ; EQ-5D-5L; CCRSA; FS. Aphasia will then be fully assessed with the Western Aphasia Battery- Revised (WAB-R);[30] and cognition with the Cognitive Linguistic Quick Test (CLQT),[31] which

Purpose	Person responsible (Location)	Time	Forms/ Measures	Description
				has been specifically developed for people with aphasia. These assessments will be reported descriptively under participant characteristics.
RANDOMISATION (time 0)				
Consent (PEER arm only)	Trial Manager (Home)	30 minutes	Stage 2 Consent CRF; Intervention registration CRF	This visit will occur following the background and baseline assessments and randomisation. This visit will be conducted to provide information about what peer-befriending entails for those participants randomised to this arm of the trial and obtain consent (2nd stage consent).
PEER Befriender visits (PEER arm only)	Peer Befrienders (Home or community)	6-8 visits (each min.1 hr)		Patients in the PEER group only will receive six visits from a peer befriender over a 3-month period. A further two visits in the next 6 months for a gradual transition to the end of the intervention will also be offered.
Follow-up (4 months post randomisation)	Blind assessor (Home)	1-2 visits (each 1-1.5 hrs)	Personal & social case history CRF; GHQ-12; DISCS; SWEMWBS; CPIB; CIQ; EQ-5D-5L; CCRSA; FS; CSRI	This visit will occur at 4 months post-randomisation. Sections of the Personal & social case history CRF will be repeated to check for any changes, e.g. in marital status, work status. Outcome measures will be completed in an interview format with a blind assessor with expertise in facilitating people with aphasia. The number of sessions to complete these assessments will be dependent on a participant's aphasia severity and cognitive ability (e.g. level of concentration and fatigue).
	Qualitative Research assistant (Home)	1 visit (1 hour)		In addition a purposively selected sample of participants will take part in in-depth interviews (n=10 USUAL; n=10 PEER). A qualitative research assistant with expertise in qualitative methodology will conduct these interviews. The assistant will be aware of treatment allocation, and will work independently from the blind assessors conducting the quantitative assessments. The interviews will explore the impact of stroke and aphasia on the person's emotions; and what has helped getting on with life; confidence; feeling hopeful; and their perspectives on whether and how the care they received supported their well-being. Those who received peer befriending will also be asked about their experiences of the intervention, its perceived benefits and any difficulties. Participant perspectives on the study protocol (e.g. recruitment process, assessments) will also be explored.
	Trial Manager or qualitative research	1 visit (1 hour)		To collect information about resource use using the CSRI, to be completed with significant other as primary respondent where available and participant with aphasia

Purpose	Person responsible (Location)	Time	Forms/ Measures	Description
	assistant (Home)			(with additional information drawn from community rehabilitation teams and records).
Follow-up (10 months post randomisation)	Blind assessor (Home)	1-2 visits (each 1–1.5 hrs)	GHQ-12; DISCS; SWEMWBS; CPIB; CIQ; EQ-5D-5L; CCRSA; FS; CSRI	This session will occur at 10 months post-randomisation. Outcome measures will be completed in an interview format with a blind assessor with expertise in facilitating people with aphasia.
	Qualitative research assistant (Home)	1 visit (1 hour)		The research assistant working on the qualitative arm of the project will also visit independently at 10 months, and will re-interview the subset of PEER participants (n=10) a second time about the longer term impact of the stroke and aphasia and their perceptions about the intervention.
	Trial Manager or qualitative research assistant (Home)	1 visit (1 hour)		The Trial Manager or qualitative research assistant will conduct a visit to collect information about resource use using the CSRI (see above).

Assessments completed at each time point – People with aphasia

Purpose	Assessment	Baseline	4 months post-randomisation	10 months post-randomisation
Personal & social case history CRF	Demographic & other variables	X	X	X
Background assessments	WAB-R	X		
	CLQT	X		
Primary Outcome(s)	GHQ-12	X	X	X
	DISCS	X	X	X
Secondary Outcomes	SWEMWBS	X	X	X
	CPIB	X	X	X
	CIQ	X	X	X
Exploratory Outcomes	CCRSA	X	X	X
	FS	X	X	X
Economic Evaluation	EQ-5D-5L	X	X	X
	CSRI		X	X
Qualitative interviews			X<br>N=20	X<br>N=10

RANDOMISED CONTROLLED TRIAL – Significant Others

Consent for significant others will be sought on the consent visit. A set of baseline measures will be completed pre-randomisation and then again at 4 and 10 months. A sub-sample, purposively selected, of significant others (n=5 USUAL; n=5 PEER) will take part in a semi-structured interview at 4 months and cover: the impact of their partner’s stroke and aphasia on their life and family life; and their perspectives of the care received. The subsample in the PEER arm will also be asked about the administration and impact of peer befriending.

RANDOMISED CONTROLLED TRIAL – Significant others

Purpose	Person responsible (Location)	Time	Forms/ Measures	Description
Consent	CRN, SLT or Research team member (Hospital or home)	15-20 minutes	SO Registration & eligibility CRF; Stage 1 Consent CRF; SO Case history CRF	Each participant with aphasia will be asked to nominate a significant other to take part in the study. Significant others will be screened for eligibility with some key questions to ensure they meet inclusion and exclusion criteria. Information about the project and an information sheet will then be given to the significant other. Written consent will be obtained to participate in the study.
Baseline assessments	Blind assessor (Home)	1 session (about 1 hr)	SO Personal & social case history CRF; WEMWBS; GHQ-28; BCOS	These assessments will occur at approximately the same time that participants with aphasia are completing their background and baseline assessments. Significant others will complete a case history, covering: demographic and health information and family and social circumstances. This information will be used to report on their characteristics. The significant other will also complete baseline outcome measures. All measures will be completed in an interview format with a blind assessor.
RANDOMISATION (time 0)				
Follow-up (4 months post randomisation)	Blind assessor (Home)<br><br>Qualitative Research assistant (Home)<br><br>Trial Manager or qualitative research assistant (Home or by telephone)	1 visit (40 – 45 mins)<br><br>1 visit (1 hour)<br><br>1 visit (1 hour)	SO Personal & social case history CRF; WEMWBS; GHQ-28; BCOS; CSRI	This visit will occur at 4 months post-randomisation. Sections of the SO Personal & social case history CRF will be repeated to check for changes. Outcome measures will be completed in an interview format with a blind assessor.<br><br>A sub-sample, purposively selected, of significant others (n=5 USUAL; n=5 PEER) will take part in a semi-structured interview and cover: the impact of their partner's stroke and aphasia on their life and family life; and their perspectives of the care received. Those in the PEER arm will also be asked about the administration and impact of peer befriending<br><br>To collect information about person's with aphasia resource use using the CSRI, to be completed with significant other as primary respondent where available and participant with aphasia (with additional information drawn from community rehabilitation teams and records).

Purpose	Person responsible (Location)	Time	Forms/ Measures	Description
Follow-up<br>(10 months post randomisation)	Blind assessor<br>(Home)<br><br>Trial Manager or qualitative research assistant<br>(Home or by telephone)	1 visit<br>(40 – 45 mins)<br><br>1 visit (1 hour)	Home<br>WEMWBS;<br>GHQ-28;<br>BCOS; CSRI	This session will occur 10 months post-randomisation. Outcome measures will be completed in an interview format with a blind assessor.<br><br>The Trial Manager or qualitative research assistant will conduct a visit to collect information about resource use using the CSRI (see above)

Assessments completed at each time point – Significant others

Purpose	Assessment	Baseline	4 months post-randomisation	10 months post-randomisation
SO Personal & social case history CRF	Demographic, health information, family and social circumstances	X	X	X
Outcome measures	WEMWBS	X	X	X
	GHQ-28	X	X	X
	BCOS	X	X	X
Economic evaluation	CSRI		X	X
Qualitative interviews			X<br>N=10	

RANDOMISED CONTROLLED TRIAL – Peer Befrienders

After giving consent, peer befrienders will be DBS checked. They will attend a session where they complete a range of baseline measures. This session will be followed by 5-6 hours of peer befriender training over 2-3 days delivered by the befriender facilitator(s). Completion of baseline measures and training will happen whilst waiting for DBS clearance. Once DBS clearance is obtained, peer befrienders will be allocated people with aphasia who have been discharged from hospital and who have completed any intensive community therapy. They will visit people with aphasia at least six times over a 3-month period. During this time, peer befrienders will attend monthly group supervision sessions, which will provide them an opportunity to share experiences and discuss any difficulties that have arisen. Peer befrienders will then complete the GHQ-12 (to monitor for depression, see section 5.2) and outcome measures again once they have completed two befriending cycles, i.e. six befriending visits with each of two participants. For befrienders who do not fully complete a cycle because, e.g. the participant with aphasia dies or withdraws, the cycle will be considered completed if they have done a minimum of two visits. Any peer-befrienders who do not complete peer befriending with two participants will complete outcomes measures within 1-month prior to the completion of the study data collection. At the time of completing outcome measures, all peer befrienders will take part in semi-structured interviews to explore their experience of the study, including reflections on programme training and delivery, support received, any concerns or difficulties, and perceived benefits.

RANDOMISED CONTROLLED TRIAL – Peer befrienders

Purpose	Person responsible (Location)	Time	Forms/ Measures	Description
Screening and Consent	Trial Manager (Home)	1 hour	PB Registration & eligibility CRF; PB Stroke case history CRF; GHQ-12; FAST	Peer-befrienders will be screened with the GHQ-12 to ensure they are not depressed and the FAST to ensure their aphasia is mild-moderate. If eligible, initial information about the study will be given to each potential befriender which the Trial Manager will go through in detail. Written consent will then be obtained from the peer befriender to participate in the study.
Background & Baseline assessments	Blind assessor (Home)	1-2 sessions (each 1-1.5 hrs)	PB Personal & social case history CRF; CIQ; WEMWBS; GSE; GHQ-12	These assessments will occur within 1-2 weeks of giving consent. The number of sessions to complete the assessments will be dependent on a befrienders aphasia severity and cognitive ability (e.g. level of concentration and fatigue). All assessments and outcome measures will be completed in an interview format with an assessor with expertise in facilitating people with aphasia. At the first assessment point, all befrienders will complete a case history, covering: demographic and stroke-related variables and personal interests. This will be used as background information, and will also contribute to pairing with peer-befrienders for those in the intervention arm.
Training	Befriender facilitator (City, University of London)	1-day (1 day or two half days: to be determined in development phase)		This group training course will last 5-6 hours over 2-3 days to accommodate the needs of the befrienders in the group (i.e. levels of attention and fatigue). The training will provide befrienders with the skills and knowledge to work as a befriender, support and converse with people with aphasia who are a short time post-stroke, and help the person with aphasia adjust to life post-stroke. The befriender will attend the course with other peer befrienders.
PEER befriender visits	Peer Befrienders (Home or community setting)	6-8 visits (each 1-2 hrs)	PB visit record sheets	Each peer befriender will be matched and allocated to a minimum of 2 people with aphasia post-stroke. Each befriender will visit the person six times over a 3 month period with a further two visits in the next 6 months for a gradual transition to the end of the intervention. It is anticipated that each peer-befriender will work with 2-4 participants during the project and no more than two at any one time.

Purpose	Person responsible (Location)	Time	Forms/ Measures	Description
Peer befriender supervision	Befriender facilitator (City, University of London or community setting)	Monthly (1 – 1.5 hrs)	Befriender facilitator supervision records	Peer befrienders will be offered monthly group supervision sessions from a befriender facilitator. These will be an opportunity to share experiences and discuss any difficulties that have arisen. Peer-befrienders who are facing particular challenges e.g. with one participant, will receive supplementary individual supervision from their facilitator. Peer befrienders and the facilitators will record these challenges.
Follow-up	Blind assessor (Home)<br><br>Qualitative Research assistant (Home)	1 visit (1 hour)<br><br>1 visit (1 hour)	PB Personal & social case history CRF; CIQ; WEMWBS; GSE; GHQ-12	This visit will occur after a befriender has completed befriending two people with aphasia. If they are unable to befriend two people, this visit will occur within 1-month prior to the completion of the study data collection. Sections of the PB Personal & social case history CRF will be repeated to check for any changes. The outcome measures and GHQ-12 to monitor for depression will be completed in an interview format with an assessor with expertise in facilitating communication with people with aphasia. The number of sessions to complete these assessments will be dependent on a peer befrienders aphasia severity and cognitive ability (e.g. level of concentration and fatigue).<br><br>Befrienders will also take part in a semi-structured interview to explore their experience of the study, including reflections on programme training and delivery, support received, any concerns or difficulties, and perceived benefits.

Assessments completed at each time point – Peer Befrienders

Purpose	Assessment	Baseline	Following peer befriending
PB Personal & social case history form	Demographic, stroke-related variables and personal interests	X	X
Aphasia assessment	FAST	X	
Outcomes	CIQ	X	X
	WEMWBS	X	X
	GSE	X	X
Monitoring	GHQ-12	X	X
Qualitative interviews			X

2.6 Sample size

We will recruit in total 60 participants with aphasia (30 in each arm of the study). Allowing for a ~15% lost to follow up rate, at least 50 will complete the study. With 60 participants recruited, we will be able to estimate a 95% confidence interval for the recruitment rate to within approximately 25%. This sample size will be adequate to estimate important parameters needed to inform the design and the sample size of a full trial, such as the standard deviation, consent rates, event rates. This sample size also meets recommended sample sizes for feasibility studies.[32]

Although this is a feasibility study, we would like to estimate exploratory differences between the PEER and USUAL arms. If we assume we retain 50 participants at follow-up, a two-sided test, $\alpha = 0.05$, and independence of participants, we will have 80% power to detect an effect size of approximately 0.8. The standard deviation of the primary GHQ-12 outcome in a generic population of patients with stroke has been shown to be approximately 3.6 – this was using the 0 0 1 1 scoring, with total scores ranging from 0 – 12.[33] This effect size corresponds to an approximate mean difference of 3 points between groups. This sample size is sufficient for these exploratory comparisons, however, in a definitive trial we would want to have higher power to detect a smaller effect size. This feasibility study will give us a standard deviation specific to the group that we would be interested in recruiting for a definitive trial, so would help to inform such a sample size calculation.

2.7 Recruitment

Four NHS Trusts are involved, aiming to recruit one - two participants with aphasia from each Trust, each month. If recruitment is slower than anticipated in the first three months of recruitment an additional Trust will be added. Five main hospitals across four NHS Trusts are involved: the Homerton Hospital, the Royal London Hospital, and University College London Hospital, the National Hospital for Neurology and Neurosurgery and the Royal Free Hospital. Lead clinicians (SLTs) and CRNs will identify and screen all individuals with aphasia who are undergoing discharge. Standard operating procedures for screening and obtaining consent will be provided to the recruitment sites. Any necessary training for interacting with people with aphasia will be provided by the Trial Manager who is a highly specialist SLT. The numbers of people screened, identified as eligible, and consented will be recorded at each site and provided to the Trial Manager at the end of each month. Potential participants identified in hospital but discharged before screening will have their details forwarded to the Trial Manager with their consent and will be screened and consented into the study by a member of the research team (see also participant timeline in section 2.5). Participants identified in the community and/or people who are re-screened upon discharge from hospital into the community will be screened and/or recruited a CRN, community SLT or member of the research team. Significant others will be recruited at the same time as people with aphasia. However, in cases where this is not possible recruitment will occur during the initial home visit.

3. Methods: Assignment of interventions (for controlled trials)

3.1 Allocation

The King's Clinical Trials Unit (KCTU) will provide the randomisation service. Randomisation will use the web-based service hosted at the King's Clinical Trials Unit (KCTU) in accordance with a standard operating procedure and held on a secure server. Once eligibility has been confirmed, the informed consent form has been signed, and baseline assessments have been completed, the Trial Manager will access the KCTU randomisation system within 0-3 days to randomise the patient at the individual level (trial time point 0). A unique study patient identification number (PIN) will be assigned to each participant by the MACRO system. Each participant will be randomised 1:1 to PEER or USUAL care. The randomisation allocation will utilize minimization with a random component. Minimisation will be based on the following characteristics: severity of aphasia (based on WAB cut-offs), recruitment area (Hackney, Tower Hamlets, Camden & Islington) and physical ability (i.e. wheelchair-user or not). The study statisticians and investigators will be blinded to treatment allocation until data collection is complete. The investigators will remain blinded until the analyses are completed, with the statisticians remaining blinded for as long as practically possible during the analysis. The trial manager and qualitative and health economics researchers will be unblinded.

3.2 Blinding (masking)

In behavioural interventions, blinding participants to treatment versus control allocation is problematic. If participants are provided with information about the intervention to be tested, as ethics guidelines require, they will know whether they are in the intervention or the control arm of the study. This is particularly problematic in psychological interventions where people who may already be distressed or anxious are likely

to become even more distressed when they realise they are in the control arm of a study. Where a participant is aware that they have been allocated to the control condition, there are potentially threats to validity and maintaining lack of bias.[34,35] These threats include: the ‘resentful demoralisation’ effect[36] whereby when participants are allocated to their non-preferred arm of a trial it leads to deflated scores on psychological outcome measures, and/or non-compliance; selective differential attrition rates between groups; and lack of consent to randomisation from potential participants with strong preferences over group allocation.[34,35] Indeed, in our team’s previous experience with a peer support intervention with mental health patients at the time of discharge, many potential participants refused to take part unless they were guaranteed to receive the peer support.[37] In such circumstances, some advocate the use of a Zelen design where only those in the experimental group consent to the trial. An ethical concern with such a design is that participants are included in a study without their consent.

To minimise these threats, we are following a modified two stage consent design,[38,39] as highlighted in the MRC framework for complex interventions. This will ensure that those in the control group consent to take part in the study and have their data collected and compared to others, but are blind to group allocation. First, we will invite participants to join a study monitoring progression and adjustment to life post stroke and aphasia, and comparing different packages of care. Those who consent will be randomised. Those assigned to the control group will receive usual care; they will know that other people may receive different care, against which they will be compared, but they will not know what this entails. The second stage of consent will involve only those participants who have been randomised to peer befriending. A separate visit (conducted within their own home/ community setting) will be conducted by the Trial Manager within 1-2 weeks of randomisation to inform participants of their allocation in the PEER arm of the trial; give them information about the peer befriending and get their consent to participate in this arm.

The principal investigator, study statisticians, and research assistants collecting post-baseline data will be blinded to treatment allocation until data collection is complete. The Trial Manager, qualitative researchers, health economics researchers and the befriender supervisor will be unblinded.

To ensure blinding of quantitative outcome measures for the research assistants collecting post-baseline data, participants with aphasia who received peer befriending will be told to not reveal this information during assessment. To further reduce instances of unblinding particularly within the work environment, research assistants will not have access to participant files, computer documents with participant details will be password protected, they will not be permitted to answer the work telephone and all visits will be arranged by the Trial Manager.[40] If a research assistant becomes unblinded they will report this to the trial manager who will keep a record of such instances. If this happens during the 4 month assessment point, the 10 month assessments will be completed by another research assistant blind to group allocation.

The trial statisticians will remain blinded to group by utilising the partially unblinded facility for data extracts from the KCTU randomisation system, where the groups will be labelled in a non-discernable way, i.e. A and B. Data that are gathered and stored on the peer befrienders and on which peer befriender sees which participant will be held in a second database that is separate from the main database. The trial statisticians

will not request extracts from this database until such a time as they need to become unblinded to complete the primary analysis. Further information about blinding of other investigators and researchers is detailed in section 4.2. on data management.

No instances are expected to occur where a participant in the control arm may need to be unblinded.

4. Methods: Data collection, management, and analysis

4.1 Data collection methods

Outcome measures data will be collected by blind assessors who are skilled in communicating with people with aphasia. As part of the development phase, example assessment sessions will be videotaped between a member of the research team and consultants with aphasia (see appendix 1). These videos will be used to train each blind assessor in order to ensure consistent administration of the outcomes within assessment sessions. In addition, the Trial Manager will observe the first assessment session of each assessor. Further assessment sessions will be observed at random by the Trial Manager either in person or by reviewing videotaped sessions. Assessors will be given a data collection pack for each participant, at each time point. These packs will contain a script to guide their conversations with participants and verbal instructions for each of the outcomes administered.

The qualitative research assistant conducting the semi-structured interviews will similarly be supported. They will receive training in conducting semi-structured interviews from Dr Sarah Northcott (co-applicant), who has extensive experience of adapting qualitative methodologies for people with aphasia. Dr Northcott will listen to two initial interviews and give feedback, for example, to ensure questioning is unbiased and leads to full exploration of topics. Dr Northcott will continue to support the qualitative research assistant throughout the project, and will periodically listen to interviews.

For the economic evaluation, either the trial manager or the qualitative research assistant will collect the CSRI information. They will be given training in completion of the CSRI and eliciting resource use information by a member of the study team with relevant health economics expertise (Dr Chris Flood). As much information as possible will be collected from the significant other as primary respondent, where available. The Trial Manager will draw additional information by contacting CRN nurses or members of the clinical team as appropriate. Dr Flood will help to supervise the collection of data to ensure completeness.

To facilitate participants to attend baseline and follow-up sessions, participants, significant others and peer befrienders will be contacted by either text/telephone at least one week prior to each assessment session and on the previous day to confirm appointment times. To maximise participant engagement in the project and retention of participants, a quarterly newsletter will be sent to them throughout their involvement in the study, and a single phone-call between follow-up assessment times will also be made to remind them of their next appointment.

4.2 Data management

4.2.1 Trial databases

The main trial database will be hosted at King's Clinical Trials Unit (KCTU). Data will be managed using the InferMed MACRO database system. An electronic Case Report Form (eCRF) will be created using the InferMed MACRO system. This system is regulatory compliant (Good Clinical Practice, title 21 of the Code of Federal Regulations Part 11, European Commission Clinical Trial Directive). The eCRF will be created in collaboration with the trial statisticians and the CI and maintained by the King's Clinical Trials Unit until data is transferred to the CI at City. It will be hosted on a dedicated secure server within KCL. The Chief Investigator will act as custodian for the trial data. Source data will be entered by authorised staff (i.e. Trial Manager and research assistants) onto the eCRF with a full audit trial in which participants will be identified by their unique PIN. The main trial database will include feasibility data on people consented, with these data being aggregated where we do not have consent to hold participants' data (for example, reasons for declining participation), as well as assessment and outcome measures data, and Serious Adverse Event (SAE) data.

A separate database on intervention details will also be hosted at KCTU. This will include all necessary information on the intervention each participant in the PEER arm received as extracted by visit record forms and information from supervision sessions (e.g. number of sessions arranged, completed, missed & reason why; duration of session; activities performed and goals pursued). The intervention database will meet the same requirements as the main trial database above, but only the trial manager will be authorised to input data and have access. Trial statisticians will only have access after unblinding. Data in the second database will be extracted and summarised routinely by an unblinded statistician independent to the trial where necessary.

Fidelity data, qualitative and health economic data will be kept at City, University of London (City). The fidelity dataset will be anonymised with participants being identified by their PIN. The trial manager and unblinded researchers analysing fidelity data will have access to it. Qualitative data will be transcribed and then saved by the qualitative research assistant in an electronic database, on a secure network drive within the City system, which is regularly backed up. Participants in the qualitative data database will be identified by their unique PIN, however this database will also include identifying information, such as gender and age. Health economic data will also be entered on a secure City network drive, onto a password-protected Excel spreadsheet, by the trial manager only. Each participant on this spreadsheet will be identified by their PIN. Qualitative and health economics data will be accessible by the Trial Manager, qualitative and health economics researchers.

4.2.2 Database passwords

Database access will be strictly restricted through passwords to the authorised research team. The CI or delegate will request usernames and passwords from the KCTU. It is a legal requirement that passwords to the eCRF are not shared, and that only those authorised to access the system are allowed to do so. If new

staff members join the study, a personalised username and password should be requested via the CI or delegate (e.g Trial Manger) from the KCTU administrator.

Qualitative data will be stored in a password-protected drive at City. The trial manager and those involved with collecting, inputting and analysing qualitative research data (qualitative researcher(s), Dr Sarah Northcott) will be the only members of the research team with password access. The trial manager and those involved with collecting, inputting and analysing health economics data (health economics researcher, Dr Flood) will be the only members of the research team with password access to this Excel spreadsheet.

4.2.3 Data Handling & Confidentiality/Format of Records

We will adhere to NHS confidentiality practice, and to the Research Governance Framework in monitoring and managing the research. As CI, Prof Katerina Hilari will undertake overall responsibility for management of the project. The eCRF will be designed to promote accurate recording of data. A Data Management Plan will detail measures taken to promote data quality, for example, range and logic checks, and to monitor data completeness. Data will be handled, computerised and stored in accordance with the Data Protection Act, 1998. Participants will be identified on the trial and other study databases using their unique PIN. Access to data extracts from the main trial database will be confined to the trial manager and statisticians and will proceed via completion of KCTU data request forms Data Request Form (DM06 Form1) and R-004 (Randomisation Data Extract Request Form) v2.0 and submission of the forms to ctu@kcl.ac.uk. The trial manager and other research staff will maintain accurate participant records/results detailing observations on each participant enrolled. At the end of the study, essential documentation will be archived in accordance with requirements of City and destroyed 10 years after study completion. The retention of study data will be the responsibility of the Chief Investigator.

4.2.4 Identifiable and Unblinded Data

All participant contact/screening and recruitment data will be stored on spreadsheets at City, which will have restricted access from password-protected network drives. Hard copy of identifiable data (e.g. consent forms) and potentially identifiable data (screening sheets which will include participant initials) will be collated by the trial manager and stored in locked filing cabinets at City. Only the trial manager and the trial administrator will have access to this data. Identifiable data will not be entered on the eCRF, transferred to the KCTU or transferred to the trial statisticians. Data pertaining to the intervention that could unblind the statisticians will only be entered in the database specifically designed for data about the intervention. Intervention related data that could unblind the statisticians will not be entered in the main trial database, in particular, not in text fields that would be extracted and perused by the statisticians (for example on serious adverse event and withdrawal forms).

For qualitative data, the transcripts will clearly indicate the arm of the trial a participant has been randomised. For that reason, access to the transcripts will be restricted to the qualitative research sub-team as detailed above under 4.2.2. Similarly, the excel spreadsheet containing the health economic data will also clearly indicate the arm of the trial a participant has been randomised. For that reason, access to the document will also be restricted as detailed in 4.2.2 above.

4.2.5 On-Site/Central Monitoring

The Trial Manager will conduct on-site/central monitoring. The likely approach will be for the trial manager to do monthly checks on data completeness and certain range checks as outlined in the Data Management Plan. In addition, regular checks of a selected number of participant records will be done at three monthly intervals to check that data recording procedures are being followed consistently and accurately. The data will also be perused by the trial statistician when extracted for DMC reports and at the end of the study. Data queries will be raised by the trial statistician and sent to the trial manager for resolution. Any issues found in any of these checks will be resolved against the original records and corrected in the MACRO database where possible. The amount of missing data will be reported to the DMC at DMC meetings.

4.3 Statistical methods

4.3.1 General

Data analysis will be performed by the trial statistician under the supervision of the senior trial statistician at King's College London, using a password-protected computer in an office in a secure corridor. A comprehensive statistical analysis plan will be developed and agreed with the trial's oversight committees. All analyses will be performed on an intention to treat basis. Every effort will be made to reduce loss to follow up and to collect outcome data from participants who have withdrawn from treatment should they give permission to do so.

4.3.2 Descriptive statistics

Trial flow data will be reported as outlined by the CONSORT statement. The primary and secondary outcomes will be summarised using summary statistics, for the entire trial population and by trial arm, at each trial time point. The primary and main secondary outcome means and confidence intervals will be plotted over time. These summary statistics will help inform sample size estimation for the definitive trial.

4.3.3 Feasibility outcomes

We will calculate proportion who are eligible of those screened (number eligible / number screened); proportion who are eligible at first screen (number eligible / number screened at first screen) and second screen (number eligible / number screened at second screen); rate of eligibility per month; proportion who consent of those eligible (number who consent / number eligible); rate of consent per month; and rate of recruitment (participants randomised) per month. The frequency and proportion of people consented who withdraw overall, by study arm, and by those who do before and after randomisation will be presented. This will specifically include describing those in the PEER arm who decline consent at the second stage. Appropriate 95% confidence intervals will be constructed for all of the above measures.

Qualitative data analysis will be used to evaluate the acceptability of research procedures, outcome measures and interventions to participants, their significant others and peer support workers (see section 4.3.9 below). Moreover, we will informally ask CRNs and other members of the clinical teams involved with

SUPERB in all our sites about their experiences with the trial. This information will provide additional context to our feasibility outcomes.

We will use descriptive statistics to document what usual care consists of, based on data from the CSRI. Fidelity of the peer befriending training and supervision to the training manual and the peer befriending intervention to the peer befriending handbook will be evaluated by calculating a per cent fidelity score (components implemented / components planned x 100). This will involve checking videotaped sessions (training, supervision & intervention) against fidelity checklists (developed during the development phase: see appendix 3). Inter- (two raters) and intra-rater reliability will be calculated for all three session types. Inter-rater agreement coefficients will be used to calculate inter and intra-rater reliability. Counts of sessions completed will indicate whether befriender/befriendee pairs have adhered to the six befriending visits.

4.3.4 Primary outcome analysis

The comparison will be between the PEER and USUAL groups. The GHQ-12 will be scored 0 0 1 1 and summed, resulting in a 0-12 score range. This overall total GHQ-12 score will be analysed using linear mixed models with the 4 and 10 months post-randomisation measures as dependent variables, with a random intercept for individuals, and time, the baseline GHQ-12 score, a dummy variable indicating treatment group, the minimisation stratification factors and any baseline variables that were imbalanced or that predicted missingness as independent variables. A treatment group by time interaction term will be included to allow for extracting comparisons at the 4 and 10-month post-randomisation time points. Participants who do not contribute any outcome measurements of the primary outcome at either time point will be omitted from this analysis. Participants who provide any outcome data will be included. These models will account for missing data using the maximum likelihood algorithm. Given that the sample size is relatively small, in addition to the minimisation factors, we will include as covariates any baseline variables that appear to be imbalanced. This should also improve the plausibility of the missing at random assumption, see Section 4.3.5. Modelling assumptions will be checked (e.g. normally distributed residuals). We do not plan to perform imputation as we expect complete baseline data and so would only potentially have missing outcome data – imputation is not necessary in this situation as it adds variability without any further benefit.

In addition, we will explore the possibility of calculating the GHQ-12 intraclass correlation coefficient (ICC) for clusters of participants seen by the same peer befriender, if at least some peer befrienders see multiple participants (e.g. >2). This ICC will help inform a sample size calculation for a larger trial and provide estimates of a peer befriender ICC to publish in the literature. Using the data from the PEER group, we will fit a linear mixed effects model with the 4 and 10 months post-randomisation measures as dependent variables and a random intercept for befriender to calculate the within-peer, between-peer and total variability, enabling us to calculate the ICC.

If the DISCS is used as the main primary outcome instead of GHQ-12, it will be analysed in a similar manner.

4.3.5 Secondary and exploratory outcome analysis

The participant and significant other outcomes will be analysed in a simliar manner to that described for the primary outcome, comparing the outcomes between the PEER and USUAL groups. The peer befriender outcomes will be compared between the baseline and post-befriending time points using a paired t-test.

4.3.6 Missing data

The amount and reasons for missing data will be summarised overall and by treatment group. Missing data will be accounted for where mixed models are applied under the missing at random assumption using the maximum likelihood algorithm. The baseline characteristics of those missing follow-up and those with complete follow-up will be summarised and variables affecting missingness will be examined using a logistic predictor of missingness model. This will be done by generating a binary variable for missingness for the primary outcome variable 10 months post-randomisation (we will explore the need to do this either separately or combined for the primary and secondary outcomes) and regressing this on baseline variables. Any variables found to be important predictors of missingness will be included in the primary and secondary outcome models. From a health economic perspective, where there is missing data we will analyse the data on a complete case analysis basis and additionally use mean imputation for missing values. This will allow for a sensitivity analysis indicating whether missing values could be biasing the data.

4.3.7 Safety outcomes

Please see section 5.2 on Harms for definitions of the safety outcomes. Adverse events, adverse reactions, serious adverse events, serious adverse reactions and suspected unexpected serious adverse reactions will be summarised overall and by treatment group as counts of events and counts of people who have had events. We will calculate differences in event rates between the two treatment groups and a 95% confidence interval for the differences.

4.3.8 Per protocol analysis

Another analysis of the primary and secondary participant outcomes will be done as described above, excluding partcipants who are not in compliance with the protocol. Participants deemed not compliant will be as follows:

- Participants in the peer befriending arm who do not consent at the second stage
- Completing less than six peer befriending sessions
- Being found ineligible after randomisation based on the inclusion/exclusion criteria
- Participants in the USUAL group who get peer befrending outside the study, as reported to our outcome assessors or measured using the CSRI (in addition to their removal, we may explore analysing them in the PEER group).

4.3.9 Qualitative data

Qualitative data will be reported according to the Standards for Reporting Qualitative Research guidelines (SRQR). All interviews will be transcribed verbatim. Data will be analysed using Framework Analysis.[41] Initial themes and concepts will be identified through reviewing the data. These will then be used to construct a thematic index. All the material will then be indexed, so that each phrase or passage can be assigned a label. Thematic charts will be constructed, the chart headings evolving from the indexing process. The labelled raw data will then be summarised and synthesised into the matrices. This matrix based method of analysis enables systematic exploration of the range of views, and easy comparison both between cases and within cases to produce both descriptive and explanatory accounts of the data. In order to minimise potential bias a second analyst will independently index a proprotion of transcripts and analyse the matrix-based material, to ensure all relevant thematic material is represented fairly and is included in the final framework.

4.3.10 Economic evaluation

This pilot economic evaluation study has the objective of detecting problems in the cost collection, exploring how patients respond to the primary outcome in the study, the GHQ-12, and comparing it to the EQ-5D-5L instrument, as well as developing the economic evaluation model that will be applied to a phase III RCT. The pilot economic evaluation will compare incremental costs to incremental health gains of the PEER arm versus USUAL arm. Due to the very low numbers, this will be strictly an exercise to detect problems in the process of data collection and development of the full economic evaluation model. The rationale is to use the feasibility stage data to prepare a future full trial cost-effectiveness.

For the costs, we will use data collected on the Client Service Receipt Inventory (CSRI) on service use (including health, social and voluntary services) and associated costs at 4 and 10 months post-randomisation. Unit costs of resources used will be derived from routine sources locally where possible, and from national sources such as the NHS reference costs.

Health gains will be obtained from the answers to both the GHQ-12 and to the EQ-5D-5L instruments. The gold standard for economic evaluation is to use generic health state outcome measurements because these allow comparability across clinical areas. Thus, we will run two types of pilot economic evaluation analyses. First, we will run a pilot cost-effectiveness analysis based on the GHQ-12. Second, we will perform a pilot cost-utility analysis using Quality Adjusted Life Years (QALYs) gained based on the answers to the EQ-5D-5L. Additionally, since there is limited evidence on how the GHQ-12 and EQ-5D-5L relate to each other,[42] we will use the pilot data to explore the correlation of these two instruments in terms of health gains in different domains.

With the pilot cost and outcome data, we will explore the calculation of confidence intervals for costs and health gains using non-parametric bootstrapping, with the understanding that low numbers preclude generalisability. Also, we will explore the application of probabilistic sensitivity analysis that will eventually

generate cost-effectiveness acceptability curves in a Phase III RCT. These curves graphically represent provisional estimates of the cost-effectiveness of the intervention.

5. Methods: Monitoring

5.1 Data monitoring

The TMC will monitor the on-going day-to-day collection of data on a monthly basis. Data will also be monitored by the DMC who will meet four times to approve the data management plan, monitor adverse events and monitor the progress of the trial in relation to the primary endpoint; and once on completion of data collection.

5.1.1 Stopping guidelines

There are no formal statistical criteria for stopping the trial early. Decisions to stop the trial early on grounds of safety or futility (with regard to recruitment) will be made by the TSC on the basis of advice from the Data Management Committee.

5.2 Harms

Adverse events (AE) are any clinical change, disease or disorder experienced by the participant during their participation in the trial, whether or not considered related to the use of treatments studied in the trial. Adverse events associated with the peer befriending intervention are considered unlikely to participants and low risk. Such adverse events may include:

- Participants get upset during assessment and outcome measurement, or peer befriending visits (e.g. tension between assessors or befrienders, and participants).
- Peer befrienders complaining of deteriorating mood or scoring above the GHQ-12 cut-off on follow up visits (3 or more out of 12)

Adverse events not related to the intervention may include:

- Participants reveal to assessors unrelated new medical issues, which require an assessment by a healthcare professional or GP (e.g. fits or seizures, worsening visual difficulties, increased frequency or severity of headaches, accidents or injuries such as falls).
- New medical diagnosis of depression or signs of depression that raise clinical concern. (e.g. participants indicating that they are feeling low, unwell or different from usual).
- Risks arising in the home or within the family (e.g. unkempt house, falls)

If these adverse events do not lead to any of the outcomes listed a-f below, they will be considered non-serious. Their reporting is covered in the next section. Research assistants will be trained on how to respond to such events (e.g. advise participant to talk to their GP about medical and mental health concerns). Issues around peer-befriending will be discussed in supervision with the befriender facilitator. With peer-befrienders who score on the high emotional distress range on the GHQ-12 on their follow up visit, the trial manager with

explore with them whether they find peer-befriending distressing and whether they need to be withdrawn from the study (see also section 6.8 Ancillary and Post-trial care).

Serious adverse events (SAEs) are considered unlikely in this project, however, they may still occur. An AE is considered a SAE if it results in one of the following outcomes:

a) Death
b) Life-threatening (i.e., with an immediate, not hypothetical, risk of death at the time of the event, e.g., further stroke, cardiovascular event, serious infection)
c) Requires hospitalisation or prolongation of existing hospitalisation (hospitalisation for elective treatment of a pre-existing condition is not included)
d) Increased severe and persistent disability, defined as:
    - severe = a significant deterioration in the participant’s ability to carry out their important activities of daily living; and
    - persistent = 4 weeks continuous duration
e) Any other important medical condition, which, though not included in the above, may jeopardise the participant and may require medical or surgical intervention to prevent one of the outcomes listed.
f) Any episode of deliberate self-harm

A SAE is considered a serious adverse reaction (SAR) if the event is considered related to peer befriending (experienced either by the peer befriender or the person being befriended) or to the assessment or interview sessions with the research assistants. A SAE is considered a suspected unexpected serious adverse reaction (SUSAR) if the event is deemed as an unexpected reaction to peer befriending or interview or assessment sessions. Both SARs and SUSARs are also considered highly unlikely in this study. If there is any doubt as to whether the AE is a serious AE, a second opinion should be obtained from the trial manager and chief investigator.

5.2.1 Reporting AEs, SAEs, SARs and SUSARs

AEs (including SAEs, SARs and SUSARs) may be identified by research assistants (at each assessment point at baseline, 4 and 10 month post-randomisation); peer befrienders (during befriending visits); the befriender facilitator (in communication with peer befrienders); or the trial manager (on the consent visit post-randomisation). All research staff (i.e. research assistants, befriender facilitator, trial manager) and peer befrienders will be responsible for the reporting of AEs (including SAEs, SARs and SUSARs) from the time of the first baseline visit by the research assistant to the final follow up visit at 10 months randomisation.

All adverse events (including SAEs, SARs and SUSARs) will be recorded on forms (see Appendix 4) and scanned and emailed or faxed to the trial manager within 24-48 hours of occurrence. The SAE form includes the potential outcomes (a-f above) so we do not anticipate reporters to have difficulty choosing between the AE and SAE forms. Peer befrienders who have difficulty recording the event on the form will contact their

befriender facilitator who will assist in filling in the form and forwarding to the trial manager. These forms will be completed as thoroughly as possible with all available details of the event. If the research assistant or befriender facilitator does not have all information regarding the event, he/she will not wait to receive additional information before notifying the trial manager of the event and completing the form. In the case of incomplete information at the time of initial reporting, all appropriate information should be provided as soon as this becomes available.

In terms of SAE, the relationship of the event to the treatment (SAE or SAR) and expectedness of the event (SAR or SUSAR) may be assessed and indicated on the form by the research assistant, peer befriender or befriender facilitator. If uncertain, the trial manager will judge relatedness to treatment (SAE or SAR) and whether it was expected or not (SAR or SUSAR). If the trial manager is uncertain, they will consult with the chief investigator. The trial manager will immediately notify the chief investigator of any SAEs, SARs or SUSARs. SAEs will be transcribed to eCRF.

All AEs (non-serious) will be reported to the chief investigator on a monthly basis by the trial manager. These events will be reported to the DMC on an annual basis (or more frequently if requested) and will be included in the safety reporting of the completed trial.

All SAEs (including SARs and SUSARs) will be reported to the chief investigator and DMC immediately by the trial manager within 24-48 hours. A record of this notification (including date of notification) must be clearly documented to provide an audit trail. The chief investigator will inform the sponsor. If the SAE is considered by the chief investigator to be a SUSAR (i.e. related and unexpected), it will be reported to the Research Ethics Committee (REC) which approved the study, as per Health Research Authority (HRA) guidelines, using a modified version of the HRA non-CTIMP SAE report form (Appendix 3).

Reporting timelines are as follows:

- SUSARs which are fatal or life-threatening must be reported not later than 7 days after the sponsor is first aware of the reaction. Any additional relevant information must be reported within a further 8 days.
- SUSARs that are not fatal or life-threatening must be reported within 15 days of the sponsor first becoming aware of the reaction.

The Chief Investigator will provide an annual report of all SARs and SUSARs to the sponsor, the DMC and the Funder.

5.2.2 Follow-up after adverse events

After an SAE or SAR (including SUSAR), a decision will be made by the trial manager, in consultation with the chief investigator and where appropriate after advice from the relevant authorities and the participant's general practitioner, as to whether the participant should be withdrawn from either their randomised

treatment or from the trial. The trial manager will make arrangements for further assessment and management as agreed with the relevant authorities, GP and participant.

5.3 Auditing

Several mechanisms are in place to monitor and audit trial conduct. The Trial Management Committee (TMC) will meet monthly. At these meetings, it is the responsibility of the trial manager and the TMC to discuss trial progress and highlight any issues requiring further input. A sub-committee will meet on a weekly to fortnightly basis to monitor the trial and discuss issues that may arise e.g. recruitment, peer befriending visits and assessment sessions. The Data Monitoring Committee (DMC) will meet four times over the duration of the study and will report on study progress to the Trial Steering Committee. The TSC will meet six times during the project to oversee the study management. Each of these committees is outlined at the beginning of the protocol under “Trial Committees”.

6. Ethics and dissemination

6.1 Research ethics approval

The trial will be conducted in compliance with the principles of the Declaration of Helsinki (1996), the principles of Good Clinical Practice (GCP) and in accordance with all applicable regulatory requirements including but not limited to the Research Governance Framework and the Mental Capacity Act 2005. This protocol and related documents will be submitted for review to the Health Research Authority (HRA) through the Integrated Research Application System (IRAS). An application for Clinical Research Network (CRN) Adoption has already been submitted and accepted. The Chief Investigator will submit a final report at conclusion of the trial to the HRA, the Sponsor and Funder.

6.2 Protocol amendments

Any significant amendments to the protocol will be communicated with all relevant parties including the TSC, DMC, the sponsor, the funder, HRA and any trial registries that the study is registered with.

6.3 Consent or assent

Informed consent will be obtained from all participants. To ensure that each participant fully understands the nature of the study, we developed participant information materials and consent forms that are accessible to people with aphasia (see Appendix 5 for an example consent form). These materials were developed based on the experience of the applicants and in collaboration with our consultants with aphasia. The materials were developed following standard aphasia-friendly principles, such as presenting one idea at a time, using short simple sentences presented in large font, emboldening key words and representing key ideas with a suitable pictorial image. During the development phase we used templates created by the NIHR CRN for enabling people with aphasia to participate in research[43] and principles from the consent support tool, which has been specifically designed to facilitate the consent process with people who have aphasia.[44] Additionally, a group of six consultants with aphasia advised and reviewed the forms to ensure that they provided the essential information in the most appropriate way. To further facilitate the consent process, each participant

will have time (up to 48 hours) after information is provided to make an informed decision about whether they would like to consent to inclusion in the study. Any questions or queries they may have about the study will be discussed with the person obtaining consent. Scripts will be provided.

The person obtaining consent from participants will have experience of or receive training on communicating with people with aphasia using a total communication approach (e.g. explaining in simple short sentences, using gestures, pointing to pictures) and obtaining informed consent. Informed consent will be obtained for each participant (i.e. people with aphasia, significant other) by either the SLT at each recruitment site, the CRN or member of the research team. Informed consent for peer befrienders will be obtained by the trial manager. Second stage consent for those in the PEER arm of the study will be obtained by the trial manager who has extensive experience communicating with adults with acquired communication disorders and consent processes.

After information giving, the person obtaining consent will ask potential participants with aphasia three simple yes/no or forced alternative questions to check their understanding of key aspects of the study (Is this study about a drug or how you feel? Will our researchers visit you once or many times? Can you stop if you wish, yes or no?). If participants with aphasia cannot answer these correctly, this will suggest that they have such severe aphasia that they are unable to give informed consent and they will not be included in the study.

For participants who are physically unable to sign the form (e.g. due to weakness in dominant hand due to stroke) then consent will be given using a mark or line in the presence of an independent witness (who has no involvement in the trial) who will then corroborate by signing the consent form.

6.4 Progressing to a definitive trial

Effect sizes with 95% confidence intervals will be estimated to check that the likely effect is within a clinically relevant range as confirmation that it is worth planning a definitive trial. This information together with acceptability of the study data; safety of intervention; participant recruitment and retention rates will help us determine whether the definitive RCT is feasible.

6.5 Confidentiality

The Chief Investigator will act as custodian for the trial data. Management of the data will be kept secure, anonymised and confidential (see section 4.2) and in accordance with the 1998 Data Protection Act.

6.6 Declarations of interests

The chief investigator and co-investigators have no competing interests to declare for the overall trial and each study site.

6.7 Access to data

Data management and access was comprehensively described in section 4.2. In short, data will be held at Kings Clinical Trial Unit (KCTU) (main database, intervention database) and City, University of London (qualitative data, economic evaluation data, hard copy data). Dr Kimberley Goldsmith and Kirsty James (Junior Statistician) will have monitor-only access to the KCTU main database, and will only be able to access the data via requests to the KCTU for data extracts. Hard copy data that contains identifiable information will be secured in a filing cabinet accessible to only Dr Nicholas Behn and the research administrator. The qualitative data will be contained in password-protected documents on a secure network drive accessible by the qualitative research sub-team. The health economic data will be contained in a password-protected excel spreadsheet on a secure network drive accessible by the health economics research sub-team. Other research assistants and investigators on the trial who are not involved in the qualitative and health economics data collection and analysis will not have access to the electronic or paper records of this data.

6.8 Ancillary and post-trial care

During the trial, if a participant or peer befriender scores within the high emotional distress range of the GHQ-12 (3 or more)[44] or the GHQ-28 for significant others (6 or more)[73], the research assistant or trial manager will discuss with them their score and they will be asked if they feel their mood is low. They will be asked to consider talking to their GP and, if they would like, they will be given information on local support organisations and groups. For peer befrienders, the trial manager will explore with them whether they find peer befriending distressing and whether they need to be withdrawn from the study. Information about local support sources will also be offered if participants express feelings of loneliness and social isolation. On completion of the study all participants will receive information about local services and voluntary organisations like Dyscover, Speakability and the Stroke Project, which all support well-being post-stroke.

6.9 Dissemination policy

It is intended that the results of the study will be reported and disseminated at international conferences and in peer-reviewed scientific journals. These include the publication of the research protocol and publication of the results in an open access journal and the quarterly Bulletin (official magazine of the Royal College of Speech and Language Therapists). Our User Group will advise us regarding dissemination to the stroke community, which we anticipate will include publication of the results in stroke and aphasia voluntary organisations newsletters (e.g. The Stroke Association, Speakability). A results leaflet will be created and a dissemination event held to further explain the results to participants and local NHS therapists.

7. Insurance / Indemnity

City University has Professional Indemnity and Clinical Trials insurance cover for its liability relating to all of these activities. Professional Indemnity is for £15 million in any one occurrence and insured with Zurich. Insurance for clinical trials is for £10 million in any one occurrence and insured through Arthur J. Gallagher.

8. Financial Aspects

Funding provided by:

Stroke Association

Stroke Association House

240 City Road

London, EC1V 2 PR

Tel: 0207 566 0300

Amount: £490,664

9. Signatures

4th July 2018

Chief Investigator Date

Print name

KATERINA HILARI

4th July 2018

Statistician (if applicable) Date

Print name

KIMBERLEY GOLDSMITH

10. References

1. Hackett ML, Pickles K. Part I: frequency of depression after stroke: an updated systematic review and meta-analysis of observational studies. *Int J Stroke.* 2014;9(8):1017-1025.
2. Ayerbe L, Ayis S, Wolfe CD, Rudd AG. Natural history, predictors and outcomes of depression after stroke: systematic review and meta-analysis. *Br J Psychiatry.* 2013;202(1):14-21.
3. Pohjasvaara T, Vataja R, Leppavuori A, Kaste M, Erkinjuntti T. Depression is an independent predictor of poor long-term functional outcome post-stroke. *Eur J Neurol.* 2001;8(4):315-319.
4. Ghose SS, Williams LS, Swindle RW. Depression and other mental health diagnoses after stroke increase inpatient and outpatient medical utilization three years poststroke. *Med Care.* 2005;43(12):1259-1264.
5. Stroke Association. Feeling overwhelmed. 2013; https://www.stroke.org.uk/sites/default/files/feeling_overwhelmed_final_web.pdf.
6. Kauhanen M, Korpelainen JT, Hiltunen P, et al. Poststroke depression correlates with cognitive impairment and neurological deficits. *Stroke.* 1999;30(9):1875-1880.
7. Winkler M, Bedford V, Northcott S, Hilari K. Aphasia blog talk: How does stroke and aphasia affect the carer and their relationship with the person with aphasia? *Aphasiology.* 2014;28(11):1301-1319.
8. Cruice M, Worrall L, Hickson L. Quantifying aphasic people's social lives in the context of non-aphasic peers. *Aphasiology.* 2006;20(12):1210-1225.
9. Fotiadou D, Northcott S, Chatzidaki A, Hilari K. Aphasia blog talk: How does stroke and aphasia affect a person's social relationships? *Aphasiology.* 2014;28(11):1281-1300.
10. Northcott S, Hilari K. Why do people lose their friends after a stroke? *International Journal of Language & Communication Disorders.* 2011;46(5):524-534.
11. Tsouna-Hadjis E, Vemmos KN, Zakopoulos N, Stamatelopoulos S. First-stroke recovery process: the role of family social support. *Archives Physical Medicine & Rehabilitation.* 2000;81(7):881-887.
12. Boden-Albala B, Litwak E, Elkind MS, Rundek T, Sacco RL. Social isolation and outcomes post stroke. *Neurology.* 2005;64(11):1888-1892.
13. Lincoln NB, Worthington E, Mannix K, Lincoln N. A Survey of the Management of Mood Problems after Stroke by Clinical Psychologists. Paper presented at: Clinical Psychology Forum2012.
14. Intercollegiate Stroke Working Party. *National clinical guideline for stroke.* London: Royal College of Physicians;2016.
15. NHS Improvement - Stroke. *Psychological care after stroke: Improving stroke services for people with cognitive and mood disorders.* 2011.
16. Richards DA, Bower P, Pagel C, et al. Delivering stepped care: an analysis of implementation in routine practice. *Implement Sci.* 2012;7:3.
17. Hackett ML, Anderson CS, House A, Xia J. Interventions for treating depression after stroke. *Cochrane Database Syst Rev.* 2008(4):CD003437.
18. Thomas SA, Walker MF, Macniven JA, Haworth H, Lincoln NB. Communication and Low Mood (CALM): a randomized controlled trial of behavioural therapy for stroke patients with aphasia. *Clinical rehabilitation.* 2013;27(5):398-408.
19. Repper J, Carter T. A review of the literature on peer support in mental health services. *J Ment Health.* 2011;20(4):392-411.
20. Hoey LM, Ieropoli SC, White VM, Jefford M. Systematic review of peer-support programs for people with cancer. *Patient Educ Couns.* 2008;70(3):315-337.
21. Solomon P. Peer support/peer provided services underlying processes, benefits, and critical ingredients. *Psychiatr Rehabil J.* 2004;27(4):392-401.
22. Mead S, Hilton D, Curtis L. Peer support: a theoretical perspective. *Psychiatric rehabilitation journal.* 2001;25(2):134.
23. Mead N, Lester H, Chew-Graham C, Gask L, Bower P. Effects of befriending on depressive symptoms and distress: systematic review and meta-analysis. *Br J Psychiatry.* 2010;196(2):96-101.
24. Haun J, Rittman M, Sberna M. The continuum of connectedness and social isolation during post stroke recovery. *J Aging Stud.* 2008;22(1):54-64.
25. Enderby P, Wood V, Wade D. *Frenchay aphasia screening test (FAST).* West Sussex: John Wiley & Sons Ltd; 1986.

26. Turner-Stokes L, Kalmus M, Hirani D, Clegg F. The Depression Intensity Scale Circles (DISCs): a first evaluation of a simple assessment tool for depression in the context of brain injury. *Journal of Neurology, Neurosurgery & Psychiatry.* 2005;76(9):1273-1278.
27. Kneebone I, Baker J, O'Malley H. Screening for depression after stroke: developing protocols for the occupational therapist. *The British Journal of Occupational Therapy.* 2010;73(2):71-76.
28. McVicker S, Swinburn K. *How to set up a befriending Scheme: a guide for scheme organisers.* UK: Connect Press; 2009.
29. Rose TA, Worrall LE, Hickson LM, Hoffmann TC. Aphasia friendly written health information: content and design characteristics. *Int J Speech Lang Pathol.* 2011;13(4):335-347.
30. Kertesz A. *Western Aphasia Battery - Revised (WAB-R).* 2006.
31. Helm-Estabrooks N. *Cognitive Linguistic Quick Test (CLQT).* 2001.
32. Sim J, Lewis M. The size of a pilot study for a clinical trial should be calculated in relation to considerations of precision and efficiency. *J Clin Epidemiol.* 2012;65(3):301-308.
33. Hilari K. The impact of stroke: are people with aphasia different to those without? *Disabil Rehabil.* 2011;33(3):211-218.
34. Mohr DC, Spring B, Freedland KE, et al. The selection and design of control conditions for randomized controlled trials of psychological interventions. *Psychotherapy and Psychosomatics.* 2009;78:275-284.
35. Ainsworth HR, Torgerson DJ, Kang'ombe AR. Conceptual, design, and statistical complications associated with participant preference. *The Annals of the American Academy of Political and Social Science.* 2010;628:176-188.
36. Shadish WR, Cook TD, Campbell DT. *Experimental and quasi-experimental designs for generalised causal inference.* Boston: Houghton Mifflin; 2002.
37. Simpson A, Flood C, Rowe J, et al. Results of a pilot randomised controlled trial to measure the clinical and cost effectiveness of peer support in increasing hope and quality of life in mental health patients discharged from hospital in the UK. *BMC Psychiatry.* 2014;14:30.
38. Campbell R, Peters T, Grant C, Quilty B, Dieppe P. Adapting the randomized consent (Zelen) design for trials of behavioural interventions for chronic disease: feasibility study. *J Health Serv Res Policy.* 2005;10(4):220-225.
39. Torgerson DJ, Roland M. What is Zelen's design? *BMJ.* 1998;316(7131):606.
40. Minns Lowe CJ, Wilson MS, Sackley CM, Barker KL. Blind outcome assessment: the development and use of procedures to maintain and describe blinding in a pragmatic physiotherapy rehabilitation trial. *Clin Rehabil.* 2011;25(3):264-274.
41. Ritchie J, Spencer L. Qualitative data analysis for applied policy research. In: Bryman A, Burgess R, eds. *Analysing qualitative data*. London: Routledge; 1994.
42. Peasgood T, Brazier J, Mukuria C, Rowen D. A conceptual comparison of well-being measures used in the UK. 2014.
43. National Institute for Health Research. Enabling people with aphasia to participate in research: resources for stroke researchers. 2016; https://www.nihr.ac.uk/nihr-in-your-area/stroke/aphasia.htm.
44. Jayes M, Palmer R. Initial evaluation of the Consent Support Tool: a structured procedure to facilitate the inclusion and engagement of people with aphasia in the informed consent process. *Int J Speech Lang Pathol.* 2014;16(2):159-168.
45. Goldberg DP, Williams P. *A User's Guide to the General Health Questionnaire (GHQ).* Oxford: NFER-Nelson; 1988.
46. Ebrahim S, Barer D, Nouri F. Use of the Nottingham Health Profile with patients after a stroke. *Journal of Epidemiology and Community Health.* 1986;40:166-169.
47. Dennis M, O'Rourke,S., Slattery,J., Staniforth,T., & Warlow,C. Evaluation of a stroke family care worker: Results of a randomised controlled trial. *British Medical Journal.* 1997;314(1071).
48. Dennis M, O'Rourke S, Lewis S, Sharpe M, Warlow C. Emotional outcomes after stroke: Factors associated with poor outcome. *Journal of Neurology, Neurosurgery and Psychiatry.* 2000;68:47-52.
49. Hilari K, Byng S, Lamping DL, Smith SC. Stroke and Aphasia Quality of Life Scale-39 (SAQOL-39): evaluation of acceptability, reliability, and validity. *Stroke.* 2003;34(8):1944-1950.
50. Hilari K, Lamping DL, Smith SC, Northcott S, Lamb A, Marshall J. Psychometric properties of the Stroke and Aphasia Quality of Life Scale (SAQOL-39) in a generic stroke population. *Clin Rehabil.* 2009;23(6):544-557.

51. McDowell I. *Measuring health: a guide to rating scales and questionnaires.* Oxford university press; 2006.

52. Vieweg BW, Hedlund JL. The General Health Questionnaire (GHQ): A comprehensive review. *Journal of Operational Psychiatry.* 1983.

53. NHS Health Scotland, University of Warwick, University of Edinburgh. Short Warwick Edinburgh Mental Well Being Scale. 2007.

54. NHS Health Scotland, University of Warwick, University of Edinburgh. *Warwick Edinburgh Mental Well Being Scale: User guide version 2.* 2015.

55. Baylor C, Yorkston K, Eadie T, Kim J, Chung H, Amtmann D. The Communicative Participation Item Bank (CPIB): Item bank calibration and development of a disorder-generic short form. *Journal of Speech, Language, and Hearing Research.* 2013;56(4):1190-1208.

56. Baylor C, Oelke M, Bamer A, et al. Validating the Communicative Participation Item Bank (CPIB) for use with people with aphasia: an analysis of differential item function (DIF). *Aphasiology.* 2016:1-18.

57. Willer B, Ottenbacher KJ, Coad ML. The community integration questionnaire. A comparative examination. *Am J Phys Med Rehabil.* 1994;73(2):103-111.

58. Willer B, Rosenthal M, Kreutzer JS, Gordon W, Rempel R. Assessment of the community integration following rehabilitation for traumatic brain injury. *Journal of Head Trauma Rehabilitation.* 1993;8(2):75-87.

59. Kuipers P, Kendall M, Fleming J, Tate R. Comparison of the Sydney Psychosocial Reintegration Scale (SPRS) with the Community Integration Questionnaire (CIQ): psychometric properties. *Brain Inj.* 2004;18(2):161-177.

60. Corrigan JD, Deming R. Psychometric characteristics of the Community Integration Questionnaire: Replication and extension. *The Journal of Head Trauma Rehabilitation.* 1995;10(4):41-53.

61. Dalemans RJ, de Witte LP, Beurskens AJ, van den Heuvel WJ, Wade DT. Psychometric properties of the community integration questionnaire adjusted for people with aphasia. *Arch Phys Med Rehabil.* 2010;91(3):395-399.

62. Cherney LR, Babbitt EM, Semik P, Heinemann AW. Psychometric properties of the communication confidence rating scale for aphasia (CCRSA): Phase 1. *Topics in stroke rehabilitation.* 2015.

63. Babbitt EM, Heinemann AW, Semik P, Cherney LR. Psychometric properties of the communication confidence rating scale for aphasia (CCRSA): Phase 2. *Aphasiology.* 2011;25(6-7):727-735.

64. Hawthorne G. Measuring social isolation in older adults: development and initial validation of the friendship scale. *Social Indicators Research.* 2006;77(3):521-548.

65. Marshall J, Booth T, Devane N, et al. Evaluating the Benefits of Aphasia Intervention Delivered in Virtual Reality: Results of a Quasi-Randomised Study. *PloS one.* 2016;11(8):e0160381.

66. Northcott S, Burns K, Simpson A, Hilari K. 'Living with Aphasia the Best Way I Can': A Feasibility Study Exploring Solution-Focused Brief Therapy for People with Aphasia. *Folia Phoniatr Logop.* 2015;67(3):156-167.

67. Vickers CP. Social networks after the onset of aphasia: The impact of aphasia group attendance. *Aphasiology.* 2010;24(6-8):902-913.

68. Herdman M, Gudex C, Lloyd A, et al. Development and preliminary testing of the new five-level version of EQ-5D (EQ-5D-5L). *Quality of life research.* 2011;20(10):1727-1736.

69. Palmer R, Cooper C, Enderby P, et al. Clinical and cost effectiveness of computer treatment for aphasia post stroke (Big CACTUS): study protocol for a randomised controlled trial. *Trials.* 2015;16(1):1.

70. Forster A, Dickerson J, Young J, et al. A cluster randomised controlled trial and economic evaluation of a structured training programme for caregivers of inpatients after stroke: the TRACS trial. *Health Technology Assessment.* 2013;17(46):1-98.

71. NHS Health Scotland, University of Warwick, University of Edinburgh. Warwick Edinburgh Mental Well Being Scale. 2006.

72. Bakas T, Champion V. Development and psychometric testing of the Bakas Caregiving Outcomes Scale. *Nurs Res.* 1999;48(5):250-259.

73. Bakas T, Champion V, Perkins SM, Farran CJ, Williams LS. Psychometric testing of the revised 15-item Bakas Caregiving Outcomes Scale. *Nurs Res.* 2006;55(5):346-355.

74. Bakas T, Farran CJ, Austin JK, Given BA, Johnson EA, Williams LS. Stroke caregiver outcomes from the Telephone Assessment and Skill-Building Kit (TASK). *Top Stroke Rehabil.* 2009;16(2):105-121.

75. King RB, Hartke RJ, Denby F. Problem-solving early intervention: A pilot study of stroke caregivers. *Rehabilitation Nursing.* 2007;32(2):68-84.

76. Schwarzer R, Jerusalem M. Generalized Self-Efficacy scale. In: Weinman J, Wright S, Johnston M, eds. *Measures in health psychology: A user's portfolio. Causal and control beliefs* Windsor, UK: NFER-NELSON; 1995:35-37.

11. Appendixes

11.1 Appendix 1 – Development phase

DEVELOPMENT PHASE

This phase consists of a series of workshops with people with aphasia. Six people with aphasia (hereafter consultants) who have experience of the Connect befriending scheme, either as peer befrienders (n=3) or befriendees (n=3) will participate in the workshops. The consultants will attend six group workshops (see flowchart below) that will be conducted prior to the RCT. Each workshop will last 3 hours (in the middle of the day). The first two workshops will: (i) review information sheets and consent forms; and (ii) the outcome measures to be used and provide feedback on relevance, acceptability, format and layout. Outcome measures will also be trialled out individually with the consultants with aphasia to evaluate respondent burden. The remaining workshops will: (iii) finalise the intervention manual; (iv) determine the timing and mechanism for fidelity checks; (v) review the topic guide for participant interviews; and (vi) determine and document all the processes involved in the study including recruitment, training, supervision and support of the peer-befrienders, pairing of peer befrienders, assessment and management of risk, record keeping and mechanism for responding to and documenting adverse events.

DEVELOPMENT PHASE – Workshops

Appoint consultants (people with aphasia) who participate in a series of workshops

↓

Workshop 1-2:

- Review information sheets and consent forms
- Review outcomes measures

↓

Workshops 3-4

- Check topic guides for participant interviews
- Review current Connect Befriending Scheme Handbook and finalise content of training
- Check timing and mechanism for fidelity checks.
- Develop and review record sheet of intervention and fidelity checklist

↓

Workshop 5-6:

- Discuss all process including recruitment, training, supervision & support of peer befrienders, pairing of peer befrienders, assessment and management of risk, record keeping, and mechanisms for responding to and documenting adverse events.
- Generate Job Description and Person Specification for peer befrienders.

11.2 Appendix 2 – Description of outcome measures

11.2.1 Participant with aphasia outcomes

General Health Questionnaire – 12 (GHQ-12)

The GHQ-12[45] is a 12-item questionnaire extensively used as a screening tool of emotional distress for psychiatric disorders. The measure has been recommended for use for people with stroke,[15] having been used in many stroke studies[46-48] including, those that involve people with aphasia.[49,50] Previous research involving people with aphasia has established cut-offs on aphasia tests for self-completion of the GHQ-12 and estimate that 87-90% would be able to complete this measure.[49,50] The psychometric properties of the measure have been extensively tested.[45,51,52]

Depression Intensity Scale Circles (DISCS)

The DISCS is a single item scale that can be used to determine the level of emotional distress.[26] This scale is recommended for people with communication problems, cognitive deficits, and visual perception problems post-stroke.[15,27] The DISCS contains six circles, each 2cm in diameter. A person is asked to point to the circle which shows how depressed they feel. Scores on DISCS range 0-5, with a score of 0-1 indicating no/low distress and a score of ≥2 used as a cut-off for identifying depression in those with complex disabilities following brain injury.[26] DISCS had acceptable convergent validity, reliability, and responsiveness for depression in people with complex disabilities following acquired brain injury.[26]

Short Warwick Edinburgh Mental Well Being Scale (SWEMWBS)

The SWEMWBS is a 7-item questionnaire which provides an estimate of mental wellbeing.[53] Each item is scored on a scale ranging from 1 ('none of the time') to 5 ('all of the time'). A total score of 7 to 35 is obtained, which is then transformed into an interval scale score using a conversion table (goo.gl/hPMEC6). Higher scores reflect greater overall mental wellbeing. While there is a longer 14-item WEMWBS, the shortened version better meets the scaling properties of the Rasch model and the shortened version is highly correlated with the longer 14-item WEMWBS.[54]

Communication Participation Item Bank (CPIB)

The CPIB is a 10-item questionnaire that assesses the extent with which a person's condition interferes with their ability to participate in a range of speaking situations in the community.[55] The measure was specifically designed for people with communication difficulties, including aphasia. Each item describes a situation in which a person needs to speak with others (e.g. 'communicating in a small group of people') and asks a person to consider the degree to which their condition interferes, on a 4-point scale ranging from 0 'very much' to 4 'not at all'. A summary score range is 0 to 30, where higher scores indicate that the communication difficulties interfere less with participation. There is preliminary data on the validity of the measure[55] including, for people with aphasia.[56]

Community Integration Questionnaire (CIQ)

The CIQ is a 15-item questionnaire[57,58] that assesses community integration across three domains: home (e.g. meal preparation, housework); social (e.g. shopping, visiting friends, leisure activities); and productive activity (e.g. work, school, volunteer activities). Most items are scored on a scale ranging from 0 to 2, with total scores ranging from 0 to 29. Higher scores indicate greater independence and community integration. Most studies that have used the CIQ involved people with traumatic brain injury and shown to have good reliability and validity.[57-60] More recently, an adapted aphasia-friendly version of the CIQ was created for people with aphasia post-stroke and shown to have good internal consistency ($\alpha = 0.75$), excellent test-retest reliability (ICC = 0.96) and construct validity.[61]

Communication Confidence Rating Scale for people with aphasia (CCRSA)

The CCRSA is a 10-item questionnaire developed for people with aphasia to assess confidence in communicating in a range of activities.[62,63] Each item is scored on a 0-100 scale where 0 is 'not confident', 50 is 'moderately confident' and 100 is 'very confident'. Each score is then given a corresponding point value: 1 for 0, 10 and 20; 2 for 30, 40 and 50; 3 for 60, 70 and 80; and 4 for 90 and 100. A total score of 10-40 is used to rate a person's overall confidence in communicating, where a higher score indicates greater communicative confidence. Some preliminary evidence exists on the reliability, validity of the measure using Rasch analysis, and sensitivity to change.[62,63] Although further research on the measure is needed, it is the only published questionnaire that measures confidence for people who have aphasia post-stroke.

Friendship Scale (FS)

The FS is a brief 6-item questionnaire that measures perceived feelings of social isolation and social connection.[64] Each item is scored on a 5-point Likert scale (range= 0-4) and the possible range for the total summary score if 0 to 24, where higher scores indicate that a person is more socially connected and a score of '0' complete social isolation. The scale has been used in several studies involving people post-stroke, including people with aphasia.[65-67] It also has good reliability and discriminant validity.[64]

EQ-5D-5L

This questionnaire is a standardised instrument for measuring generic health status applicable to a wide range of conditions and treatments.[68] The first part of the measure contains 5 dimensions (mobility, self care, usual activities, pain/discomfort, anxiety/depression) that are rated on 5 levels (no problems, slight problems, moderate problems, severe problems, and extreme problems). The second part is a Visual Analogue Scale where a person self-rates their health on a 20cm vertical scale with the endpoints 'the best health you can imagine' and 'the worst health you can imagine'. The EQ-5D-5L will help to measure quality adjusted life years (QALYs) gained in each group, which will be used as an outcome in cost-effectiveness analyses and economic evaluation. A similar procedure is being used in another study underway investigating a treatment for people with aphasia post-stroke.[69]

Client Service Receipt Inventory (CSRI)

The CSRI is a well-used tool for collecting resource use in order to describe patterns and overall service use (i.e. health, social and voluntary services, informal costs etc). It also asks about the types of psychological care provided, which the research team recognise as particularly important. It is a well-respected tool used fairly routinely in RCTs. This study will use a modified CSRI data collection instrument, which has been validated for use with stroke patients.[70] To reduce burden on people with aphasia, this modified CSRI will be completed with data collected from a significant other as the primary respondent, with additional information drawn from community rehabilitation teams and records by our link clinicians.

11.2.2 Significant others outcomes

Warwick Edinburgh Mental Wellbeing Scale (WEMWBS)

The WEMWBS is a 14-item questionnaire that measures mental wellbeing, in which all items are positively worded and address aspects of positive mental health.[71] Each item is scored on a scale ranging from 1 (‘none of the time’) to 5 (‘all of the time’) resulting in a total score of between 14 to 70. A higher score indicates greater overall mental wellbeing. This scale has been shown to be reliable, valid and responsive.[54]

General Health Questionnaire-28 (GHQ-28)

The GHQ-28 is a 28-item questionnaire used to screen psychological wellbeing and detect possible cases of people with psychiatric disorders.[45] The GHQ-28 will be scored 0 0 1 1 and summed, resulting in a 0-28 score range. The questionnaire is reliable and valid.[45] The GHQ-28 also provides four subscale scores (somatic symptoms, anxiety/insomnia, severe depression, and social dysfunction)

Bakas Caregiver Outcome Scale (BCOS)

The BCOS is a 15-item questionnaire originally designed to measure life changes in family caregivers of stroke survivors, including social functioning, subjective well-being and physical health.[72] Items are rated on a 7-point scale ranging from ‘changed for the worst’ to ‘changed for the best’. A total score ranges from 15 to 105 where higher scores indicate more positive caregiver outcomes. The BCOS has satisfactory reliability and is valid[73] and has been used in several intervention studies for caregivers of stroke survivors.[74,75]

See description above for the following outcomes:

Client Service Receipt Inventory (CSRI)

11.2.3 Peer befriender outcomes

General Self-Efficacy Scale (GSE)

The GSE is a 10-item questionnaire[76] that asseses a general sense of perceived self-efficacy with the aim to predict coping with daily hassles as well as adaptation after experiencing all kinds of stressful life events. Each item is scored on a 4-point scale with responses summed to give a total score of 10 to 40 where a higher score reflects more self-efficacy. The scale has been shown to be reliable and valid.[76]

<u>See description above for the following outcomes:</u>

Community Integration Questionnaire (CIQ)

Warwick Edinburgh Mental Wellbeing Scale (WEMWBS)

General Health Questionnaire-12 (GHQ-12)

11.3 Appendix 3 – Fidelity checklists

Fidelity criteria for training workshops

**Name of rater:**	**Date of rating:**
**Details of session observed:**	**Fidelity rating score:** **/ 24**

Content covered	Present	Absent
**1. Required resources available**<br><br>[Pens and paper, name badges, local maps, hand out sheets]		
**2. Participants given opportunity to introduce themselves**<br><br>e.g. who they are, where they are from, why they have attended the training		
**3. Participants offered the chance to raise burning questions at outset of training and any issues addressed appropriately**<br><br>e.g. any fears, worries, anxieties they have about befriending		
**4. Overview of the befriending project**<br><br>e.g. its aims		
**5. Group discussion of what befriending involves**<br><br>e.g. previous experience of befriending including volunteering, personality type, the support available to befrienders, understanding the role of befriender		
**6. Discussion around the hopes and fears of the befrienders**<br><br>e.g. possible benefits and possible anxieties/concerns		
**7. Group given outline of their responsibilities as volunteers and what they might expect**		
**8. Volunteer agreement documentation shown to befrienders (and discussed if required)**		
**9. Personal risk to befrienders discussed**<br><br>Including Susie Lamplugh; individuals' journey to the training session; the laws and morals around risk; some examples of risks associated with befriending; how individuals are protected		
**10. Safeguarding discussed**<br><br>Befrienders given opportunity to discuss issues raised e.g. what might increase risk to people with aphasia, advice to say something if they see something, recognising different types of abuse, how to deal with disclosure of abuse, procedure to follow		
**11. Key purposes and aims of befriending covered**<br><br>Including the importance of discussing with befriendee what they want, and reminder that their experience and that of the befriender may not be the same		

**12. Trainer has a conversation with a volunteer, observed by the rest of the group**		
**13. Group discussion of expressive, listening and conversation skills**<br><br>Discussion of expressive skills (talk, gesture, writing, drawing, intonation, pointing); discussion of listening skills (paying attention, eye contact, responding to what is said). Discussion of conversation skills (taking turns, sharing topics, spontaneity, giving and getting ideas, humour, natural flow)		
**14. Discussion (in pairs or as part of a group) of what aspects of conversation individuals find easy and more challenging**		
**15. Variety of communication resources presented and discussed**		
**16. Group offered opportunity to raise and discuss specific anxieties regarding communication**		
**17. Roles: similarities and differences between befriender and advisor, friend and healthcare professional discussed, including the skills and knowledge each role involves**		
**18. Advice given on what a befriender should not do**<br><br>May include: involvement in personal situations, agreeing to impractical arrangements, thinking about unwanted extra contact/tasks, difficult situations		
**19. Next steps after training discussed**<br><br>May include: SUPERB team meeting people with aphasia, finding out their preferences and anxieties, arranging the first visit, giving befrienders information on the person they are matched with		
20. **Information on supervision meetings and support covered**		
**21. Visit record form covered**<br><br>May include: content of the form, easiest way of completing the form		
**22. Camera operation instructions given**		
**23. Small group work to discuss challenging scenarios**<br><br>May include: someone with very limited talk, when befriending visits come to an end, being asked for advice; group may also discuss as a whole		
**24. Recap, summary and/or opportunity for questions offered**		
**TOTAL**		

Fidelity criteria for supervision sessions

**Name of rater:**	**Date of rating:**
**Details of session observed:**	**Fidelity rating score:**

What behaviours from befrienders do we expect to see within a supervision session?

These behaviours should be present 50% of the time in 75% of the participants. We do not expect all participants to do this 100% of the time.

Behaviour	Present	Absent
1. **An ability to respect an environment suitable for frank, confidential discussion and to work collaboratively with the supervisor and peers**		
2. **An ability to communicate with and work with different individuals within the group**		
**3. An ability to listen to each other**<br><br>e.g. Did they attend, have eye contact, body posture.		
4. **An ability to be open to considering different ideas and experiences within the group**		
**5. An ability to be willing to flag up issues and discuss them openly within the group***		
**6. An ability to acknowledge the contributions of others**<br><br>e.g. Did they use appropriate / encouraging gesture, use nodding, intonation? Did they summarise or re-cap?		
**7. If risk and safeguarding was discussed, an ability to report adherence to the training guidelines (put N/A if not applicable)**<br><br>e.g. risk assessment, buddy list and itinerary, vigilance to safeguarding		
**TOTAL**		

*Items 5 and 12 apply once befrienders have been matched. If no matching has occurred, write N/A.

What behaviours do we expect to see from the supervisor in a supervision session?

**Behaviour**	**Present**	**Absent**
**8. An ability to be prepared for the session**<br><br>e.g. room set-up, resources prepared, clear overview of what the session would cover		
9. **While maintaining professional boundaries, an ability to show appropriate levels of warmth, concern, confidence and genuineness, matched to the befrienders need**		
**10. A capacity to form a collaborative relationship with the group of befrienders, based on an active stance which focuses on enabling the group members to work as a team**<br><br>e.g. did the supervisor **look and watch** the group members to 'read' what was happening within the group? Did the supervisor facilitate **balanced peer interaction and exchange?** Did the supervisor **clarify** and **check things out**? Did the supervisor **link** group members? Did the supervisor **probe, or challenge group members to** appropriately **expand the discussion**		
**11. An ability to model and actively encourage the use of total communication strategies**<br><br>e.g. use of gesture, written word, drawing, photographs, have paper and pen available, emphasise key words		
**12. An ability to accommodate and sensitively problem solve issues raised by the befrienders, or which became apparent during supervision***		
**13. A capacity to structure the session and maintain appropriate pacing**		
**14. An ability to summarise and end the group effectively**<br><br>e.g. summarise the session, discuss next steps with befrienders, agree the next meeting date		
**TOTAL**		

*Items 5 and 12 apply once befrienders have been matched. If no matching has occurred, write N/A.

Fidelity criteria for peer befriender visits

Name of rater:	Date of rating:
**Details of session observed:**	**Fidelity rating score:**

What general behaviours do we expect from befrienders within a visit?

Behaviour	Present	Absent
**1. An ability to initiate a discussion/interaction with the befriendee?**<br><br>e.g. able to create an environment suitable for open exchange and interaction or frank, confidential discussion; able to share own, post-stroke experiences; able to respond to befriendees who express concerns		
2. **While maintaining volunteer boundaries, an ability to show appropriate levels of warmth, concern, confidence and genuineness, matched to befriendees needs**		
**3. An ability to avoid negative interpersonal behaviours (such as impatience, aloofness, or insincerity)**		
4. **An ability to effectively manage the physical and social environment to be conducive to the session**		
**5. An ability to give support relevant to the befriendee which may include tips and ideas about additional resources** e.g. local support groups		
**6. An ability to share as a befriender: shared experience and share tips and ideas**		
**7. An ability to manage expectations of the visits (<u>put N/A if not applicable</u>)**<br><br>e.g. ability to communicate the frequency and duration of visits, ability to manage endings including an ability to say goodbye		
**TOTAL**		

Does the befriender show an ability to acknowledge the competence of the person with aphasia during the visit?

Behaviour	Present	Absent
**8. The conversation is natural, non-patronising and sensitive to the needs of the PWA**<br><br>e.g. non-patronising (i.e. loudness, tone of voice, appropriate pacing); appropriate emotional tone/use of humour; encourage where appropriate		
**9. Demonstrates a "listening attitude" to show active listening skills of the PWA.**<br><br>e.g. non-verbal (e.g. gesture, nods); checks and verifies the accuracy of the information being provided by the PWA; summarises and reflects on information given by PWA		

**10. An ability to listen to the PWA's thoughts and concerns in a manner which is nonjudgmental, supportive and sensitive, and which conveys a comfortable attitude when the client describes their behaviour and experience**		
11. **An ability to respond to, and openly discuss, topics or feedback raised by the PWA, that is handled sensitively and respectfully, and acknowledges moments of frustration or upset (e.g. "I know you know what you want to say").**		
**12. An ability to use humour judiciously, understanding how it can be used as an aid to help clients (e.g. to normalise the client's experience or to reduce tension), but also recognising its risks (e.g. of invalidating the client's feelings, acting as a distraction to/ avoidance of feelings, or creating "boundary violations") (put N/A if not applicable)**		
**13. An ability to respond to client's humour in a manner that is congruent with its intent, and responsive to any implied meanings (put N/A if not applicable)**		
**TOTAL**		

Does the befriender show an ability to reveal the competence of the person with aphasia during the visit?

**Behaviour**	**Present**	**Absent**
**14. The befriender has an ability to communicate in a manner that ensures the PWA understands**<br><br>e.g. (as appropriate) short, simple sentences; use of gesture; used to emphasise or clarify; wrote key words; used resources (e.g. photographs, drawings); drew pictures to illustrate key information		
**15. Opportunities are provided for the PWA to say something**<br><br>e.g. (as appropriate) verbal information (e.g. used of fixed choice and yes/no questions); models non-verbal response mode (e.g. pointing, thumbs up/down); provides written choices for pointing, clear and visible, appropriate key words; encourages PWA to write and ensure they have pen and paper; encourages the PWA to draw		
**16. An ability to negotiate the level and structure of the visits to the befriendees needs**<br><br>e.g. maintain appropriate pacing		
**TOTAL**		

11.4 Appendix 4 – AE, SAE and SUSAR forms

REPORT OF ADVERSE EVENT (AE)

All AEs will be reported to the trial manager and chief investigator

1. Person Completing Report

Name:	
Role in study:	Peer befriender ☐<br>Befriender facilitator ☐<br>Research assistant ☐<br>Other: ______________________ ☐
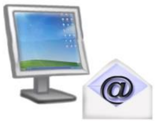<br>Email / Telephone	

2. Circumstances of event

Date of event:	
Location:	

What happened?	
Was the event **related** to the study?	☐ Yes ☐ No
Action<br>(what did you do?)	
Outcome:<br>Resolved ☐<br>Further action required ☐	Details…<br><br>Date of resolution:

5. Declaration

Signature of person completing report:	
Date:	

Signed original to be sent to Trial Manager

6. Acknowledgement of receipt by Trial Manager or Chief Investigator:

I [ ] acknowledges receipt of the above.

Signed:	
Name:	
Position:	
Date:	

REPORT OF SERIOUS ADVERSE EVENT (SAE)

All SAEs will be reported to the chief investigator and Data Monitoring Committee immediately by the trial manager.

1. Person Completing Report

Name:	
Role in study:	Peer befriender ☐<br>Befriender facilitator ☐<br>Research assistant ☐<br>Other: ____________________ ☐
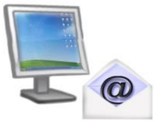<br>Email / Telephone	

2. Type of event

Please categorise this event, ticking all appropriate options:

Death ☐	Life threatening ☐	Hospitalisation or prolongation of existing hospitalization ☐
Persistent or significant disability or incapacity ☐	Congenital anomaly or birth defect ☐	Other ☐

2. Circumstances of event

Date of event:	
Location:	
What happened?	
Was the event **related** to the study?<br>Was the event **unexpected**?	☐ Yes ☐ No<br>☐ Yes ☐ No
Action<br>(what did you do?)	
Outcome:<br>Resolved ☐<br>Further action required ☐	Details…<br>Date of resolution:

5. Declaration

Signature of person completing report:	
Date:	

Signed original to be sent to Trial Manager

6. Acknowledgement of receipt by Trial Manager:

I [ ] acknowledges receipt of the above.

Signed:	
Name:	Nicholas Behn
Position:	Trial Manager
Date:	

6. Acknowledgement of receipt by Chief Investigator:

I [ ] acknowledges receipt of the above.

Signed:	
Name:	Katerina Hilari
Position:	Chief Investigator
Date:	

7. Acknowledgement of receipt by Data Management Committee

The [ ] Data Management Committee acknowledge receipt of the above.

Signed:	
Name:	
Position on DMC:	
Date:	

REPORT OF SUSPECTED UNEXPECTED SERIOUS ADVERSE REACTION (SUSAR)

The attached SAE has been classified as a SUSAR by the Chief Investigator

Details of SUSAR

Person who reported SUSAR	
Location of SUSAR	
Date of SUSAR	
Email:	

The Chief Investigator has reported this SUSAR to the Data Management Committee and sponsor of the study on the [insert date]

Signed:	
Name:	KATERINA HILARI
Date:	
Role:	Chief Investigator

Acknowledgement of receipt by main REC (please insert name):

The [ ] Research Ethics Committee acknowledges receipt of the above.

Signed:	
Name:	
Position on REC:	
Date:	

Signed original to be sent back to Chief Investigator (or other person submitting report)
Copy to be kept for information by main REC.

11.5 Appendix 5 – Example consent form

Adjustment with aphasia after stroke Research Project

Consent form – Person with aphasia

Please **tick** each box if you agree

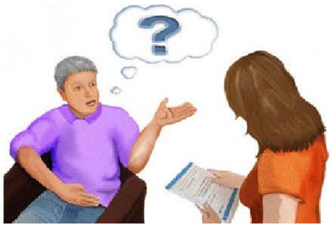

I have **read** the **information** about the **research** and had the **chance** to **ask questions** ☐

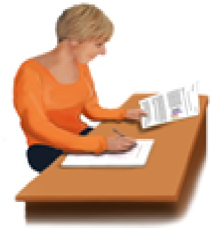

I understand that relevant **information** from my **medical records** will be passed onto the researchers ☐

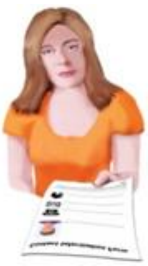

I understand that taking part means:

- Complete **questionnaires** about my **feelings** and **how I get on** ☐
- Having a **discussion** with the **researcher** ☐

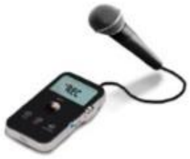

- Allow the **discussion** to be **audiotaped** ☐

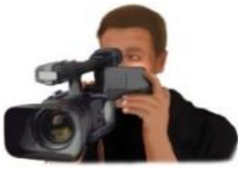

- Allow **parts of the sessions** to be **videotaped** ☐

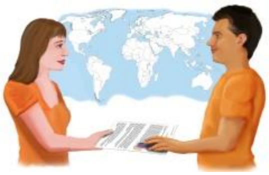

I understand that the researchers may **share my information with other researchers.** The information will be **anonymous**. They will take out my personal details first. ☐

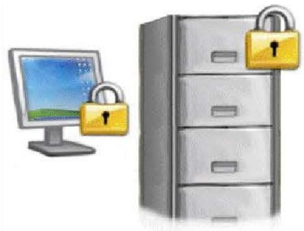

I understand that **information** about me will be **kept safe** ☐

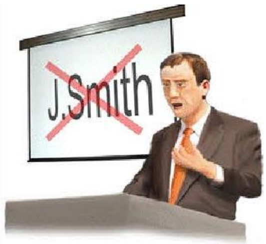

I know that when **results** are shared the researcher will **not use my name** ☐

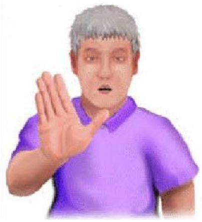

I understand that **I can stop being in the research** at any time ☐

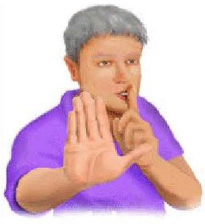

If I stop I do not have to **give a reason** ☐

…and I will still **get my normal care**

I agree to City, University of London **recording and processing this information** about me.

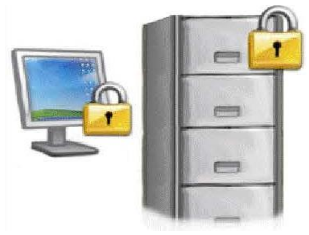

I understand that this information will be used only for the purpose(s) set out in this statement. ☐

My consent is conditional on the University complying with its duties and obligations under the **Data Protection Act** 1998.

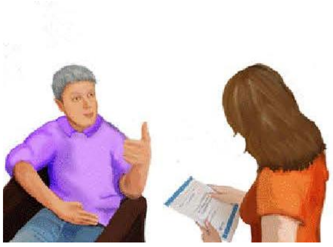

I agree to take part in the research ☐

Name ____

Signature ______________________________________ **Date** __________

I give my consent to

Name ____

Signature ______________________________________ **Date** __________
